# Determining sequencing depth in a single-cell RNA-seq experiment

**DOI:** 10.1038/s41467-020-14482-y

**Published:** 2020-02-07

**Authors:** Martin Jinye Zhang, Vasilis Ntranos, David Tse

**Affiliations:** 1 Department of Electrical Engineering, Stanford University, Stanford, CA USA; 2 Division of Biology and Biological Engineering, California Institute of Technology, Pasadena, CA USA

**Keywords:** Genome informatics, Statistical methods

## Abstract

An underlying question for virtually all single-cell RNA sequencing experiments is how to allocate the limited sequencing budget: deep sequencing of a few cells or shallow sequencing of many cells? Here we present a mathematical framework which reveals that, for estimating many important gene properties, the optimal allocation is to sequence at a depth of around one read per cell per gene. Interestingly, the corresponding optimal estimator is not the widely-used plug-in estimator, but one developed via empirical Bayes.

## Abstract (web summary)

For single-cell RNA-seq experiments the sequencing budget is limited, and how it should be optimally allocated to maximize information is not clear. Here the authors develop a mathematical framework to show that, for estimating many gene properties, the optimal allocation is to sequence at the depth of one read per cell per gene.

## Introduction

Single-cell RNA sequencing (scRNA-seq) technologies have revolutionized biological research over the past few years by providing the tools to simultaneously interrogate the transcriptional states of thousands of cells in a single experiment. In contrast to bulk RNA-Seq, which probes the average gene expression in a cell population, single-cell RNA-seq has unlocked the potential of extracting higher-order information, granting us access to the underlying gene expression distribution. Indeed, this unprecedented look into population-level heterogeneity has been vital in the success of scRNA-seq, leading up to new biological discoveries^1,2^.

**Fig. 1:**
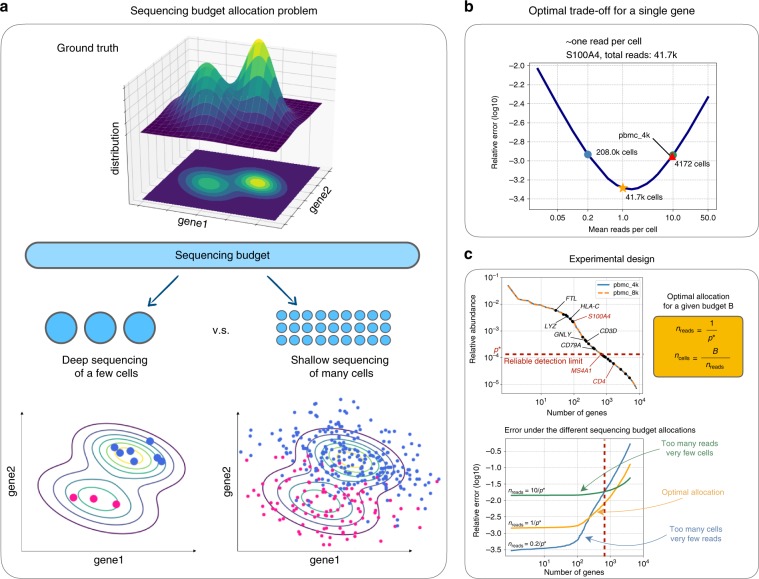
Optimal sequencing budget allocation. **a** Description of the sequencing budget allocation problem. Consider estimating the underlying gene distribution (top) from the noisy read counts obtained via sequencing (bottom). With a fixed number of reads to be sequenced, deep sequencing of a few cells accurately estimates each individual cell but lacks coverage of the entire distribution (left), whereas a shallow sequencing of many cells covers the entire population but introduces a lot of noise (right). **b** Optimal tradeoff. The memory T-cell marker gene *S100A4* has 41.7k reads in the pbmc_4k dataset. For estimating the underlying gamma distribution ${X}_{g} \sim {\rm{Gamma}}({r}_{g},{\theta }_{g})$, the relative error is plotted as a function of the sequencing depth, where the optimal error is obtained at a depth of one read per cell (orange star) and is two times smaller than that at the current depth of pbmc_4k (red triangle). **c** Experimental design. To determine the sequencing depth for an experiment, first the relative gene expression level can be obtained via pilot experiments or previous studies (top left). Then the researcher can select a set of genes of interest (i.e., some marker genes highlighted as black dots), of which the smallest relative expression level ${p}^{* }$ (*MS4A1*) defines the reliable detection limit. Finally, the optimal sequencing depth is determined as ${n}_{{\rm{reads}}}^{* }=1/{p}^{* }$ (top right). The errors under different tradeoffs are visualized as a function of the genes ordered from the most expressed to the least (bottom). The optimal sequencing budget allocation (orange) minimizes the worst-case error over all the genes of interest (left of the red dashed line), whereas both the deeper sequencing (green) and the shallower sequencing (blue) yield worse results.

Although early single-cell RNA-seq assays were labor intensive and initially constrained by the small number of cells that could be processed in a single experiment, recent technological advances have allowed hundreds of thousands of cells to be assayed in parallel^3^, eliminating the otherwise prohibitive per cell cost overhead. From a sequencing budget perspective, however, this seemingly unconstrained increase in the number of cells available for scRNA-seq introduces a practical limitation in the total number of reads that can be sequenced per cell. More reads can significantly reduce the effect of the technical noise in estimating the true transcriptional state of a given cell, whereas more cells can provide us with a broader view of the biological variability in the cell population. A natural experimental design question arises (Fig. 1a): how many cells should we choose to profile for a given study, and at what sequencing depth?

The experimental design question has attracted a lot of attention in the literature^4–8^, but as of now, there has not been a clear answer. Several studies provide evidence that a relatively shallow sequencing depth is sufficient for common tasks such as cell type identification and principal component analysis (PCA)^9–11^, whereas others recommend deeper sequencing for accurate gene expression estimation^12–15^. Despite the different recommendations, the approach to providing experimental design guidelines is shared among all: given a deeply sequenced dataset with a predefined number of cells, how much subsampling can a given method tolerate? An example of this conventional approach is also evident in the mathematical model used in a recent work^11^ to study the effect of sequencing depth on PCA. Although practically relevant, this line of work does not provide a comprehensive solution to the underlying experimental design question because of three reasons: (1) the number of cells is fixed and implicitly assumed to be enough for the biological question at hand; (2) the deeply sequenced dataset is considered to be the ground truth; (3) the corresponding estimation method is chosen a priori and is tied to the experiment.

In this work, we propose a mathematical framework for single-cell RNA-seq that fixes not the number of cells but the total sequencing budget, and disentangles the biological ground truth from both the sequencing experiment as well as the method used to estimate it. In particular, we consider the output of the sequencing experiment as a noisy measurement of the true underlying gene expression and evaluate our fundamental ability to recover the gene expression distribution using the optimal estimator. The two design parameters in our proposed framework are the total number of cells to be sequenced ${n}_{\mathrm{cells}}$ and the sequencing depth in terms of the total number of reads per cell ${n}_{\mathrm{reads}}$, both affecting the optimal estimation error. Now, the experimental design tradeoff becomes apparent when these two quantities are tied together under a total sequencing budget constraint $B={n}_{\mathrm{cells}}\times {n}_{\mathrm{reads}}$ (Fig. 1a, sequencing budget allocation problem). The sequencing budget $B$ corresponds to the total number of reads that will be generated and is directly proportional to the sequencing cost of the experiment (see Methods).

More specifically, we consider a hierarchical model^16–18^ to analyze the tradeoff in the sequencing budget allocation problem (see Methods). At a high level, we assume an underlying high-dimensional gene expression distribution ${P}_{{\bf{X}}}$ that carries the biological information of the cell population we are interested in and is independent of the sequencing process (Fig. 1a top). The cells $1,2,\cdots$ in the experiment are described by gene expressions ${{\bf{X}}}_{1},{{\bf{X}}}_{2},\cdots$ sampled from ${P}_{{\bf{X}}}$, whereas we can only observe the read counts ${{\bf{Y}}}_{1},{{\bf{Y}}}_{2},\cdots$ that are generated from the corresponding gene expressions via sequencing (Fig. 1a bottom). In this context, it is clear that with many cells ${n}_{\mathrm{cells}}$ we can estimate the read count distribution ${P}_{{\bf{Y}}}$ accurately, whereas with more reads per cell ${n}_{\mathrm{reads}}$ we can make sure that the individual (normalized) observations ${{\bf{Y}}}_{1}/{n}_{\mathrm{reads},1},{{\bf{Y}}}_{2}/{n}_{\mathrm{reads},2},\cdots$ are much closer to the ground truth expressions ${{\bf{X}}}_{1},{{\bf{X}}}_{2},\cdots$ of the cells (here, ${n}_{\mathrm{reads},c}$ represents the total number of reads for cell $c$ and the average of ${n}_{\mathrm{reads},c}$ over all cells is ${n}_{\mathrm{reads}}$). The optimal tradeoff is then derived to reconcile the two.

## Results

### Model overview

The gene expression levels of each cell, denoted by ${{\bf{X}}}_{c}=[{X}_{c1},\cdots \ ,{X}_{cG}]$ for $c=1, \ldots ,{n}_{\mathrm{cells}}$, can be viewed as independent samples from the gene expression distribution ${P}_{{\bf{X}}}$, where $G$ denotes the number of genes. More specifically, we assume that ${X}_{cg}$ represents the true relative abundance of the mRNA molecules originating from a gene $g$ in cell $c$, so that ${\sum }_{g=1}^{G}{X}_{cg}=1$. To model the sequencing process, we assume that after a particular cell ${{\bf{X}}}_{c}$ has been sampled from ${P}_{{\bf{X}}}$, its corresponding gene counts ${{\bf{Y}}}_{c}=[{Y}_{c1},\cdots \ ,{Y}_{cG}]$ are generated via Poisson sampling of ${\gamma }_{c}\cdot {n}_{\mathrm{reads}}$ reads from ${{\bf{X}}}_{c}$, where ${\gamma }_{c}$ is a size factor that is cell-specific but not gene-specific. Overall, our hierarchical model is given by (here, we simplified the model by fixing ${\gamma }_{c}$; see Eq. (2) in the Methods section for the complete model and Supplementary Note 1 for more details): for cells $c=1,2, \cdots ,{n}_{\mathrm{cells}}$,

1 $${{\bf{X}}}_{c} \sim {P}_{{\bf{X}}},\quad {\rm{and}}\quad {Y}_{cg}| {X}_{cg} \sim {\rm{Poi}}({\gamma }_{c}{n}_{{\rm{reads}}}{X}_{cg})\,{\rm{for}}\,g=1,2,\cdots ,G.$$

Under this framework, the ultimate goal of a single-cell RNA-seq experiment would be to estimate quantities related to the (unknown) ground truth distribution ${P}_{{\bf{X}}}$ from the noisy measurements ${{\bf{Y}}}_{c}$. Fixing the total sequencing budget $B={n}_{\mathrm{cells}}\times {n}_{\mathrm{reads}}$, we aim to characterize the optimal experimental design tradeoff between the number of cells ${n}_{\mathrm{cells}}$ and the number of reads per cell ${n}_{\mathrm{reads}}$ that can minimize the corresponding estimation error.

Although our framework is non-parametric—in the sense that no particular prior is assumed for the underlying gene distribution ${P}_{{\bf{X}}}$, it is instructive to illustrate the framework in the context of the widely used overdispersion model, where for each gene $g$, the read counts ${Y}_{cg}$ are assumed to follow a negative binomial distribution^19–21^. As the negative binomial distribution can be derived as a gamma–Poisson mixture, the resulting model can be viewed as a special case of Eq. (1) in which the underlying gene expression marginals follow gamma distributions. In that case, one would be interested in estimating the marginals ${X}_{g} \sim {\mathrm{Gamma}}({r}_{g},{\theta }_{g})$, effectively decoupling the true biological variability from the technical noise that was introduced during sequencing via Poisson sampling (see Relation to the overdispersion model in Supplementary Note 4).

As a technical remark, for assuming the gamma–Poisson mixture, we dropped two constraints without loss of generality, i.e., ${X}_{cg}\;<\;1$ and ${\sum }_{g=1}^{G}{X}_{cg}=1$. The former is because the relative expression ${X}_{cg}$ is of the order of $1/G$, which is much smaller than 1. With a mean much smaller than 1, the truncated gamma distribution with truncation at 1 is very close to the corresponding untruncated distribution. The latter is because that the number of genes $G$ is large, and therefore, ${\sum }_{g=1}^{G}{X}_{cg}$ concentrates around its mean, which is 1.

### Optimal sequencing budget allocation

For our main results, we focused on 3’-end sequencing technologies^22–24^ and used the above framework to study the experimental design tradeoff for estimating several important quantities of the underlying gene distribution, such as the CV and the Pearson correlation (see the Methods section). In the context of 3’-end sequencing, ${P}_{{\bf{X}}}$ naturally models the unknown high-dimensional distribution of mRNA abundances across cells, whereas the read counts for the cells, ${{\bf{Y}}}_{1},{{\bf{Y}}}_{2},\cdots$, correspond to the number of unique molecular identifiers (UMIs) observed via sequencing. Our main result states that the optimal budget allocation (i.e., the one that minimizes the estimation error) is achieved by maximizing the number of cells while making sure that at least ~1 UMI per cell will be observed on average for all genes of primary biological interest in the experiment.

As a demonstrating example, in Fig. 1b we consider the memory T-cell marker gene *S100A4* to be of primary biological interest and evaluate the optimal tradeoff in the context of the overdispersion model for the total sequencing budget used to generate the 10x Genomics’ pbmc_4k dataset (4340 cells, total 41.7 k reads for *S100A4*); our analysis suggests that the optimal tradeoff would have been attained by sequencing 10 times shallower using 10 times more cells, reducing the error by twofolds. Of course, the recommended sequencing depth depends on the genes under consideration. For example, the sequencing depth of pbmc_4k dataset is optimal when the B-cell marker gene *MS4A1* is considered, and it should be sequenced four times deeper with 1/4 cells when the T-helper marker gene *CD4* is considered (Fig. 1c top, Supplementary Fig. 2a, b). The latter arguably has reached saturation for the 10x Genomics’ technology. Hence, the guidance there is to sequence until saturation, i.e., sequence until no more new UMIs are observed (see Experimental design in the Methods section as well as Supplementary Note 3).

As the example indicates, an important aspect of our framework is to allow flexible experimental design at a single-gene resolution. The researcher can thus design the experiment based on the mean expression level of a set of important genes related to the biological question, where the mean expression level can be obtained via pilot experiments or previous studies (see Experimental design in the Methods section). We illustrate the proposed experimental design procedure by considering peripheral blood mononuclear cells (PBMCs) with the corresponding marker genes (Fig. 1c). The goal is to ensure reliable estimation for all these genes that are above a certain expression level, say that of *MS4A1*. Hence, the expression level of the gene of interest, i.e., *MS4A1*, naturally defines the reliable detection limit ${p}^{* }$ at which we should guarantee observation of one average UMI per cell. Thus, given a budget $B$, choosing ${n}_{\mathrm{reads}}^{* }=1/{p}^{* }$ and ${n}_{\mathrm{cells}}^{*}=B/{n}_{\mathrm{reads}}^{*}$ achieves the optimal tradeoff for reliable detection at ${p}^{* }$. In this example, *MS4A1* will be sequenced ~1 UMI per cell on average. Interestingly, this approach suggests a slightly deeper sequencing for current 10x datasets (Supplementary Figs. 1 and 2).

**Fig. 2:**
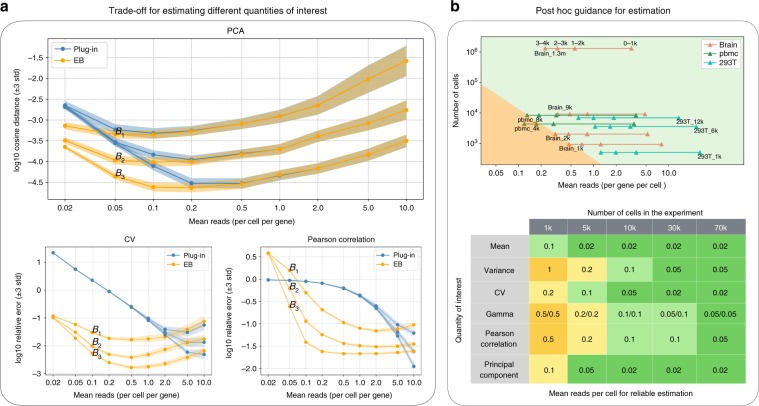
Empirical quantification of the optimal sequencing depth. **a** Simulations of error under different budget allocation. 3-std confidence intervals are provided. The top panel simulates the error for estimating the first principal direction using the plug-in estimator (blue) and the EB estimator (orange), respectively. Three budgets are considered, i.e., ${B}_{1}$ = 0.6  k per gene, ${B}_{2}$ = 3k per gene, ${B}_{3}$ = 15k per gene. The depth (mean reads per cell per gene) ranges from 0.02 to 10. The result indicates that the optimal depth for the EB estimator is the same (~0.1) for all three budgets, validating the theory that the optimal depth is independent of the budget. The cases for the coefficient of variation and the Pearson correlation (bottom) also show similar qualitative behaviors. **b** Post hoc guidance for reliable estimation. We visualized the top 4k genes of some representative datasets (top), where a triangle residing in the green region means the Pearson correlation of corresponding genes can be reliably estimated (relative error < 10%). For example, we can reliably estimate the first 2k genes for the brain_1k dataset and all 4k genes for the brain_9k dataset. A more comprehensive result is summarized in the bottom table. For example, the first element (mean, 1k) shows that with 1k cells, a gene needs to have at least 0.1 reads per cell for reliably estimating the mean.

Unlike estimating the gamma distribution parameters for the overdispersion model, we considered estimating other quantities in a non-parametric setting (see also a non-parametric interpretation of the overdispersion model in Relation to the overdispersion model in Supplementary Note 4). Although the exact optimal depth is task-dependent, our empirical evaluations have shown that the above recommendation is remarkably consistent across all quantities considered in this paper—typically lying in a narrow range between 0.2 and 1 (Fig. 2a, Supplementary Fig. 4). Last but not the least, our tradeoff analysis can also provide a post hoc guidance for reliable estimation for existing datasets, namely for certain quantities, to determine which genes can be reliably estimated and which cannot, based on their mean expression level (Fig. 2b, see also post hoc guidance for reliable estimation in Methods).

### Optimal estimator

Another important result arising from our experimental design framework is the fundamental role of the estimator in the optimal tradeoff. A very common—almost routine—practice in the literature is to use the so-called plug-in estimator, which, as a general recipe, blindly uses the scaled (relative) read counts ${{\bf{Y}}}_{1}/{n}_{\mathrm{reads},1},{{\bf{Y}}}_{2}/{n}_{\mathrm{reads},2},\cdots$ as a proxy for the true relative gene expression levels ${{\bf{X}}}_{1},{{\bf{X}}}_{2}\cdots$, effectively estimating the corresponding distributional quantities by plugging-in the observed values. For example, the plug-in estimator naturally estimates the mean of the gene expression distribution ${P}_{{\bf{X}}}$ by that of ${P}_{{\bf{Y}}/{n}_{\mathrm{reads}}}$, the variance of ${P}_{{\bf{X}}}$ by that of ${P}_{{\bf{Y}}/{n}_{\mathrm{reads}}}$, etc. This approach, although very accurate for deeply sequenced datasets, becomes increasingly problematic in the limit of shallow sequencing; overdispersion and inflated dropout levels in lowly expressed genes, typically associated in the literature with scRNA-seq, are some of the more pronounced consequences.

For the sequencing budget allocation problem, we did not restrict our results to any particular estimator; our analysis suggested that the optimal tradeoff cannot be achieved by the conventional plug-in approach but with another class of estimators developed via empirical Bayes modeling^16–18,25,26^ (see Methods). Such estimators are inherently aware of the Poisson sampling noise introduced by sequencing, and therefore can adapt to various sequencing depths. As they estimate the prior gene distribution ${P}_{{\bf{X}}}$ in the hierarchical model (2) from the observed data ${{\bf{Y}}}_{c}$, sometimes by estimating the moments of the prior distribution ${P}_{{\bf{X}}}$, they are usually associated with the names empirical Bayes, moment matching, or density deconvolution. Here, we use the term EB to refer to them in general.

**Fig. 3:**
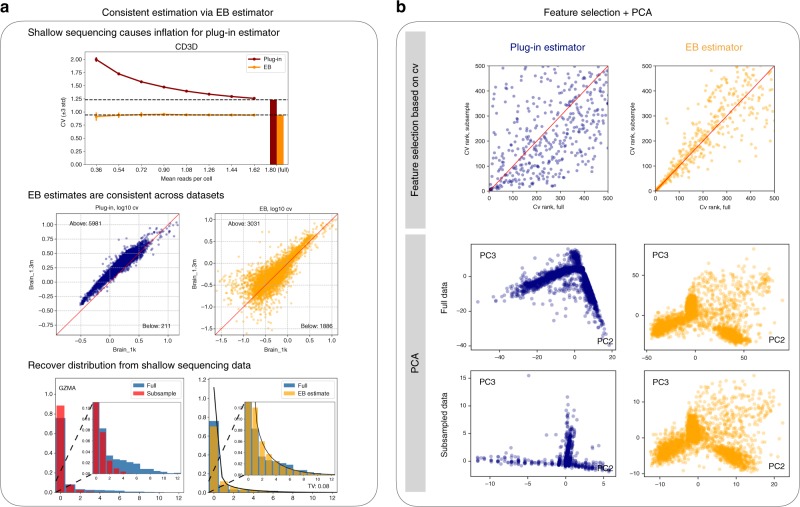
EB estimates are consistent between deep and shallow datasets. **a** Top: for estimating the coefficient of variation (CV), the plug-in estimates become more inflated as the sequencing depth becomes shallower (from right to left along the *x* axis), whereas the EB estimates are consistent. 3-std confidence intervals are provided for this panel. Middle: both brain_1k and brain_1.3m are from the mouse brain, and hence each gene should have a similar CV value between the two datasets. This is indeed the case for the EB estimator (right), which is adaptive to different sequencing depths. However, as brain_1k is twice deeper than brain_1.3m, the plug-in estimates are biased that most points are above the 45-degree line (red). Bottom: distribution recovery for the gene *GZMA* from a dataset that is subsampled to be five times shallower (left). The EB estimator provides a reasonable estimation for both the zero proportion and the tail shape, resulting in a small total variation error (right). **b** Feature selection and PCA. The task is to first select features (genes) based on CV, and then perform PCA on the selected features. The results on the full data (pbmc_4k) and a subsampled (three times shallower) are compared. EB estimates are more consistent between the full data and the subsampled data for both the CV ranks (top) and the PCA plots (bottom).

**Fig. 4:**
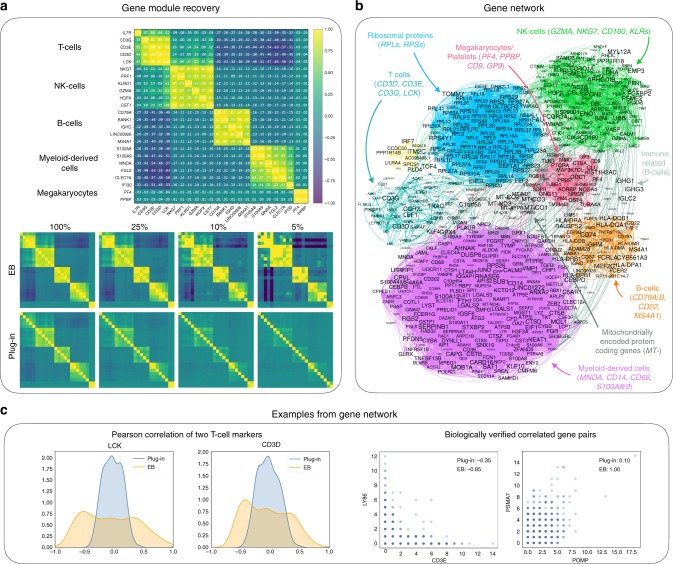
Gene module and gene network analysis. **a** Top: the EB-estimated Pearson correlation for some marker genes in pbmc_4k are visualized, ordered by different cell populations (top). The clear block-diagonal structure implies that the EB estimator is capable of capturing the gene functional groups. As a comparison, the plug-in estimator also recovers those modules but with a weaker contrast (bottom left panel, plug-in with 100%). Bottom: a subsample experiment further shows that the EB estimator can recover the module with 5% of the data. For the plug-in estimator, the first block (T cells) is blurred with 25% of the data, and the entire structure vanishes with 10% of the data. **b** Gene network based on the EB-estimated Pearson correlation using the pbmc_4k dataset. Most gene modules correspond to important cell types or functions, including T cells, B cells, NK-cells, myeloid-derived cells, megakaryocytes/platelets, ribosomal protein genes, and mitochondrially encoded protein-coding genes. **c** Left: the estimated Pearson correlations between all genes and *LCK* (1st panel) and *CD3D* (2nd panel), two known T-cell markers. There are three modes for the EB-estimated values, where the positive mode, the zero mode, and the negative mode correspond to genes in the same module, different modules, and irrelevant genes, respectively. The plug-in estimated values are nonetheless much closer to zero even for the truly correlated ones, indicating an artificial shrinkage of the estimated values. Right: two instances where the EB estimates are significantly different from the plug-in estimates. The axes represent read counts, and the color codes the number of cells. Both gene pairs are biologically validated (see Gene network analysis in Methods). See also Supplementary Figs. 11–12 for more examples.

In Figs. 3 and 4 (also Supplementary Figs. 7–12), we provide a comprehensive evaluation of the performance of EB estimators in several key applications and show that they provide remarkably consistent estimates across varying sequencing depths and different datasets. Also, they are shown to be biologically meaningful (Fig. 4c, Supplementary Fig. 12). In contrast, the plug-in approach, being sensitive to the sequencing depth, significantly overestimates the variability in gene expression (CV) owing to the inevitable zero-inflation occurring at shallow sequencing (Fig. 3a), and subsequently limits the performance of common downstream tasks such as PCA and gene network analysis (Methods, Fig. 3b, Fig. 4).

### Validation against the gold standard smFISH

In order to further validate our results that the optimal sequencing depth is attained at ~1 average UMI per cell, and that the EB estimates are indeed close to the ground truth, we considered two additional datasets^15,27^, accompanied by the single-molecule fluorescent in situ hybridization (smFISH) data, which is regarded as the gold standard for measuring the number of mRNAs in a cell. The libraries for the two scRNA-seq datasets were generated by Drop-seq^23^ and CEL-seq^28^, respectively, two UMI-based technologies.

**Fig. 5:**
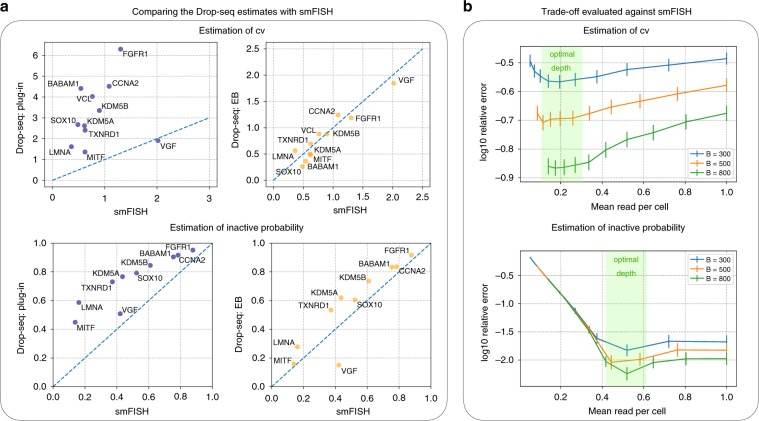
Validation using smFISH data. **a** The estimated CV (top) and inactive probability (bottom, $\kappa =2.5{n}_{{\rm{reads}}}$) from the Drop-seq data are compared with the smFISH results. The EB estimates (right) are consistent with the smFISH results while there is a clear inflation for the plug-in estimates. **b** The sequencing budget tradeoff for estimating CV (top) and inactive probability (bottom, $\kappa =1$, i.e., estimating the zero proportion at 1 read per cell) for the gene *MITF*. The relative error is evaluated against the gold standard smFISH result. 3-std confidence intervals are provided.

We first compared the estimated CV and inactive probability against the smFISH estimates. The EB estimates agree well with the smFISH data while there is clear inflation for the plug-in estimates (Fig. 5a, Supplementary Fig. 13). Furthermore, we investigated the optimal sequencing depth by fixing the budget $B$ and varying the sequencing depth, as we did in Fig. 2a. The critical difference here is that the error is evaluated against the smFISH data, which serves as a proxy for the ground truth. Two genes, *MITF* and *VGF*, were considered that have relatively more UMIs to subsample. The tradeoff curves (Fig. 5b, Supplementary Fig. 14) are qualitatively similar to the simulation studies (Fig. 2a, Supplementary Fig. 4), showing an optimal depth between 0.1 and 0.6. This is consistent with the experimental design guidelines that we provided in our earlier analysis.

See also Supplementary Figs. 15–17 and the Details of the ERCC experiments subsection in Supplementary Note 6 for additional validations using datasets with ERCC synthetic spike-in RNAs and pure RNA controls.

## Discussion

A natural yet challenging experimental design question for single-cell RNA-seq is how many cells should one choose to profile and at what sequencing depth to extract the maximum amount of information from the experiment. In this paper, we introduced the sequencing budget allocation problem to provide a precise answer to this question; given a fixed budget, sequencing as many cells as possible at approximately one read per cell per gene is optimal, both theoretically and experimentally.

Conceptually, there are three important aspects of our mathematical framework that enabled our theoretical analysis and led to the development of the corresponding sequencing-depth-aware EB estimators. First, we explicitly incorporated the notion of an unknown ground truth distribution ${P}_{{\bf{X}}}$ that describes the underlying single-cell population of interest. From this perspective, a single-cell RNA-seq experiment can be naturally seen as an attempt to extract information about this distribution. Second, we disentangled this biological ground truth not only from the sequencing process but also from the method used to estimate it. Considering the output of the sequencing experiment as a noisy measurement ${P}_{{\bf{Y}}}$ of the true underlying distribution, we were able to mathematically evaluate our fundamental ability to recover ${P}_{{\bf{X}}}$ and identify the corresponding tradeoff-optimal estimators for several quantities of interest by essentially optimizing over all possible methods and experimental design parameters. Finally, to provide practical experimental design guidelines, we considered how different biological questions could be incorporated within our framework. Assuming that a biological question can be defined in terms of a set of genes of interest (e.g., associated with a particular pathway), we were able to provide sequencing depth recommendations by minimizing the worst-case error within that set.

Our experimental results showed that the proposed EB estimators could achieve significantly better performance compared with the conventional plug-in approach that is commonly used by existing single-cell analysis methods. Importantly, we demonstrated that the proposed estimators produce unbiased results across deep and shallow datasets obtained from the same underlying population of cells and validated their ability to produce estimates that are very close to the ground truth as measured by smFISH. We also provided post hoc guidance for reliable estimation by evaluating our results on multiple genes from different biological samples. Apart from providing cost-efficient data generation guidelines for future experiments, we believe that our results are also going to be useful in assessing the quality and statistical interpretability of existing datasets, particularly in the context of global collaborative initiatives such as the Human Cell Atlas^29^.

## Methods

### Model

For a scRNA-seq experiment, let ${n}_{\mathrm{cells}}$ be the number of cells and ${n}_{\mathrm{reads}}$ be the average UMIs per cell. The total number of UMIs $B={n}_{\mathrm{cells}}\times {n}_{\mathrm{reads}}$ is used to denote the available budget for this experiment. Given a fixed budget, we are interested in the optimal allocation between ${n}_{\mathrm{cells}}$ and ${n}_{\mathrm{reads}}$ for estimating certain distributional quantities that are important to the scRNA-seq analysis.

We adopt a hierarchical model for the analysis. Let *G* be the number of genes and for each cell $c=1,\cdots \ ,{n}_{{\mathrm{cells}}}$, let ${{\bf{X}}}_{c}=[{X}_{c1},\cdots \ ,{X}_{cG}]$ be the relative gene expression level satisfying ${\sum }_{g=1}^{G}{X}_{cg}=1$. The relative expression levels are assumed to be drawn i.i.d. from some unknown cell distribution ${P}_{{\bf{X}}}$, which is defined with respect to the cell population under investigation—it may be cells from a certain tissue or some isolated cell sub-populations. This is quite a general model. For example, cells coming from several sub-populations can be modeled by letting ${P}_{{\bf{X}}}$ be a mixture distribution. The gene expression level ${{\bf{X}}}_{c}$ is measured by the observed UMIs ${{\bf{Y}}}_{c}\in {{\mathbb{N}}}^{G}$ via sequencing, of which the stochastic process is modeled using Poisson noise; such noise model has been extensively validated by previous works^16,30^. In addition, we assume a size factor ${\gamma }_{c}$ for each cell that accounts for the variation in cell sizes. To summarize, for gene $g=1,\cdots \ ,G$ in cell $c=1,\cdots \ ,{n}_{{\mathrm{cells}}}$, we have assumed

2 $$\begin{array}{ccc}&&{{\bf{X}}}_{c}\mathop{ \sim }\limits^{{\rm{i.i.d.}}}{P}_{{\bf{X}}},\ \ {\gamma }_{c}\mathop{ \sim }\limits^{{\rm{i.i.d.}}}{P}_{\gamma },\\ &&{Y}_{cg}| {X}_{cg},\quad{\gamma }_{c} \sim {\rm{Poi}}({\gamma }_{c}{n}_{{\rm{reads}}}{X}_{cg}).\end{array}$$

### Quantities to estimate

We study the optimal sequencing budget allocation for estimating the following distributional quantities of ${P}_{{\bf{X}}}$ that are commonly used in scRNA-seq analysis. See Supplementary Note 2 for more details.

- The marginal gene moments ${M}_{k,g}={\mathbb{E}}[{X}_{cg}^{k}]$, $g=1,\cdots \ ,G,\,k=1,2,\cdots$. The marginal gene moments can be used to compute quantities like the mean expression, CV, the Fano factor, or the parameters for the overdispersion model (assuming that ${X}_{cg}$ follows a gamma distribution), which play an important role in data pre-processing, feature selection, and gene-type identification^31,32^.
- The gene expression covariance matrix $K\in {{\mathbb{R}}}^{G\times G}$, which also gives rise to the Pearson correlation matrix. Both quantities can be used to study the dependency structure of genes, e.g., via spectrum methods^33–35^ or gene network analysis^36,37^.
- The inactive probability of a gene $g$ with the definition ${p}_{0,g}(\kappa )={\mathbb{E}}[\exp \left(-\kappa {X}_{cg}\right)]$. It also has the interpretation of the proportion of zero-UMI cells for gene $g$ when the cell population is sequenced $\kappa /{n}_{{\rm{reads}}}$ times deeper. As special cases, $\kappa$ = $\infty$ corresponds to the probability that ${X}_{cg}$ is zero, whereas $\kappa$ = ${n}_{{\mathrm{reads}}}$ corresponds to the proportion of cells whose observed counts ${Y}_{cg}$ are zero. The latter was also considered in a recent work^38^.
- The inactive probability of a gene pair ${g}_{1},{g}_{2}$ with the definition ${p}_{0,{g}_{1}{g}_{2}}(\kappa )={\mathbb{E}}[\exp (-\kappa ({X}_{c{g}_{1}}+{X}_{c{g}_{2}}))]$ that quantifies the change that both genes are inactive. This quantity can be used to analyze the gene co-expression network^38^.
- The marginal gene distribution ${P}_{{X}_{g}}$ (also considered in a recent work^16^).

### Optimal sequencing budget allocation

We considered a single gene and derived the optimal budget allocation for estimating all the above quantities of its distribution ${P}_{{X}_{g}}$ (see Supplementary Note 5 for more details). As the mean relative expression level of a gene ${p}_{g}$ is relatively stable within a specific tissue/sample (see Experimental design subsection below for more details), one can safely estimate that for an experiment with budget $B$, the total number of reads for gene $g$ is around ${p}_{g}B$. Then the tradeoff with respect to gene $g$ can be written as ${p}_{g}B={n}_{{\mathrm{reads}},g}\times {n}_{{\mathrm{cells}}}$, where ${n}_{{\mathrm{reads}},g}$ is the mean reads per cell for gene $g$, satisfying the relation ${n}_{{\mathrm{reads}},g}={p}_{g}{n}_{{\mathrm{reads}}}$.

**Theorem 1**. (Optimal budget allocation, informal) For estimating moments, covariance matrix, inactive probability, pairwise inactive probability, and distribution, the optimal budget allocation is

3 $${n}_{\,\text{reads}\,,g}^{* } \sim 1,\ {n}_{\,\text{cells}\,}^{* } \sim B{p}_{g}.$$

The optimality is in the sense of minimizing the worst-case error over a family of distributions ${P}_{{X}_{g}}$ with mild assumptions and the optimal error rate is achieved by the EB estimators.

The expression ${n}_{{\mathrm{reads}},g}^{* } \sim 1$ in Theorem 1 implies that the optimal sequencing depth (mean reads per cell per gene) is given by some constant independent of the sequencing budget (see the formal statement in Supplementary Note 5). Therefore, for a scRNA-seq experiment, we should aim at a certain sequencing depth; when the budget increases, we should keep the same depth and allocate the additional budget toward collecting more cells. In other words, after having achieved a certain sequencing depth, deeper sequencing does not help as much as having more cells. We also note that the actual value of this optimal sequencing depth may be different for estimating different quantities, which is further investigated in the following section. In addition, Theorem 1 suggests that the EB estimators should be used for optimal estimation, whose effectiveness is demonstrated in Figs. 3–4.

### Experimental design

The exact values of the optimal sequencing depth ${n}_{{\mathrm{reads}},g}^{* }$ for estimating different quantities were investigated both theoretically and via simulations. First, the closed-form expressions of the optimal depth ${n}_{{\mathrm{reads}},g}^{* }$ were derived for estimating the mean, the second moment, and the gamma parameters (of overdispersion model), which depend on the distribution ${P}_{{X}_{g}}$ but are nonetheless ~1 for typical cases (Supplementary Notes 3 and 5). Second, estimation errors under different budget splits were simulated by subsampling from a real dataset with deeply sequenced genes and many cells (top 72 genes of brain_1.3m, Fig. 2a). See details of the subsampling procedure in Subsampling experiment in Supplementary Note 6). Third, a more controlled simulation that assumes the Poisson model was conducted to provide a more comprehensive evaluation (Supplementary Fig. 4). Both simulations exhibit similar qualitative behaviors and imply that the optimal sequencing depths ${n}_{{\mathrm{reads}},g}^{* }$ for estimating different quantities are between 0.2 and 1. Therefore, we reached the conclusion that the optimal budget allocation for a single gene is to have ~1 UMI per cell on average.

When there are many genes of primary biological interest, the gene among them with the smallest relative mean expression level becomes the bottleneck, as it has the fewest number of reads on average (Fig. 1c, top). We call its relative mean expression level ${p}^{* }$ the reliable detection limit, below which the estimation performance cannot be guaranteed. The optimal sequencing depth for the entire experiment ${n}_{{\mathrm{reads}}}^{* }$ is chosen so that the gene at the reliable detection limit has one read per cell (in expectation), which minimizes the worst-case error for all genes of interest. Compared with this optimal allocation, deeper sequencing (green) gives a homogeneous error across genes but at a much higher level, whereas a shallower sequencing (blue) gives a small error for a few highly expressed genes but its performance quickly deteriorates (Fig. 1c, bottom).

The recommended budget allocation in general suggests a slightly deeper sequencing depth as compared with existing datasets, e.g., 7k UMIs per cell for the pbmc_4k dataset considering *MS4A1* and 14k UMIs per cell for the brain_9k dataset considering *S100a10* (Supplementary Fig. 2). Such a depth is feasible for the current 10x Genomics’ technology, which is estimated to be able to sequence 10–45k UMIs per cell where the actual values depend on different tissues (Feasibility of the recommended sequencing depth in Supplementary Note 3). In addition, under such a sequencing depth, all analyses are valid as the Poisson model is still a good approximation of the sequencing process. Regarding the rare genes, since the UMI efficiency for the 10x technology is estimated to be 10–15%, in order to achieve one read per cell, the gene needs to have at least 1/0.15$\approx$7 transcripts in the cell. The gene *CD4* (Supplementary Fig. 2b) seems to be below this limit. For such genes, the recommendation should be sequencing until saturation.

The input parameter to the proposed experimental design approach, i.e., the detection limit ${p}^{* }$, corresponds to the smallest mean expression level among the list of genes of interest. Therefore, to carry out the proposed experimental design procedure, it is important to have an estimate of the mean expression levels for these genes. Such information may come from various sources whose data closely matched the system under study. First, researchers usually conduct pilot experiments before conducting the main experiment; the data from the pilot experiment can be used to provide such an estimate. Also, data from past studies or public databases, either scRNA-seq or bulk RNA-Seq, can be used to provide the estimate. Some popular databases include Tabula Muris (scRNA-seq)^39^ for different mouse tissues, Human Cell Atlas^29^ (scRNA-seq) and GTEx^40^ (bulk RNA-Seq) for different human tissues, TCGA^41^ (bulk RNA-Seq) for human cancer data, and GEO^42^ (bulk/single-cell RNA-seq) for past studies. One caveat here is that different datasets may have different covariate compositions, like sex, age, or demographic factors. To evaluate the sensitivity of using reference data to estimate ${p}^{* }$ for the proposed experimental design procedure, we consider four different types of reference data in Supplementary Figs. 18–19: in-sample bulk RNA-Seq or scRNA-seq, where the corresponding reference data were obtained from the same biological sample as the data for the current study, and their out-of-sample counterparts obtained from independent biological replicates. Our results suggest that although all four types of reference data can be used to determine the optimal sequencing depth accurately, in-sample scRNA-seq and out-of-sample bulk RNA-Seq should be considered as the most and least preferable sources of reference data respectively.

In practice, there is enough experimental flexibility to choose both the total sequencing budget $B$ as well as the total number of cells ${n}_{{\mathrm{cells}}}$ to achieve the recommended allocation. The budget $B$ is typically specified in terms of the total number of lanes that will be used for sequencing and is directly proportional to the sequencing cost of the experiment. For example, the 10x Genomics’ pbmc_4k dataset was sequenced on one Illumina Hiseq4000 lane yielding a total of ~350 million reads, whereas the brain_1.3m data set was sequenced on 88 Hiseq4000 lanes (11 flow cells) yielding ~30 billion reads. Sample multiplexing can also be utilized to achieve fractional lane occupancies for smaller experiments. Now, given a fixed budget $B$, one can adjust the desired sequencing depth (${n}_{{\mathrm{reads}}}=B/{n}_{{\mathrm{cells}}}$) by selecting the total number of cells at the library preparation stage of the experiment. Although all single-cell RNA-seq assays rely on the Illumina platform for sequencing, the library preparation stage (e.g., single-cell isolation, mRNA capture, and barcoding) is technology-specific^22–24,28,43^. Nevertheless, it is possible to accurately choose the total number of cells by adjusting the cell concentration (cells/$\mu l$) and the final cell suspension volume that is going to be used in the process. For example, the 10x Genomics Chromium platform can be adjusted to yield from 500 to 10K cells per lane in a single run (10× user manual: https://support.10xgenomics.com/permalink/3vzDu3zQjY0o2AqkkkI4CC). For larger experiments, multiple lanes can be used (e.g., the brain_1.3m dataset was prepared on 133 10x Genomics Chromium lanes, each optimized to capture ~10k cells). Even though the library preparation stage can incur additional costs for a single-cell RNA-seq experiment, these costs are independent of the sequencing process, can vary significantly across different technologies^44^, and are in general decreasing rapidly.

### Empirical Bayes estimators

The EB estimators refer to the estimators that are aware of the noise model (which is Poisson here) and correct for the noise introduced by it. As they estimate the prior gene distribution ${P}_{{\bf{X}}}$ in the hierarchical model (2) from the observed data ${{\bf{Y}}}_{c}$, sometimes by estimating the moments of the prior distribution ${P}_{{\bf{X}}}$, they are usually associated with the names empirical Bayes, moment matching, or density deconvolution. Here, we use the term EB to refer to them in general.

As an illustrating example, consider a simplified model that for cell $c$ and gene $g$:

$${X}_{cg} \sim {P}_{{X}_{g}},\ {Y}_{cg}| {X}_{cg} \sim {\mathrm{Poi}}({X}_{cg}).$$

The plug-in estimator estimates the gene variance by the sample variance of UMIs, i.e.,

$${\widehat{var}}_{g}^{{\rm{plug-in}}}=\frac{1}{{n}_{{\rm{cells}}}-1}\sum _{c=1}^{{n}_{{\rm{cells}}}}{({Y}_{cg}-{\overline{Y}}_{cg})}^{2},$$

where ${\overline{Y}}_{cg}=\frac{1}{{n}_{{\mathrm{cells}}}}{\sum }_{c=1}^{{n}_{{\mathrm{cells}}}}{Y}_{cg}$ is the empirical mean. However, the estimated value is usually overly variable owing to the presence of the Poisson noise. Indeed,

$${\mathbb{E}}[{\widehat{var}}_{g}^{{\rm{plug-in}}}]={\rm{Var}}[{Y}_{cg}]={\rm{Var}}[{X}_{cg}]+{\mathbb{E}}[{X}_{cg}],$$

where the second term ${\mathbb{E}}[{X}_{cg}]$ corresponds to the technical variation introduced by the Poisson noise. Then, conceptually we can write:

$${\rm{plug}}{\hbox{-}}{\rm{in}}\ {\rm{variance}}={\rm{biological}}\ {\rm{truth}}+{\rm{Poisson}}\ {\rm{noise}},$$

from which we can see that the plug-in estimate is inflated by the Poisson noise. In this case, this bias can be easily corrected by simply subtracting the mean, and the corresponding EB variance estimator can be written as

$${\widehat{var}}_{g}^{{\rm{EB}}}=\frac{1}{{n}_{{\rm{cells}}}-1}\sum _{c=1}^{{n}_{{\rm{cells}}}}{({Y}_{cg}-{\overline{Y}}_{cg})}^{2}-\frac{1}{{n}_{{\rm{cells}}}}\sum _{c=1}^{{n}_{{\rm{cells}}}}{Y}_{cg}.$$

The EB estimators considered in the paper are listed in Table 1, along with the plug-in estimators for comparison. In literature, they are designed in a case-by-case fashion^16–18,24,45–47,47–50^ (more details in Supplementary Note 4).

**Table 1:** Comparison of the plug-in estimator and the EB estimator.

	plug-in	EB
1st moment ${M}_{1,g}$	$\frac{1}{{n}_{{\rm{cells}}}}{\sum }_{c=1}^{{n}_{{\rm{cells}}}}\frac{{Y}_{cg}}{{\gamma }_{c}{n}_{{\rm{reads}}}}$	same
2nd moment ${M}_{2,g}$	$\frac{1}{{n}_{{\rm{cells}}}}{\sum }_{c=1}^{{n}_{{\rm{cells}}}}\frac{{Y}_{cg}^{2}}{{({\gamma }_{c}{n}_{{\rm{reads}}})}^{2}}$	$\frac{1}{{n}_{{\rm{cells}}}}{\sum }_{c=1}^{{n}_{{\rm{cells}}}}\frac{{Y}_{cg}^{2}-{Y}_{cg}}{{({\gamma }_{c}{n}_{{\rm{reads}}})}^{2}}$
$k$th moment ${M}_{k,g}$	$\frac{1}{{n}_{{\rm{cells}}}}{\sum }_{c=1}^{{n}_{{\rm{cells}}}}\frac{{Y}_{cg}^{k}}{{({\gamma }_{c}{n}_{{\rm{reads}}})}^{k}}$	$\frac{1}{{n}_{{\rm{cells}}}}{\sum }_{c=1}^{{n}_{{\rm{cells}}}}\frac{{\prod }_{r=0}^{k-1}({Y}_{cg}-r)}{{({\gamma }_{c}{n}_{{\rm{reads}}})}^{k}}$
1st pairwise moment ${M}_{11,{g}_{1}{g}_{2}}$	$\frac{1}{{n}_{{\rm{cells}}}}{\sum }_{c=1}^{{n}_{{\rm{cells}}}}\frac{1}{{n}_{{\rm{reads}}}^{2}{\gamma }_{c}^{2}}{Y}_{c{g}_{1}}{Y}_{c{g}_{2}}$	same
Inactively probability ${p}_{0,g}(\kappa )$	$\frac{1}{{n}_{{\rm{cells}}}}{\sum }_{c=1}^{{n}_{{\rm{cells}}}}{{\mathbb{I}}}_{\{{Y}_{cg}=0\}}$	$\frac{1}{{n}_{{\rm{cells}}}}{\sum }_{c=1}^{{n}_{{\rm{cells}}}}{a}_{{Y}_{cg}}$
Pairwise inactive probability ${p}_{0,{g}_{1}{g}_{2}}(\kappa )$	$\frac{1}{{n}_{{\rm{cells}}}}{\sum }_{c=1}^{{n}_{{\rm{cells}}}}{{\mathbb{I}}}_{\{{Y}_{c{g}_{1}}\!={Y}_{c{g}_{2}}\!=0\}}$	$\frac{1}{{n}_{{\rm{cells}}}}{\sum }_{c=1}^{{n}_{{\rm{cells}}}}{a}_{{Y}_{c{g}_{1}}}{a}_{{Y}_{c{g}_{2}}}$
Distribution ${P}_{{X}_{g}}$	Empirical distribution of ${Y}_{cg}$ (scaled by $1/{n}_{{\rm{reads}}}$)	${\hat{P}}_{{X}_{g}}$ that most likely gives empirical distribution of ${Y}_{cg}$ via model (2)

The two estimators are written in similar forms for better comparison. For the inactive probability (and the pairwise case), ${a}_{{Y}_{cg}}$ is a coefficient that depends on ${Y}_{cg}$, $\kappa$, and ${n}_{{\rm{reads}}}$. See Inactive probability in Supplementary Note 5 for the exact expression and other details.

### Empirical evaluation of the tradeoff

We conducted two sets of simulations to evaluate the estimation error under different budget splits, which differ in how the data are generated. The first simulation (Fig. 2a) subsampled from a high-budget dataset consisting of the top 72 genes from the brain_1.3 m dataset. These genes were chosen because they have at least 10 reads per cell, providing a deep dataset to perform the subsample experiments. This simulation better matches the real data as the subsampling procedure does not assume the Poisson model (see Subsampling experiment in Supplementary Note 6). However, as we did not know the true gene distribution, the plug-in estimates of the high-budget dataset that we subsample from were used as proxies of the ground truth, against which we evaluated the estimation error. The second simulation, corresponding to Supplementary Fig. 4), generated the data according to model (2), where the true gene distribution ${P}_{{\bf{X}}}$ was obtained by using the empirical distribution of the first 100 highly expressed genes in the pbmc_4k dataset. This setting better validates the theory as it assumes the same model. Moreover, the estimation error is exact as the ground truth is available. Both simulations include many genes to address the heterogeneity of the gene distribution, and the genes considered here, being top genes in the dataset, have similar mean expression levels so that the mean reads over all genes can well represent the mean reads for each gene. Both simulations exhibit qualitatively the same behavior, validating the theory that the optimal depth (mean reads per cell per gene) is a constant that does not depend on the budget.

### Post hoc guidance for reliable estimation

The feasible region (top) and the post hoc table (bottom) were obtained via simulation, where we fixed the number of cells (1k, 5k, 10k, 30k, 70k) and studied how the error decreases as a function of the sequencing depth (Supplementary Figs. 5–6). The data were generated according to model (2) similar to the second tradeoff simulation, where the empirical distributions of the marker genes in pbmc_4k and brain_9k were used as the true gene distribution, respectively, to account for heterogeneity in different tissues. The true gene distribution was normalized so that each gene has the same mean expression level. As a result, the mean reads over all genes were the same as mean reads for each gene, providing a single-gene level error characterization. The post hoc table was obtained by finding the smallest sequencing depth such that the relative error was smaller than 0.1 (−2 in the log10 scale for the relative squared error and −1 for other errors, see Definition of errors in simulations in Supplementary Note 6). The results for both simulations were qualitatively the same. Hence, only the table for pbmc_4k was included.

### Comparing the performance of plug-in and EB estimators

Figure 3a demonstrates that the EB estimator is adaptive to different sequencing depths while the plug-in estimator is not. The top panel shows the estimated CV using the plug-in and EB estimators under different sequencing depths, where we can see clear inflation for the plug-in estimates. The full data are from pbmc_4k, and the subsample rate ranges from 0.2 to 1 (1 corresponds to the full data). The experiment was repeated five times, and the 3-std confidence interval was provided. The results for other genes, as well as for estimating the inactive probability, can be found in Supplementary Fig. 7. The middle panel compares the estimated CV from two datasets of the same tissue. Genes with at least 0.1 reads per cell were considered as our post hoc analysis showed that CVs of genes below this level could not be reliably estimated. The EB estimator may produce an invalid result when the plug-in variance is smaller than the plug-in mean of a gene, which was not accounted for by the Poisson model. Such cases were not common and were excluded while counting the number of genes that are above or below the red line. Hence, the total number of genes for the two panels may slightly differ. More results are in Supplementary Figs. 8–9. The bottom panel shows that the EB estimator can recover the gene distribution from shallow sequencing data. The shallow data were generated by subsampling to have 20% reads of the full data. For error evaluation, the recovered distribution was rescaled to have the same mean as the empirical distribution from the full data. See Supplementary Fig. 10 for more results.

Figure 3b investigates the common task where the most informative features (genes) were selected based on CV, and PCA was then performed on the selected features. The data were from pbmc_4k and was clipped at the 99th quantile to remove outliers. Such a procedure was also used in previous works on applying PCA to scRNA-seq data^11^. The top 500 genes with the highest CV were selected and the PCA scores were plotted for the 2nd and 3rd PC direction. The first direction was skipped because it corresponded to the variation in cell sizes. The results on the full data and the subsampled data (three times shallower) were compared, showing that the EB estimator is more consistent than the plug-in estimator.

Figure 4a considers recovering gene functional groups using Pearson correlation. We used the pbmc_4k dataset here as the biological structure of the PBMCs is well-understood. The major cell populations identified in this dataset are T cells (*IL7R*, *CD3D/E*, *LCK*), NK-cells (*NKG7*, *PRF1*, *KLRD1*, *GZMA*, *HOPX*, *CST7*), B cells (*CD79A*, *BANK1*, *IGHD*, *LINC00926*, *MS4A1*), myeloid-derived cells (*S100A8/9*, *MNDA*, *FGL2*, *CLEC7A*, *IFI30*) and megakaryocytes/platelets (*PF4*, *PPBP*). The heatmap of the EB-estimated Pearson correlation of those genes were visualized in Fig. 4a top, which shows that the EB estimator can well capture the gene functional groups. A subsample experiment was then conducted to investigate how well the estimators can recover the modules from the shallow sequencing data. The data were subsampled from the full data with rates 100% (full data), 25%, 10%, and 5%. The EB estimator can recover the module at a much shallower depth as compared to the plug-in estimator.

### Gene network analysis of the pbmc_4k dataset

The gene network (Fig. 4b) was constructed based on the EB-estimated Pearson correlation using the pbmc_4k dataset. The genes were filtered to have the EB-estimated variance larger than 0.1, resulting in 791 genes in total. A correlation larger than 0.8 was considered as a gene–gene edge. We found that varying the threshold from 0.4 to 0.95 did not significantly alter the result. The gene modules were identified based on knowledge of marker genes and gene pathways, as well as previous studies on PBMCs (see Gene module identification in Supplementary Note 6). We also note that the existence of megakaryocytes/platelets may be due to the imperfection of PBMC isolation, and since many genes were expressed in multiple cell populations (e.g., *CD74*, *CD27*), the resulting annotation only gives a rough picture of the underlying gene functional groups.

Next, we considered some important genes and plot their correlations with all other genes (Fig. 4c left, Supplementary Fig. 11). As a general phenomenon, the EB-estimated values are more spread out and exhibit different modes corresponding to genes that interact differently with the gene of interest. The plug-in estimated values are nonetheless much closer to zero even for genes that are known to be well-correlated.

Finally, we considered the gene pairs where the estimated values for the EB estimator and the plug-in estimator differ significantly (>0.7). Out of 1054 such pairs, 91 were also annotated based on STRING^51^, yielding a *p* value of 4.2e-11 while testing against the null hypothesis that the gene pairs were selected at random based on a one-sided hypergeometric distribution test (see Gene module identification in Supplementary Note 6). We plot the histograms of several such pairs and show that all of them have clear biological interpretations (Fig. 4c right, Supplementary Fig. 12). *LY86* (also known as *MD1*) is a secreted protein that has been shown to have an important role in T-cell activation, whereas *CD3E* is expressed within T cells (see Gene module identification in Supplementary Note 6). These two genes are not co-expressed and hence, are negatively correlated. *POMP* encodes a chaperone for proteasome assembly, whereas *PSMA7* is one of the 17 essential subunits for the complete assembly of the 20S proteasome complex. Hence, the two genes work together for proteasome assembly and should be positively correlated. The EB-estimated correlation is one and is probably an over-estimate owing to the randomness of the estimator. However, the actual Pearson correlation should not be much smaller than 1. In spite of the strong biological evidence, the plug-in estimator gives very small values owing to the presence of sequencing noise (See also Supplementary Fig. 12).

### smFISH experiments for validation

For validation, we considered two datasets, where both the scRNA-seq and the smFISH data are available. smFISH can be regarded as the gold standard for measuring the number of mRNAs in a cell and was used as a proxy for the ground truth (see Details of the smFISH experiments in Supplementary Note 6 for more details).

In the first dataset, both Drop-seq and smFISH were applied to the same melanoma cell line^15^. A total of 5763 cells and 12,241 genes were kept for analysis from the Drop-seq experiment, with a median of 1473 UMIs per cell. Of these genes, 24 were also profiled using smFISH. We further excluded genes with zero-UMI count in >97% of the cells and one more gene, *FOSL1*, owing to its abnormal behavior (*FOSL1* was also excluded in a recent work^16^ analyzing the dataset). We considered two distributional quantities, CV and inactive probability (with $\kappa =2.5{n}_{{\mathrm{reads}}}$), where we note that the latter has the interpretation of the proportion of zeros when the data were sequenced 2.5 times deeper. We compared the plug-in and the EB-estimated results from the Drop-seq data against the corresponding result from the smFISH data in Fig. 5a, where the smFISH estimates can be considered as the ground truth. Here, the gene *VCL* was omitted in the experiment of estimating the inactive probability because the corresponding smFISH data do not have enough cells to subsample from (4691, fewer than the number of cells captured by Drop-seq, which is 5763). The consistency between the EB estimates and the smFISH result indicates that the EB estimates are close to the ground truth. Furthermore, we investigated the optimal sequencing depth (Figure 5b and Supplementary Fig. 14) by fixing the budget and varying the sequencing depth, where the error was evaluated against the gold standard smFISH result. As this was done by subsampling from the original dataset, to ensure a wide range, only two genes with relatively more reads (*MITF* and *VGF*) were considered. Figure 5b and Supplementary Fig. 14 are qualitatively similar to the simulation results in Fig. 2a and Supplementary Fig. 4, showing an optimal depth between 0.1 and 0.6. This is consistent with our previous experiments based on 10x Genomics’ data and the experimental design guidelines we provide in this work, i.e., that the optimal depth for estimating different quantities is 0.2–1 read per cell per gene.

In the second dataset, both CEL-seq and smFISH were applied to the same mESC cell line and culture conditions^27^ (smFISH data from D. Grün, personal communication). Again, the plug-in and the EB-estimated results from the CEL-seq data were compared against the corresponding result from the smFISH data in Supplementary Fig. 13 for nine genes measured by smFISH, where we observed a good consistency between the EB and the smFISH results. As there are only 80 cells, we did not perform the subsampling experiment for this dataset.

Overall, the comparisons between the scRNA-seq and the smFISH results imply that our model matches the real data well, and the proposed EB estimator is able to provide estimates that are close to the ground truth. Also, the subsampling experiments in Fig. 5b and Supplementary Fig. 14 indicate that the optimal depth, evaluated using the smFISH data, is consistent with the main claim of the paper.

### Reporting summary

Further information on research design is available in the Nature Research Reporting Summary linked to this article.

## Supplementary information

Supplementary Information

Reporting Summary

## Data Availability

The 10× datasets were generated by 10x Genomics’ v2 chemistry^22^. They are publicly available and can be downloaded via the following links: pbmc_4k: https://support.10xgenomics.com/single-cell-gene-expression/datasets/2.1.0/pbmc4k pbmc_8k: https://support.10xgenomics.com/single-cell-gene-expression/datasets/2.1.0/pbmc8k brain_1k: https://support.10xgenomics.com/single-cell-gene-expression/datasets/2.1.0/neurons_900 brain_2k: https://support.10xgenomics.com/single-cell-gene-expression/datasets/2.1.0/neurons_2000 brain_9k: https://support.10xgenomics.com/single-cell-gene-expression/datasets/2.1.0/neuron_9k brain_1.3m: https://support.10xgenomics.com/single-cell-gene-expression/datasets/1.3.0/1M_neurons 293T_1k, 3T3_1k: https://support.10xgenomics.com/single-cell-gene-expression/datasets/2.1.0/hgmm_1k 293T_6k, 3T3_6k: https://support.10xgenomics.com/single-cell-gene-expression/datasets/2.1.0/hgmm_6k 293T_12k, 3T3_12k: https://support.10xgenomics.com/single-cell-gene-expression/datasets/2.1.0/hgmm_12k We note that pbmc_4k and pbmc_8k are from the same donor; brain_1k and brain_9k are also from the same donor. Also, the following pairs of datasets are sequenced together: 293T_1k and 3T3_1k, 293T_6k and 3T3_6k, 293T_12k and 3T3_12k. These six datasets are from the same biological sample. The Drop-seq dataset and the corresponding smFISH data can be found from the original paper^15^ or a recent paper that analyzed the dataset^16^. The CEL-seq data can be found from the original paper^27^. the smFISH data accompany the CEL-seq can be obtained by contacting the author. The three ERCC datasets (Zheng, Klein, Svensson) can be found in a recent paper that analyzed the data set^16^, where we have used the 2 × (control RNA + ERCC) data in the Svensson et al.^52^ paper. The Klein dataset with the pure RNA controls (the Klein ERCC dataset being part of it) can be found from the original paper^24^. The data for sensitivity analysis (Supplementary Figs. 18–19) can be found from the original paper^53^.

## Code availability

We developed the python package sceb (single-cell empirical Bayes) for the EB estimators used in this paper (available on PyPI). The code to reproduce all experiments and generate the figures presented in this paper can be found at https://github.com/martinjzhang/single_cell_eb.
