# Supplementary material for: Determining sequencing depth in a single-cell RNA-seq experiment
Source: Nat Commun. 2020 Feb 7;11:774. doi: 10.1038/s41467-020-14482-y (PMC7005864; doi:10.1038/s41467-020-14482-y)
Supplement: Supplementary file 1 — Supplementary Information [file 41467_2020_14482_MOESM1_ESM.pdf]

Determining sequencing depth in a single-cell RNA-seq experiment

Zhang et al.

Supplementary Information

4 Supplementary Figures

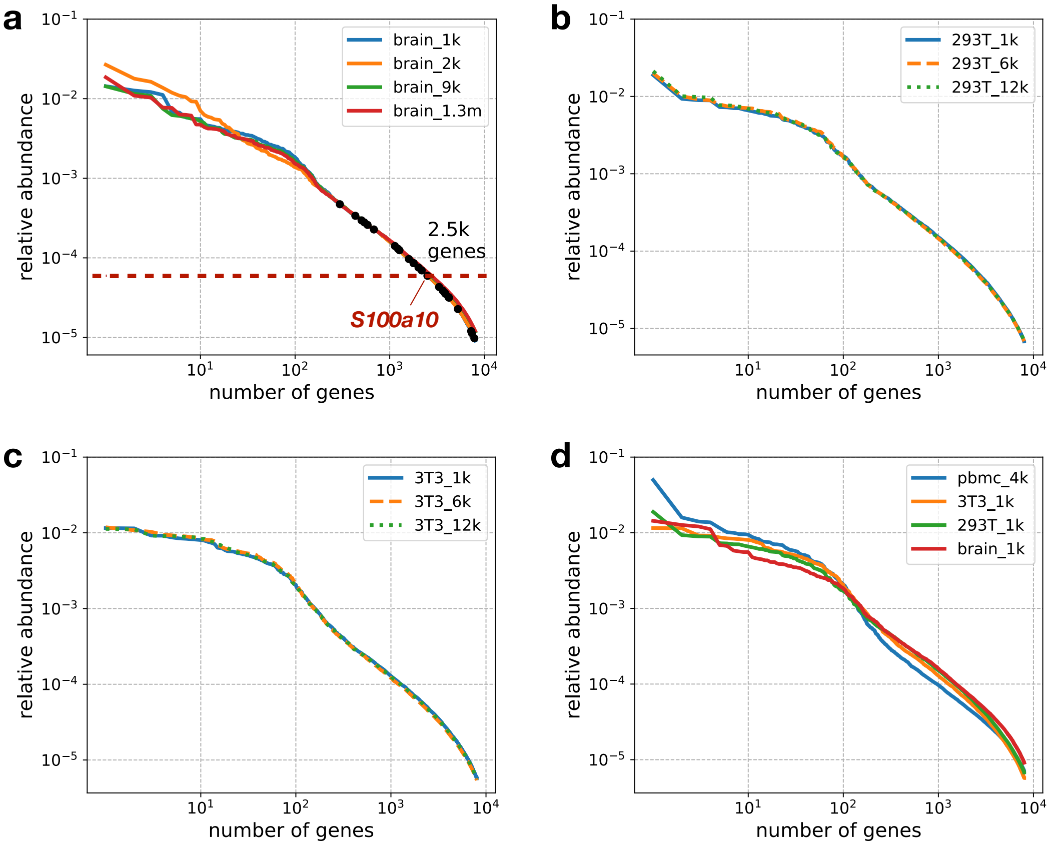

Supplementary Figure 1. Gene relative abundance curve. Panels a-c show the relative abundance curve for the brain datasets, 293T datasets and 3T3 datasets, respectively. These panels, along with the upper-left panel of Figure 1c, imply that the relative abundance of genes is consistent within the same tissue across different experiments. We note that brain_2k seems to be an outlier in panel a and in other experiments as well, e.g., Supplementary Figure 9c and Supplementary Figure 10d. Panel d compares different tissues and shows that the relative abundance curves are different for different tissues

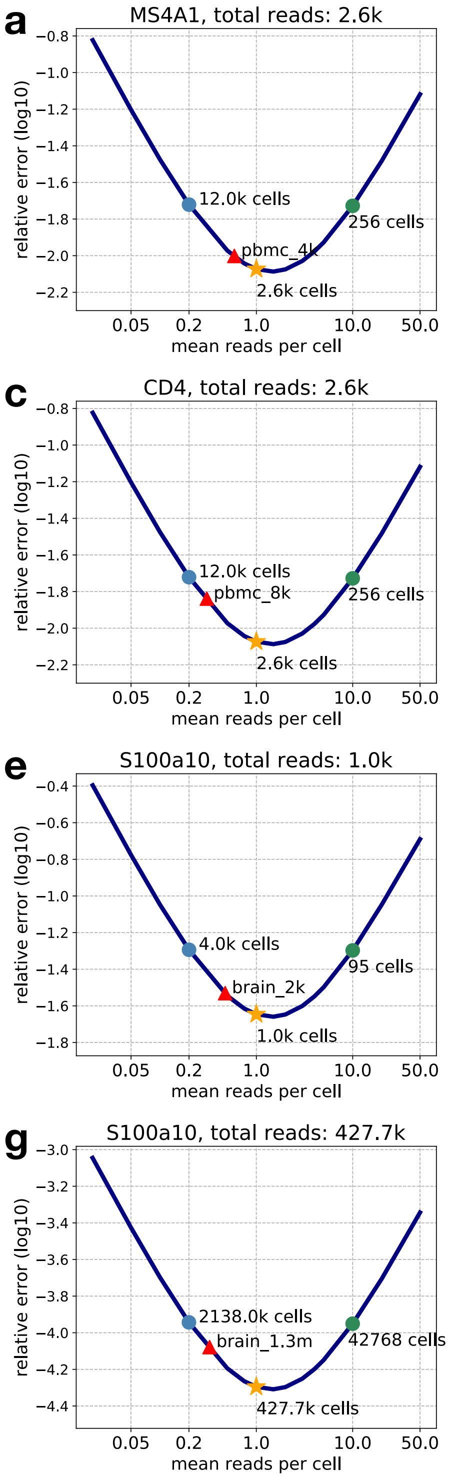

Supplementary Figure 2. Optimal trade-off. Panels a-b are two other genes (*MS4A1*, *CD4*) that we consider for pbmc_4k. Panel c is for pbmc_8 where we consider the gene *CD4* same as panel b. Panels d-g are for brain datasets (brain_1k, brain_2k, brain_9k, brain_1.3m) where we consider the gene *S100a10* (see Supplementary Figure 1a for the location of this gene on the relative abundance curve). All panels show that the current datasets should have been sequenced slightly deeper to achieve the optimal budget allocation

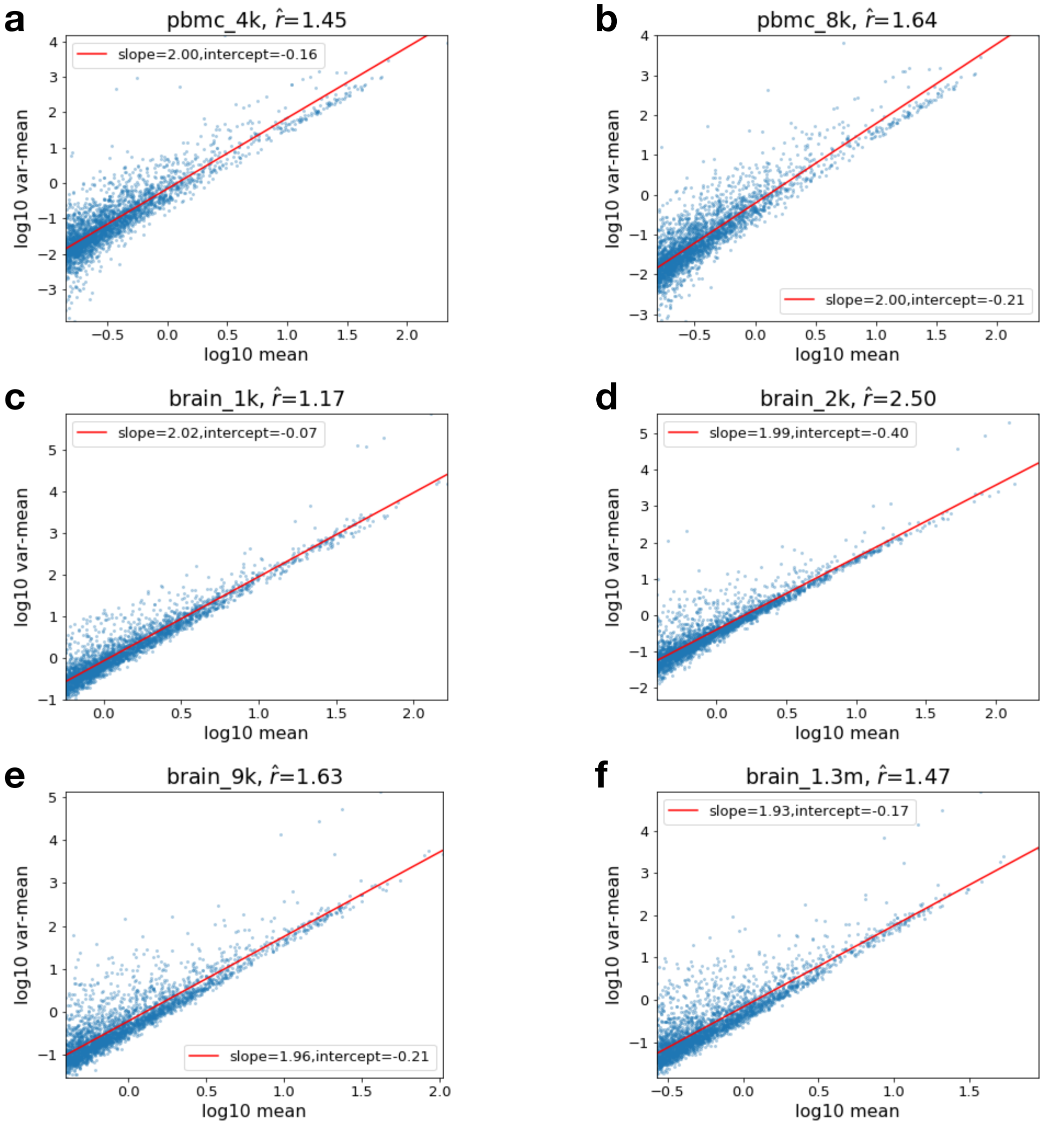

Supplementary Figure 3. Determine the shape parameter r for gamma distribution. Computing the theoretical error curve (Figure 1b, Supplementary Figure 2) requires knowing the shape parameter r for the gamma distribution, which is determined by the mean-variance relationship regression. Specifically, the overdispersion model assumes that $\mathrm{Var} = \mathrm{mean} + \frac{1}{r}\mathrm{mean}^2$. Then we can perform the log-scale regression $\log(\mathrm{Var} - \mathrm{mean}) = 2\log \mathrm{mean} - \log r$, where r corresponds to the negative log intercept. Panels a-f show the regression result on 6 different datasets, where the slopes are all very closed to 2, indicating that the mean-variance model is appropriate. The estimated values for the shape parameter r are between 1 and 2.5. Hence we choose $r = 1.5$ for computing all theoretical error curves

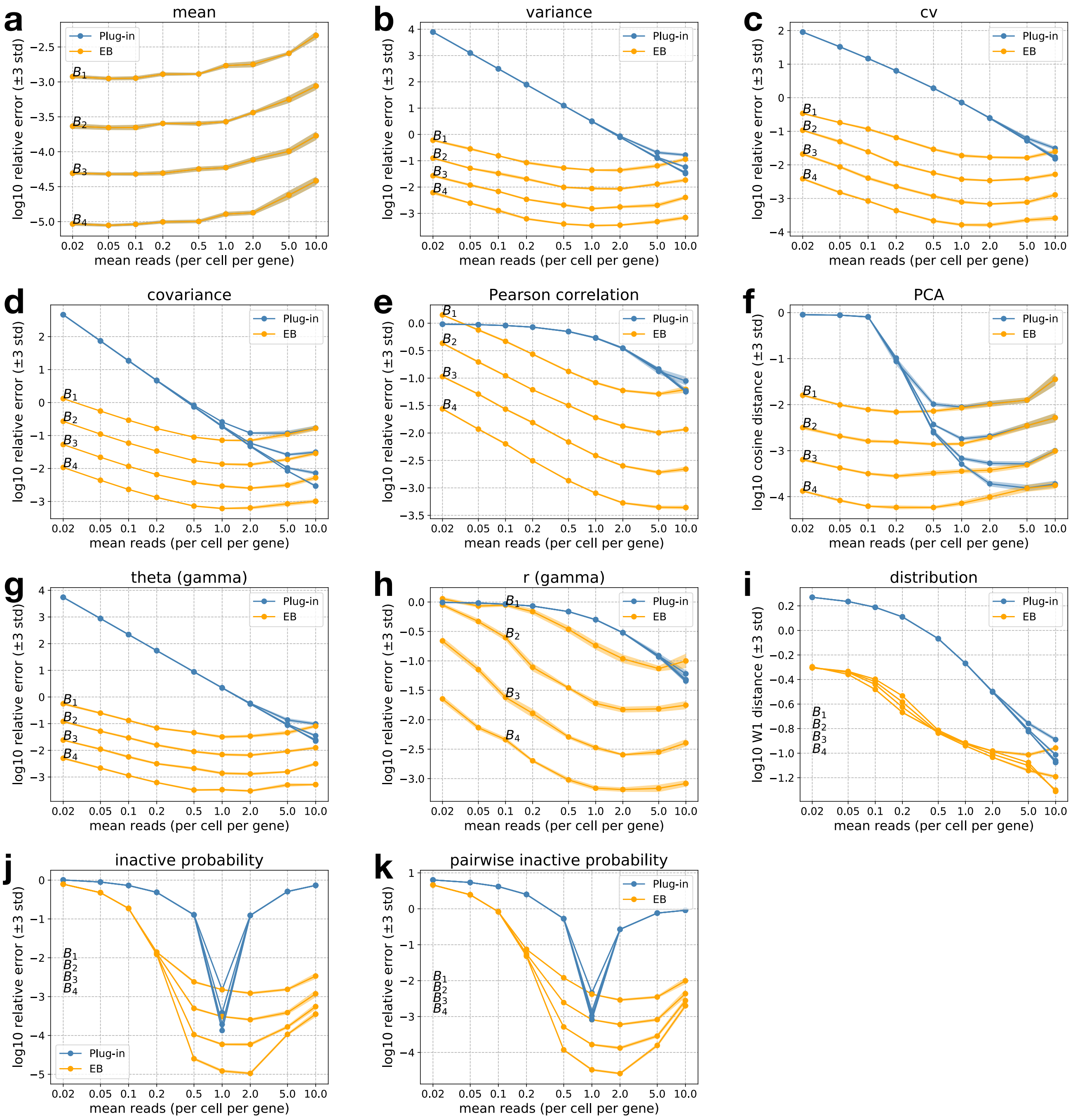

Supplementary Figure 4. Simulation of the trade-off curve. The simulation uses the top 100 highly-expressed genes in the pbmc_4k dataset. The budget is the same as Figure 2a except that we have added a fourth budget curve B_4 = 76k per gene. Without loss of generality, we choose κ to be the number of genes for defining the inactive probability as well as the pairwise inactive probability. All experiments are repeated 100 times and the 3-std confidence intervals are provided

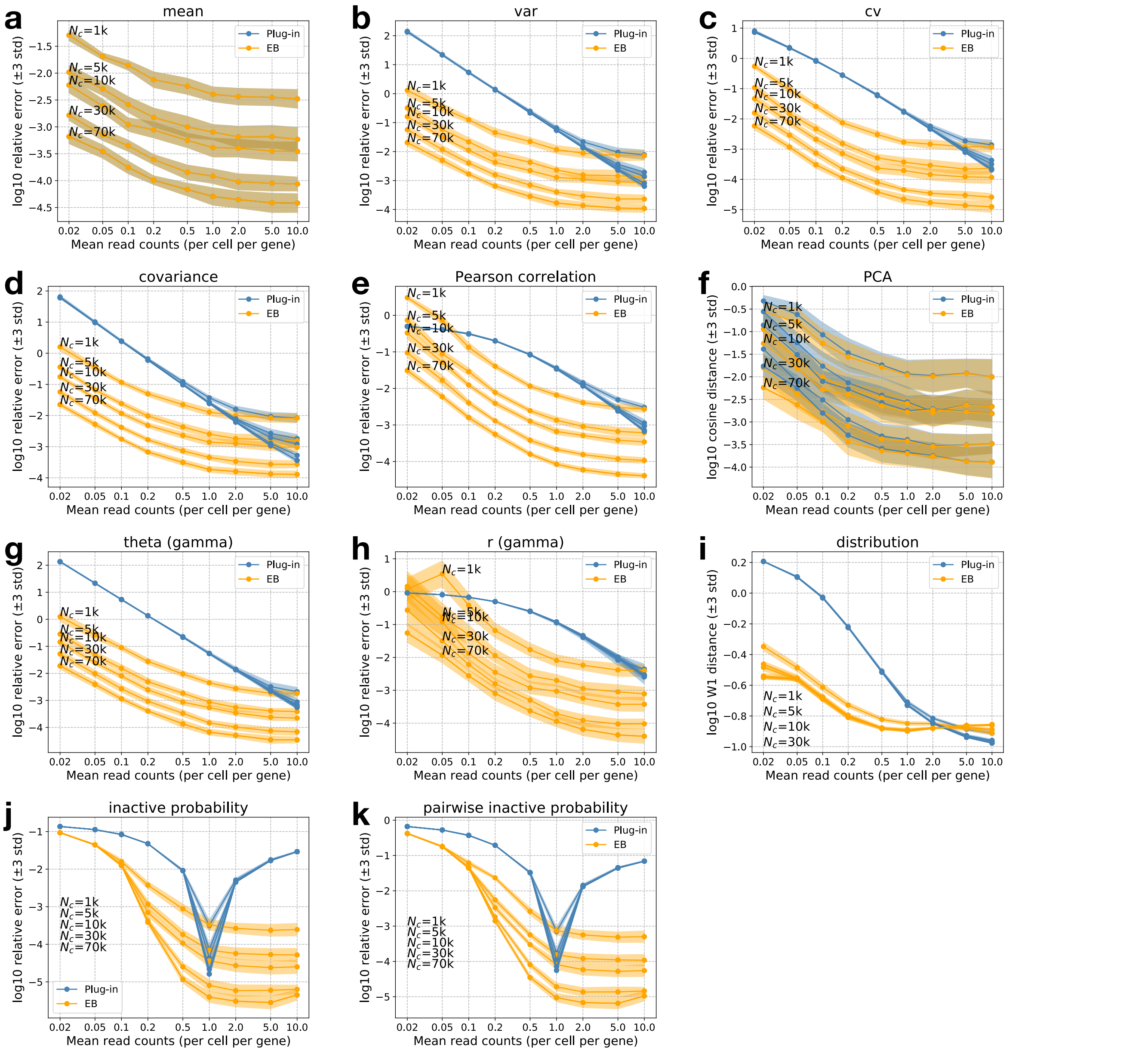

Supplementary Figure 5. Simulation for post-hoc analysis for PBMCs. We use the empirical distribution of marker genes in pbmc_4k as the true gene distribution, including *IL7R*, *CD3G*, *CD3E*, *CD3D*, *LCK*, *NKG7*, *GZMA*, *CST7*, *CD79A*, *MS4A1*, *S100A8*, *S100A9*, *MNDA*, *FGL2*. To provide a single-gene level error characterization, the gene distribution is normalized so that each gene has the same mean expression level. Then, the data is generated according to model (2). Without loss of generality, we choose κ to be the number of genes for the inactive probability as well as the pairwise inactive probability. All experiments are repeated 20 times and the 3std confidence intervals are provided. The simulation results are used to generate the post-hoc guidance table (Figure 2b), where the smallest mean reads (per cell per gene) such that the relative error is smaller than 10% is selected as the reliable detection threshold. We note that 10% corresponds to -2 for the log10 relative error (squared) and -1 for others (log10 W1, log10 cosine)

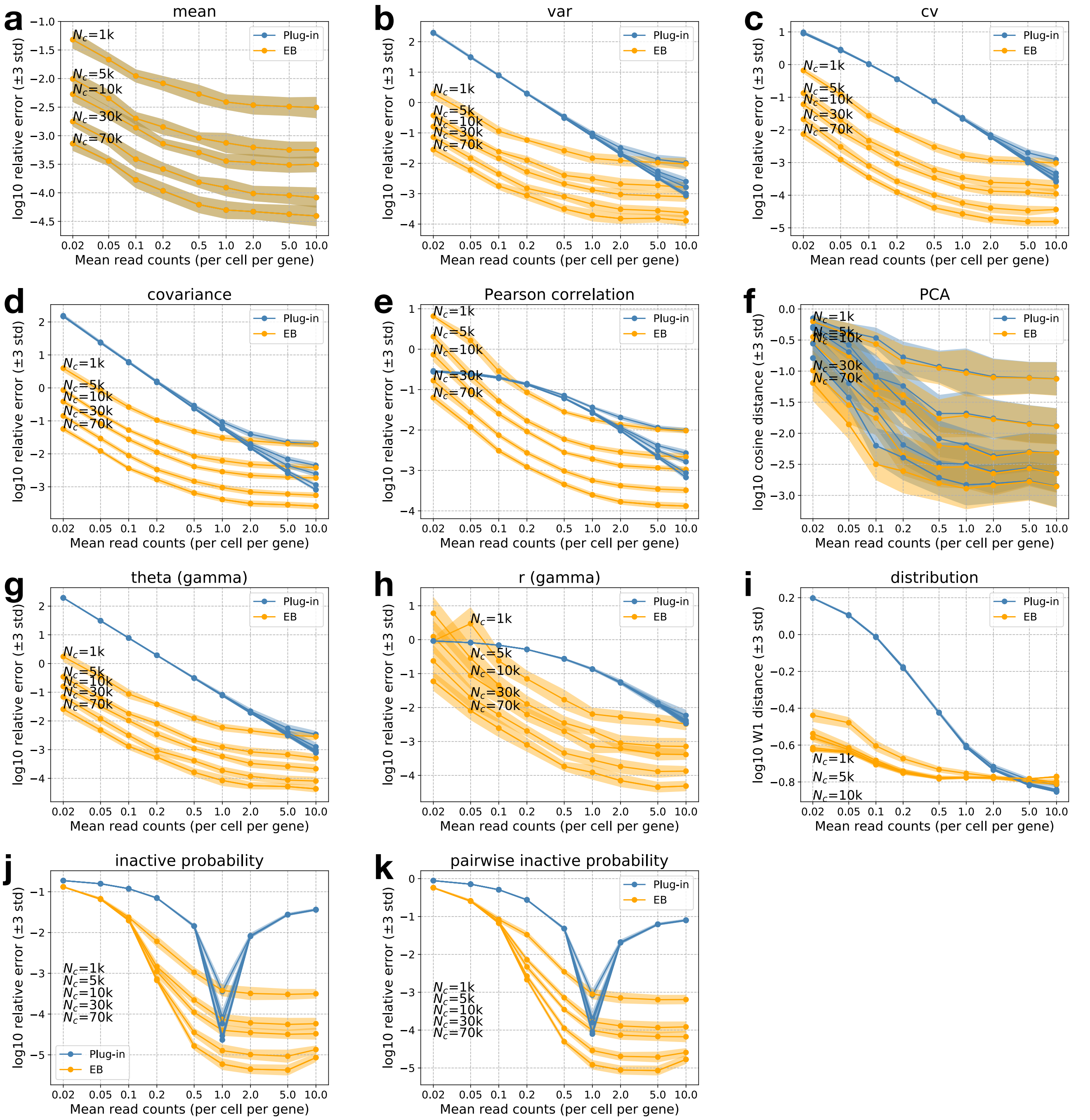

Supplementary Figure 6. Simulation for post-hoc analysis for mice brain. The same procedure as in Supplementary Figure 5 was used. We use the empirical distribution of marker genes in brain_9k as the true gene distribution, including *Gria2*, *Neurod1*, *Gadd45g*, *Clmp*, *Nrp1*, *Pcp4*, *Rnd2*, *Mef2c*, *Ptprd*, *Ly6h*, *Meg3*, *Tbr1*, *Pax6*. All procedures are the same as Supplementary Figure 5 and the results are also similar

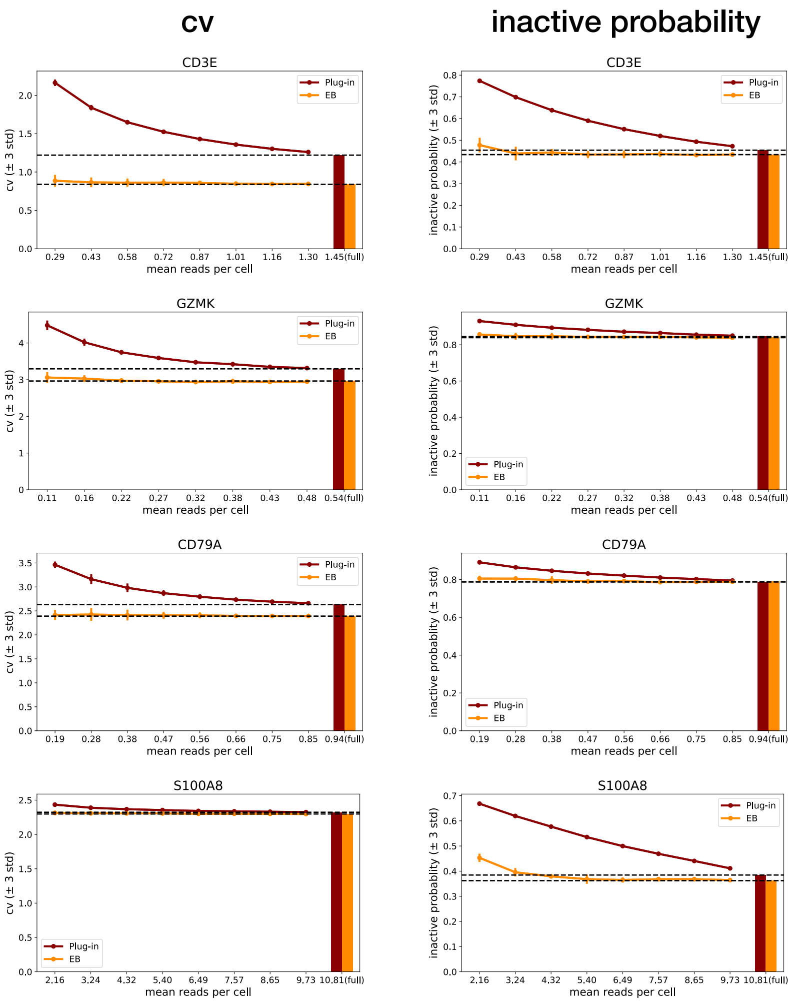

Supplementary Figure 7. Additional results to Figure 3a top. 3-std confidence intervals are provided. *CD3E*, *GZMK*, *CD79A*, and *S100A8* are marker genes for the T-cells, NK-cells, B-cells, myeloid-derived cells respectively. For the inactive probability, we choose κ to be equal to the sequence depth (n_{reads}) of the full data. As a result, the inactive probability corresponds to the proportion of zeros of the full data, where, as a sanity check, we can see that the EB estimates and the plug-in estimates are very similar at the depth of the full data (right most)

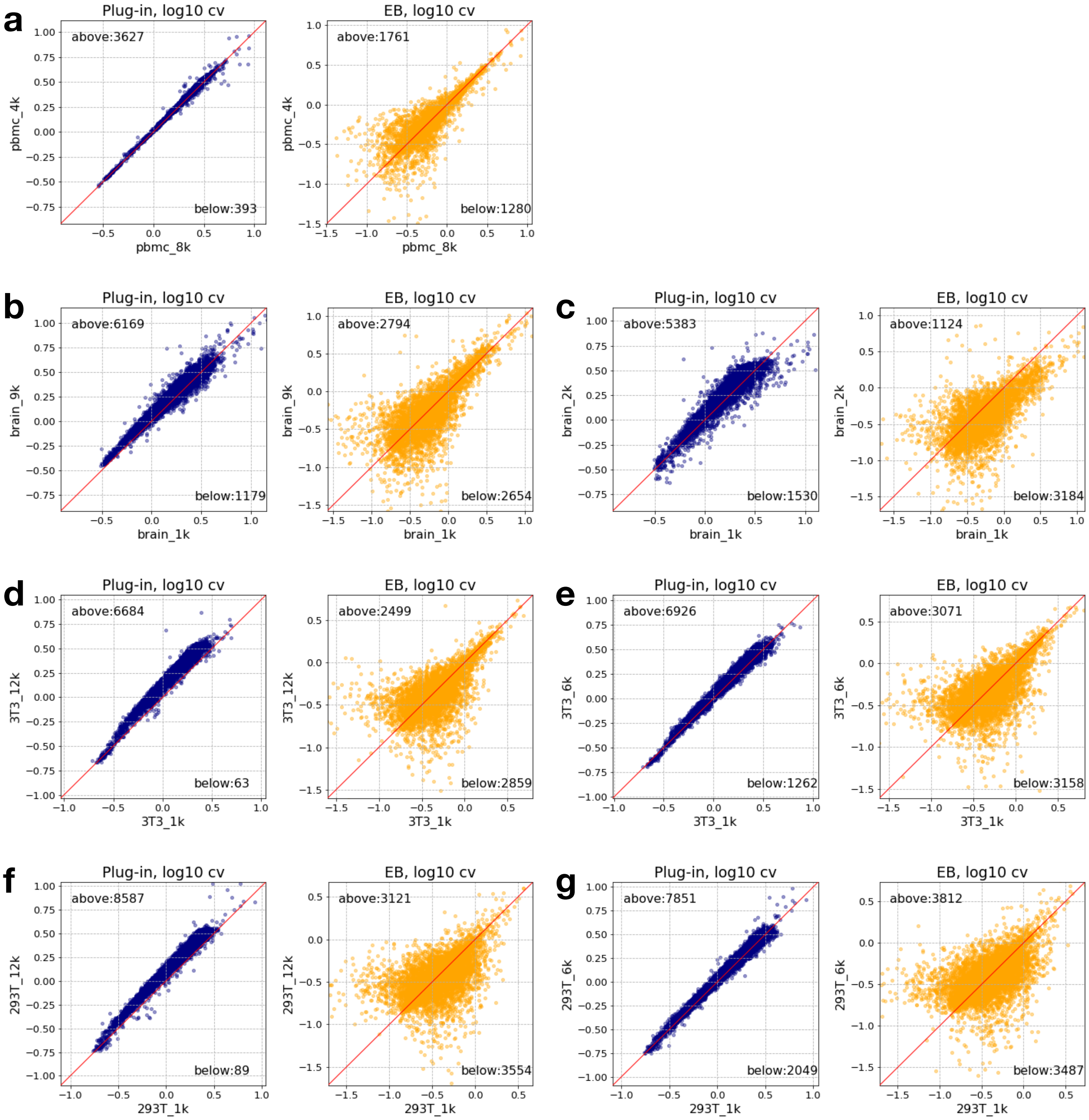

Supplementary Figure 8. Consistency plot for cv. Genes with mean read counts smaller than 0.1 are discarded prior to the experiment. We note that the variation of EB estimates are higher when the estimated values are small. This is because the plug-in estimator introduces an artificial bias that reduces the variance. In other words, despite the high variation, the EB estimates are still preferred since they are closer to the biological truth. See Variation of the EB estimates in Supplementary Note 4 for more details

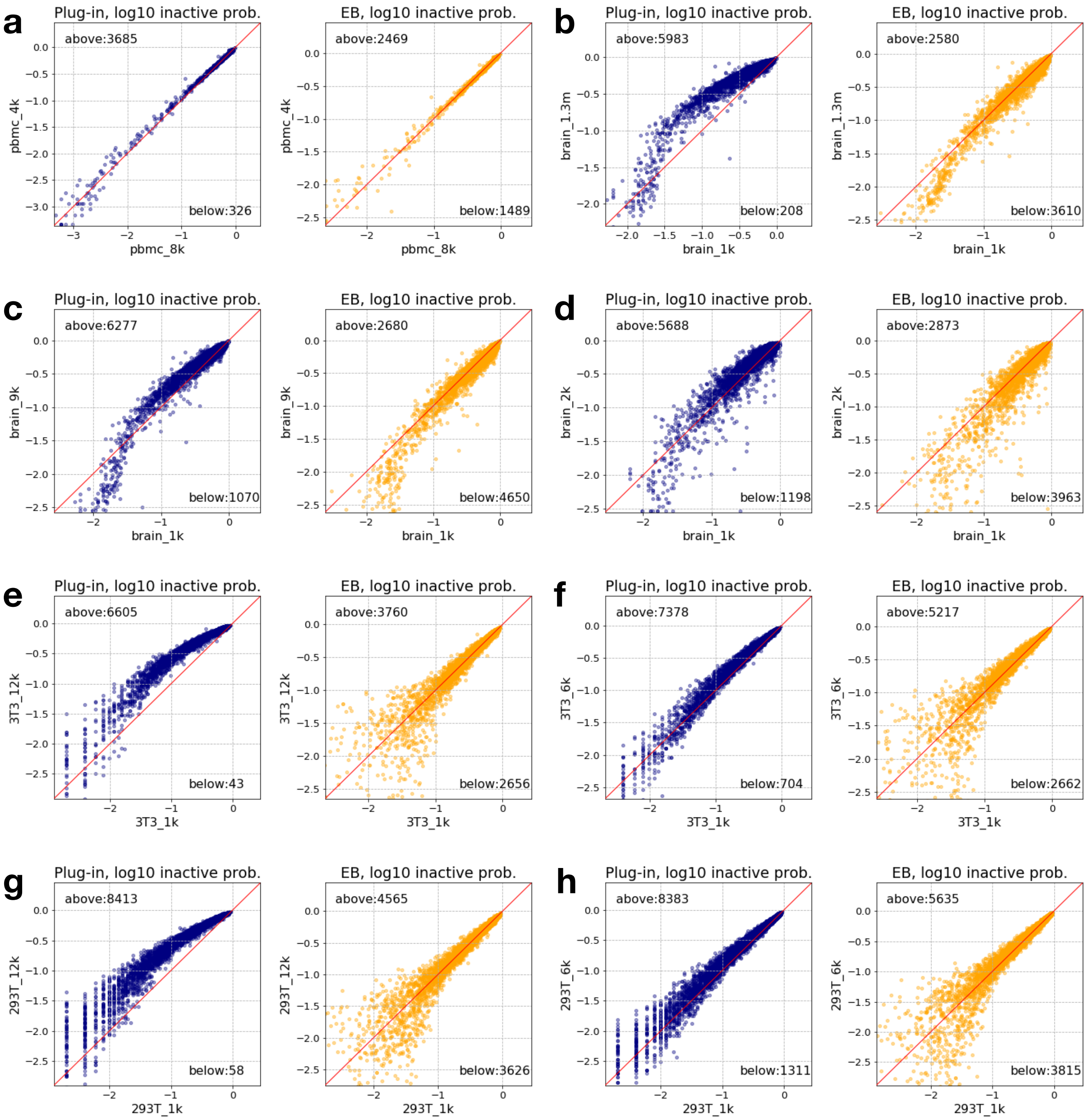

Supplementary Figure 9. Consistency plot for inactive probability. The inactive probability parameter κ is chosen to be the sequencing depth (n_{reads}) of pbmc_4k, brain_9k, 3T3_6k, 293T_6k for the corresponding tissues respectively

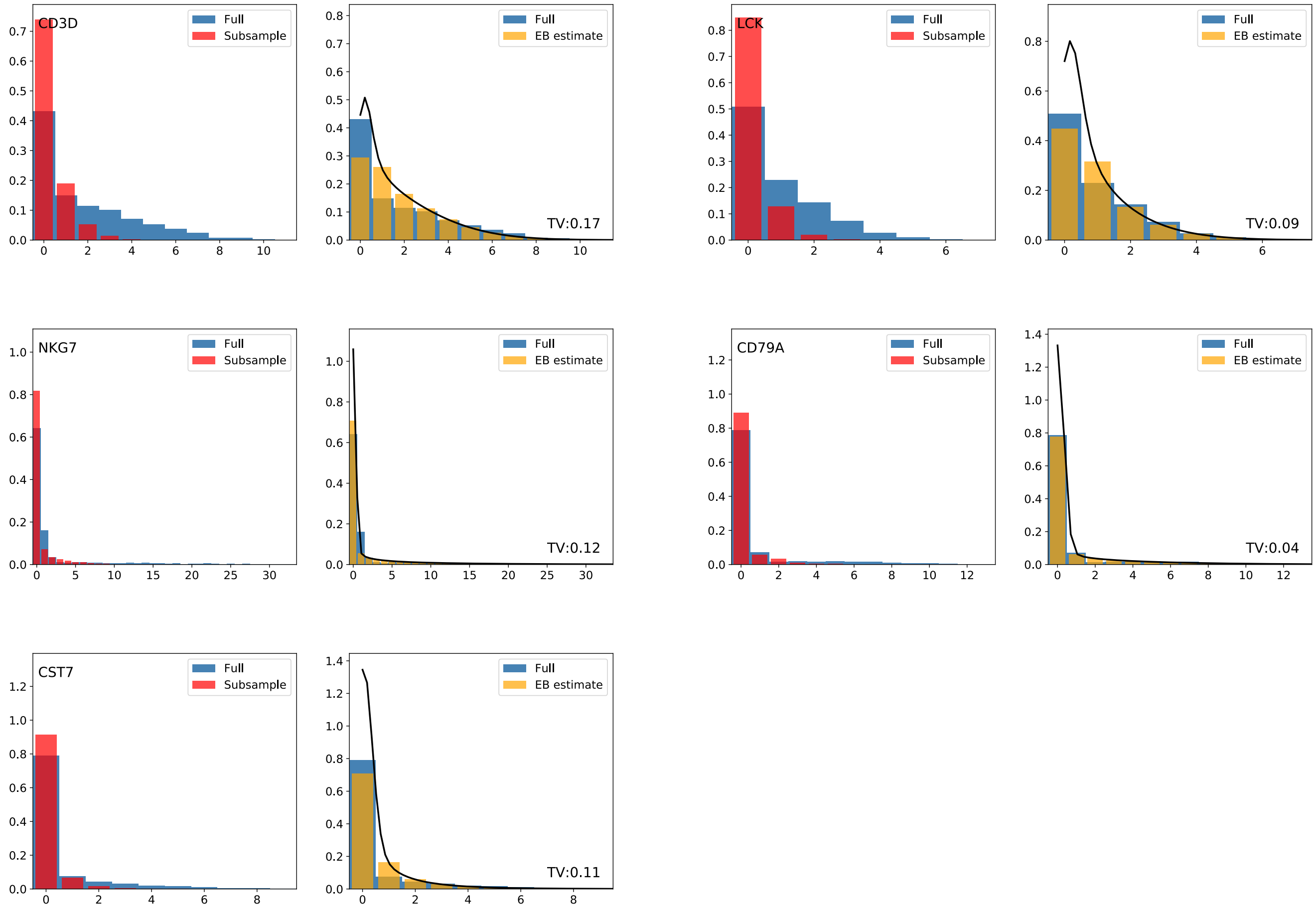

Supplementary Figure 10. Additional results to Figure 3a bottom. To compute the W1 distance, we match the two distributions to have the same mean, and then compute the W1 distance according to the standard definition. We also match the mean of the EB distribution and the original distribution for visualization purpose

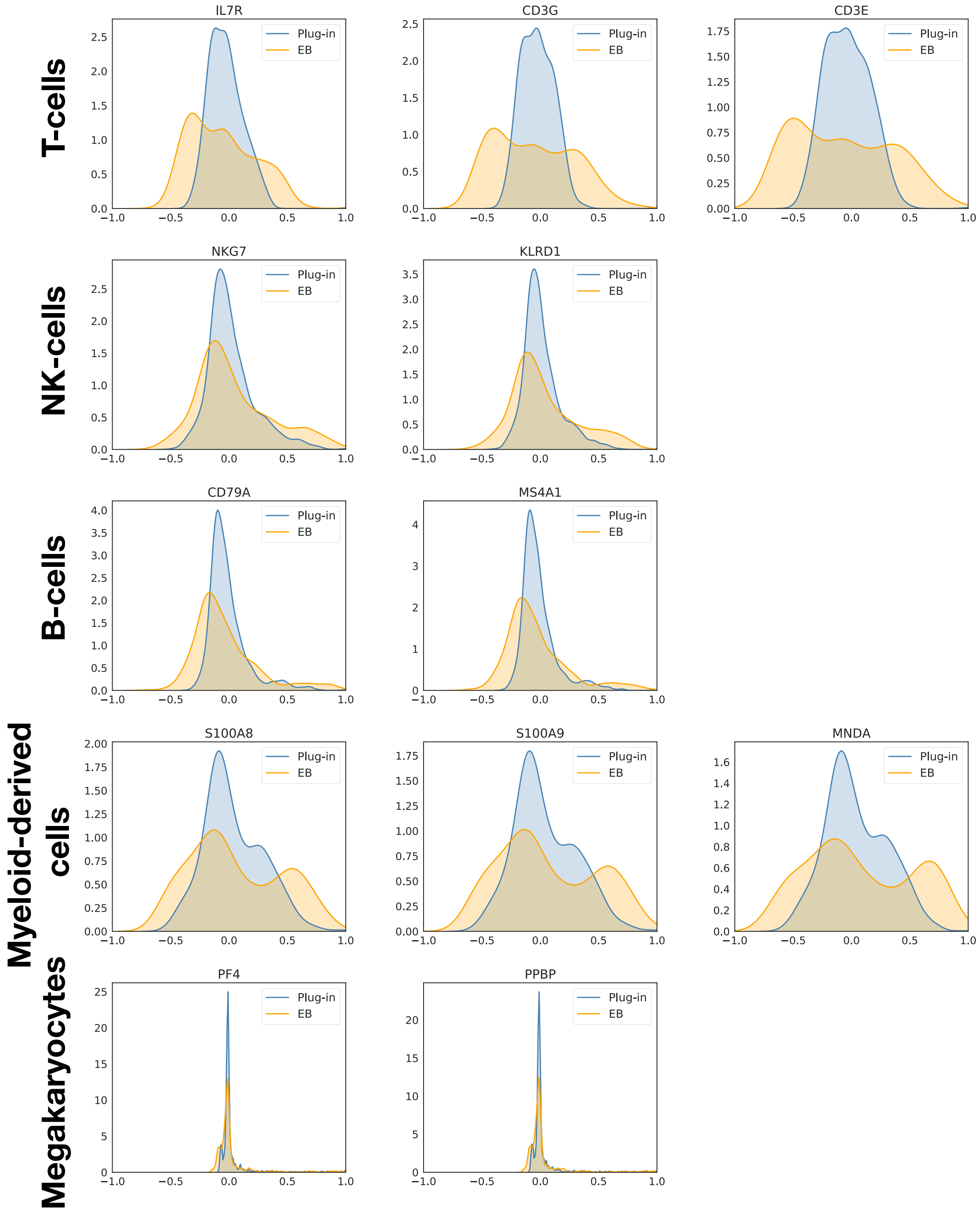

Supplementary Figure 11. Estimated correlations of some important genes with all other genes. The histograms are smoothed by Gaussian kernel density estimation

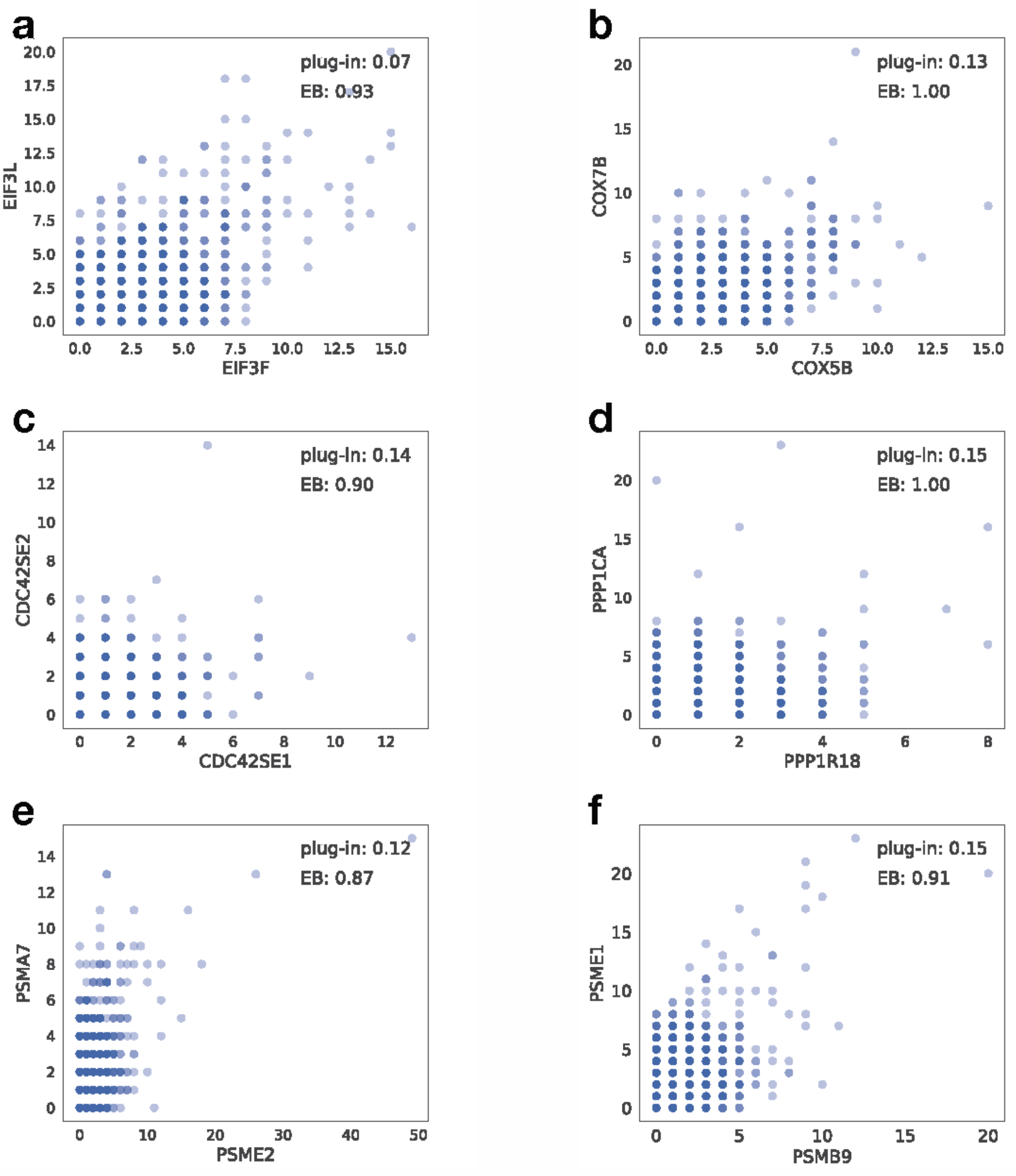

Supplementary Figure 12. Some cases where the estimated correlations for EB and plug-in differ significantly. In most cases the biological relation for the gene pairs can be inferred directly from the corresponding gene names. a) *EIF3F* and *EIF3L* encode the F and L subunits of the Eukaryotic Translation Initiation Factor 3. b) *COX7B* and *COX5B* encode the 7B and 5B subunits of Cytochrome C Oxidase. c) *CDC42SE1* and *CDC42SE2* are paralogous genes encoding *CDC42* small effector proteins 1 and 2. d) *PPP1CA* and *PPP1R18* encode the subunits of Protein Phosphatase 1. e-f) *PSMA7*, *PSME1*, *PSME2*, and *PSMB9* encode the subunits of the proteasome

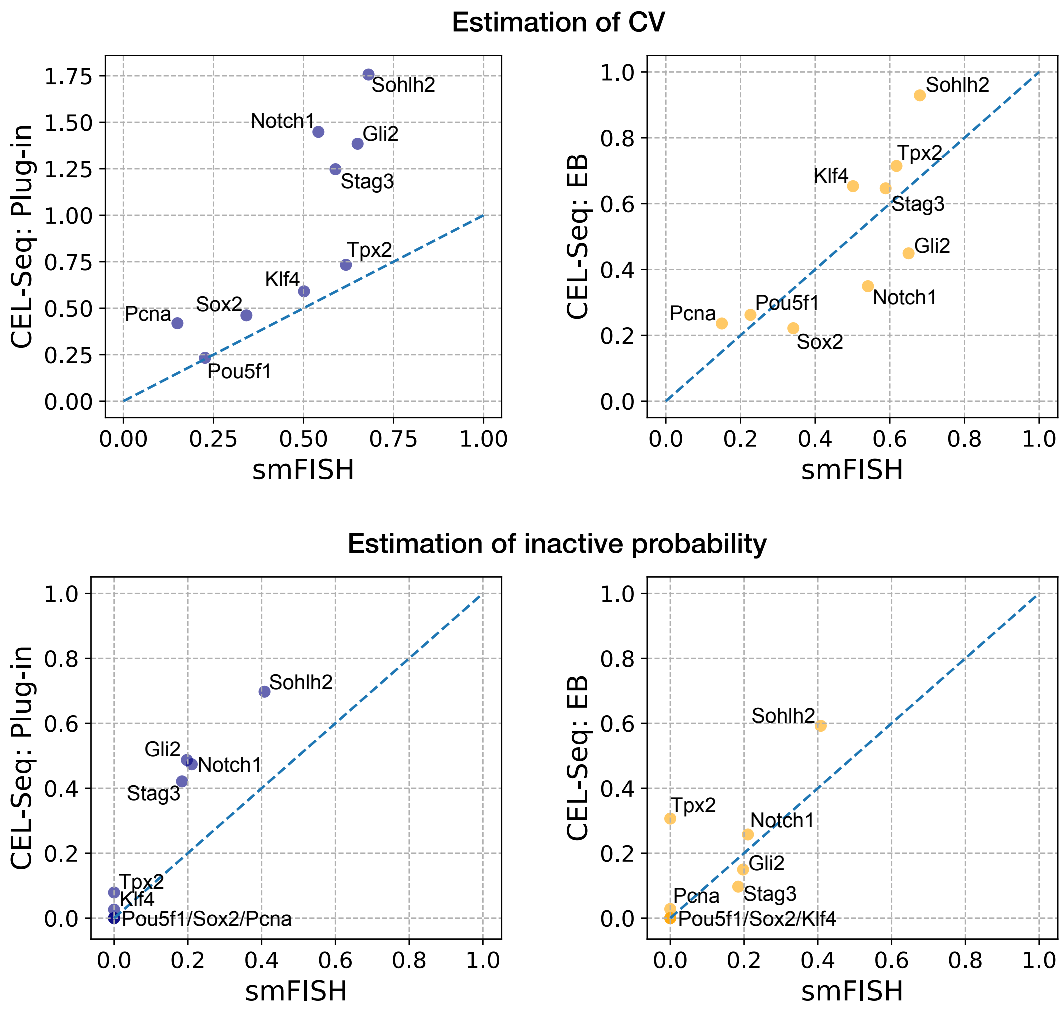

Supplementary Figure 13. The estimated cv (top) and inactive probability (bottom, $\kappa = 2.5n_{\text{reads}}$) from the CEL-seq data are compared with the smFISH results

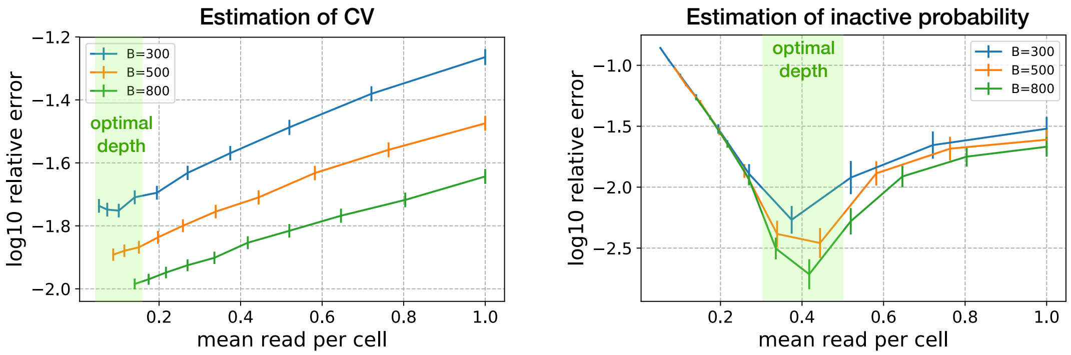

Supplementary Figure 14. The sequencing budget trade-off for estimating cv (left) and inactive probability (right, $\kappa = 1$, i.e., estimating the zero proportion at one read per cell) for the gene *VGF*. The relative error is evaluated against the gold standard smFISH result. 3std confidence intervals are provided

Estimates of various quantities for ERCC datasets

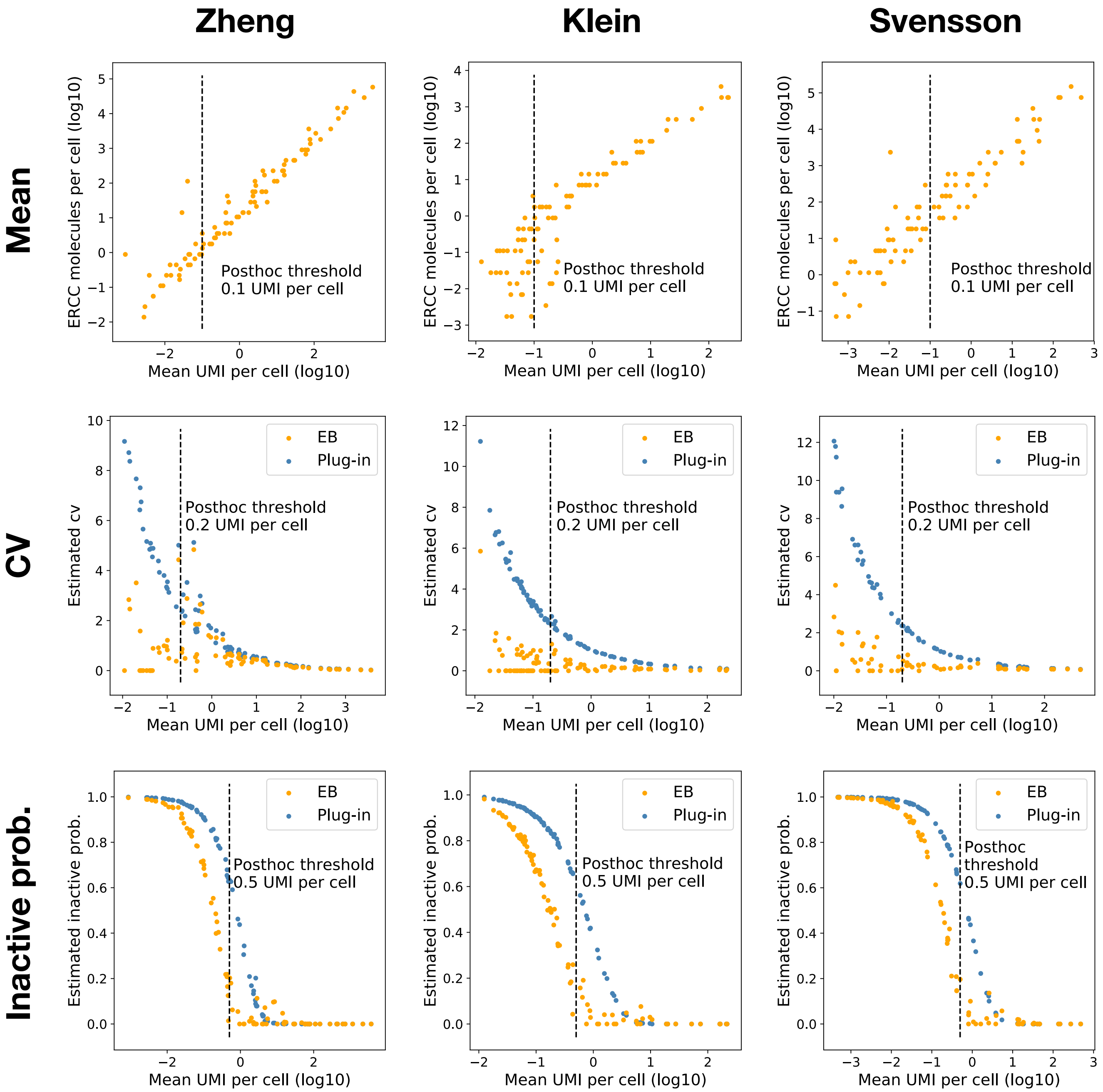

Supplementary Figure 15. Estimated mean, cv, and inactive probability ($\kappa = 5n_{\text{reads}}$) for three ERCC datasets. For ERCCs, the expected number of molecules can be estimated from the known ERCC concentration and the dilution factor. Both cv and inactive probability are expected to be close to zero since the number of ERCC molecules should be the same for all cells. The post-hoc thresholds (Figure 2b, Details of the ERCC experiments in Supplementary Note 6) are shown with a black dashed line. As we can see, beyond this threshold the EB estimates are very close to the ground truth. In contrast, the the plug-in estimates are inflated due to the technical noise and become close to the EB estimates only for the ERCCs with more UMIs

Pure RNA controls (human lymphoblastoma, K562)

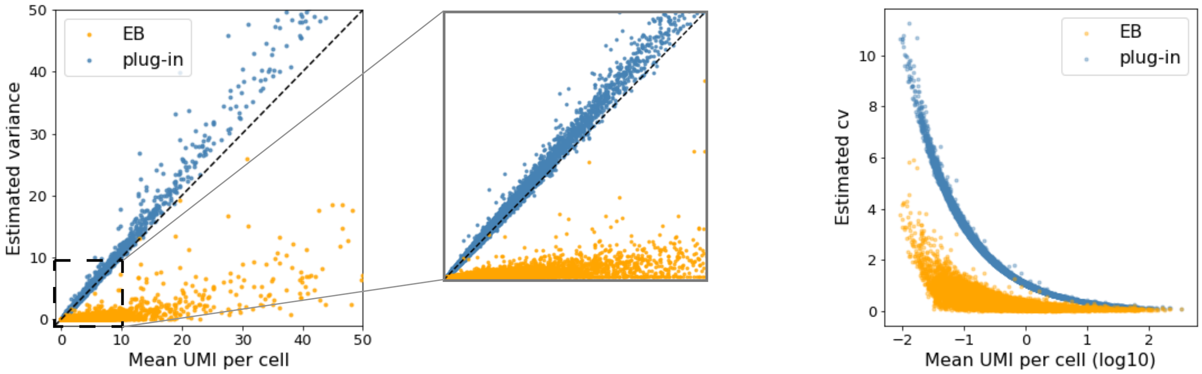

Supplementary Figure 16. The Klein dataset contains pure RNA controls that are also expected to display very small biological variability across cells. The estimated variance and cv for all genes are shown in the panel, where the EB estimates are very close to zero while the plug-in estimates are inflated due to the technical variation

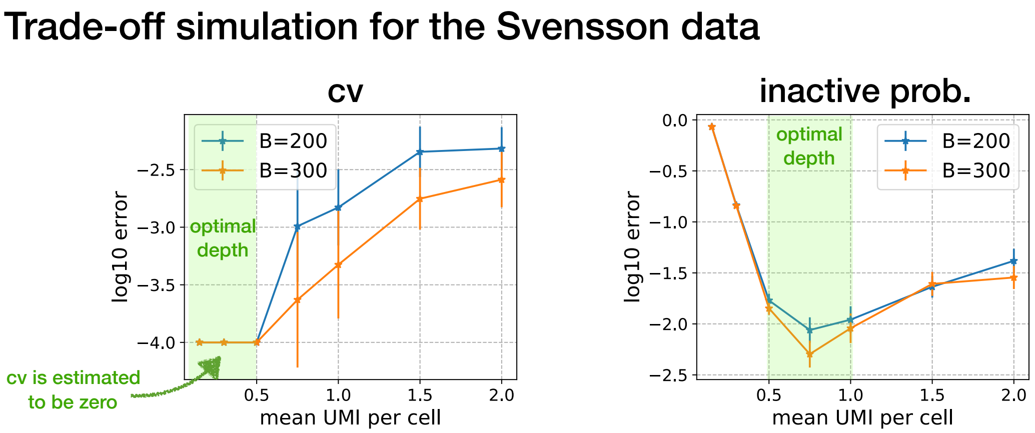

Supplementary Figure 17. The sequencing budget tradeoff for estimating cv (left) and inactive probability (right), where the optimal depth is below 0.5 for estimating cv and 0.5-1 for estimating the inactive probability. We note that the flat area on the left panel is because the estimated cv is 0 and is truncated to have a finite y-axis value. The details of the simulation is as follows. The tradeoff curve is averaged over 6 ERCC spike-ins in the Svensson data that have enough UMIs to subsample from and have similar depths (average UMIs between 10-20). The error is averaged over the 6 ERCCs and is evaluated against the ground truth, i.e., 0 for both cv and inactive probability. For example, the error for cv is $\log 10(\text{mean}(\hat{\text{cv}}^2))$, where ground truth is zero and the mean is over the 6 ERCCs. The inactive probability is estimated using $\kappa = 5n_{\text{reads}}$. All points are simulated with 100 repetitions and 3std confidence intervals are provided

	Reference data subsampled to have 500 cells and 2000 UMI per cell			
Reference type	In-sample scRNA-seq	Out-of-sample scRNA-seq	In-sample RNA-Seq	Out-of-sample RNA-seq
Reference data	10x2B_pbmc1	pbmc_4k	bulk_pbmc1	bulk_pbmc2
Data for the current study	10x2A_pbmc1			

Supplementary Figure 18. To assess the sensitivity of using reference data to estimate the detection limit p^* for the proposed experimental design procedure, we consider four different types of reference data based on the biological sample and the sequencing technology (first row). Here, "in-sample" means that the reference data is from the same biological sample as the data for the current study. E.g., the reference data may come from the pilot experiment. Similarly, "out-of-sample" means that the reference data is from a different biological sample, e.g., obtained from independent replicate or some other publicly available dataset. To simulate these four scenarios, we consider four additional PBMC datasets from a recent study[1] (rows 2-3). Specifically, 10x2A_pbmc1, 10x2B_pbmc1 are two scRNA-seq datasets generated with the 10x v2 technology which is the same as the other 10x datasets considered in the paper. bulk_pbmc1 and bulk_pbmc2 are two bulk RNA-Seq datasets. The first three datasets, i.e., 10x2A_pbmc1, 10x2B_pbmc1, and bulk_pbmc1, are from the same biological sample. These four datasets, along with the pbmc_4k dataset considered in the paper before, cover all four reference data types considered in this simulation. In addition, to provide a fair comparison and to match the smaller scale of a pilot experiment, we subsample the two scRNA-seq reference data, i.e., 10x2B_pbmc1 and pbmc_4k, to have 500 cells and 2000 reads per cell each.

The results are shown in Supplementary Figure 19 for the four reference data types respectively. As shown on the left panels, there is a good concordance of the relative abundance between the reference data and the data for the current study for each gene. Furthermore, on the right panels, we consider each gene as the gene of interest at the detection limit p^* separately and assume the true value of p^* is given by the value from the data for the current study. Using this value of p^* in the proposed experimental design procedure, the corresponding ideal estimation error is computed using the closed-form formula (Figure 1b). Not knowing p^* in reality before the experiment, we estimate it using the reference data and use the estimated value for the proposed experimental design procedure (Figure 1c) to determine the sequencing depth, resulting in a reference-based error which is greater than the ideal error. We show the ratio between the reference-based error and the ideal error on the right panels. As we can see, the ratio is small for most genes above the rare gene boundary (7 transcripts per cell for a cell with 300k transcripts in total, see Experimental design in the Methods section). Specifically, of all the genes above the rare gene boundary, 99%, 95%, 92%, 90% have an error ratio <1.5 for the four reference data types, respectively. We hence conclude that all four types of reference data can be used to accurately determine the optimal sequencing depth. We also note that in terms of preference for the reference data, in-sample scRNA-seq is most preferable while out-of-sample RNA-Seq is least preferable.

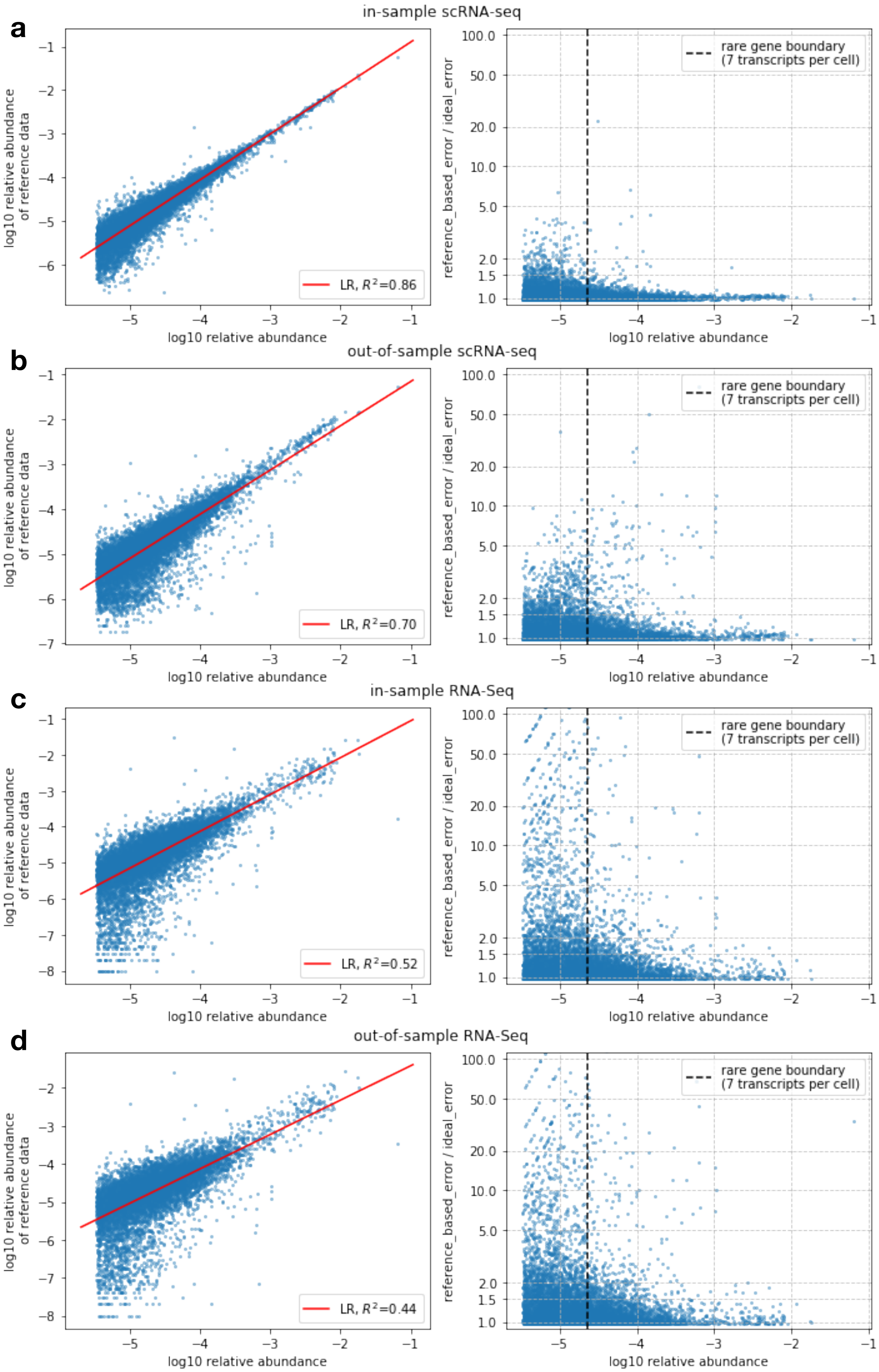

Supplementary Figure 19. See caption in Supplementary Figure 18

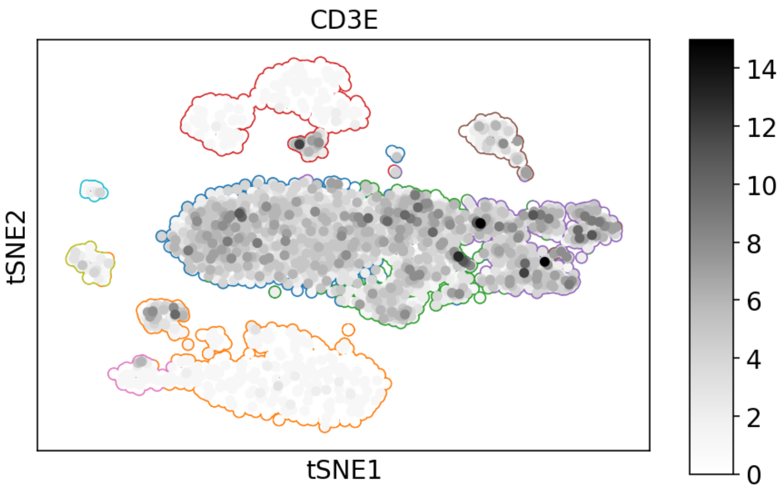

Supplementary Figure 20. The expression level of *CD3E* in the pbmc_8k dataset. The point clouds correspond to cells and a darker color means more highly expressed. The data is processed using the default setting in scanpy[2] (the recipe in the original paper[3])

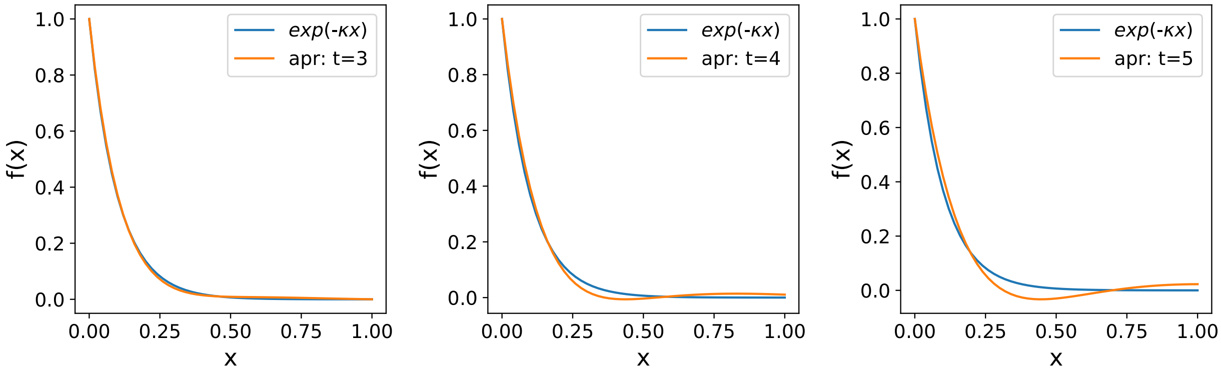

Supplementary Figure 21. The bias for the EB inactive probability estimator with different t_c (t in the figure label)

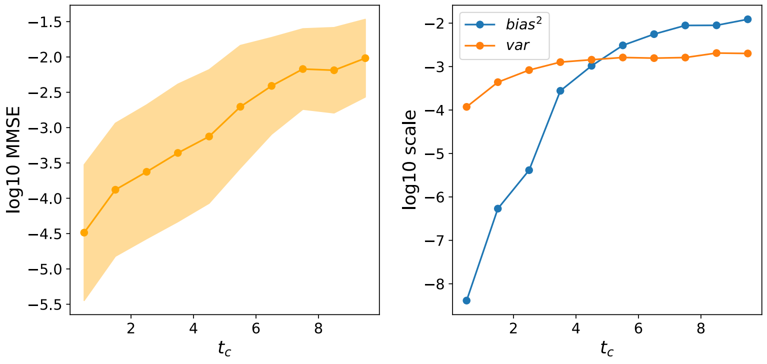

Supplementary Figure 22. The error of the EB inactive probability estimator on a toy example

Supplementary Note 1 Problem Formulation

Consider a single-cell RNA-seq (scRNA-seq) experiment where we sequence n_{cells} cells with n_{reads} reads per cell on average, where we note throughout the paper by read counts we mean the unique molecular identifier counts (UMIs) and by sequencing depth we mean the total number of UMIs per cell. The total number of reads can be written as $B = n_{\text{cells}} \times n_{\text{reads}}$ and can be thought of as the budget we have for this experiment. This budget is directly related to the resource we can spend on this particular experiment—in many scientific studies, there is usually a constraint on this budget. Fixing the budget B, there are different ways to split it between n_{cells} and n_{reads}: we can sequence many cells with few reads per cell or a few cells with mean reads per cell. Given some common statistical inference tasks, we are interested in the optimal allocation of the budget B between n_{cells} and n_{reads} that achieves the smallest estimation error, which we call the sequencing budget allocation problem.

1.1 The Poisson sampling model

The data can be summarized by a $n_{\text{cells}} \times G$ cell-gene matrix, where G is the number of genes and the c-th row, $\mathbf{Y}_c \in \mathbb{Z}^G$, represents the observed read counts for cell c, where $c = 1, \cdots, n_{\text{cells}}$. We assume that the data is generated via the following statistical model.

For cell c, let its relative gene expression level be represented as $\mathbf{X}_c = [X_{c1}, \cdots, X_{cG}] \in \Delta^G$, where Δ^G represents the probability simplex which imposes the constraint that $\sum_{g=1}^{G} X_{cg} = 1$. We note that $\mathbf{X}_c$ is only associated with the biology and is independent of the sequencing process. Without loss of generality we assume that $\mathbf{X}_c$'s are samples drawn independently and identically distributed (i.i.d.) from some unknown cell distribution $P_{\mathbf{X}}$, i.e.,

$$\mathbf{X}_c \overset{\text{i.i.d.}}{\sim} P_{\mathbf{X}}, \ \forall\, i \in [n_{\text{cells}}], \tag{4}$$

where we use the standard notation $[n] = \{1, 2, \cdots, n\}$. $P_{\mathbf{X}}$ may have mixed components, accounting for different sub-types in the cell population. The variation among $\mathbf{X}_c$ accounts for the biological variation of the data.

The gene expression level $\mathbf{X}_c$ is measured by the observed read counts $\mathbf{Y}_c$ via sequencing, for which the cell size factors γ_c that account for different sequencing depths across cells are assumed to be generated i.i.d. from some unknown distribution P_γ with an additional constraint that $\mathbb{E}[\gamma_c] = 1$. For each cell c, given the gene expression level $\mathbf{X}_c$ and the size factor γ_c, first the total number of reads of this cell follows a Poisson distribution $n_{\text{reads, c}} \sim \text{Poi}(\gamma_c n_{\text{reads}})$ *; second each read goes to gene g with probability X_{cg}. This hierarchical Poisson multinomial model can be equivalently written as an independent Poisson sampling model[4]:

$$Y_{cg} | X_{cg}, \gamma_c \sim \text{Poi}(\gamma_c n_{\text{reads}} X_{cg}), \ \forall\, c \in [n_{\text{cells}}],\ g \in [G]. \tag{5}$$

To summarize, the complete statistical model can be written as

$$\begin{aligned} &\mathbf{X}_c \overset{\text{i.i.d.}}{\sim} P_{\mathbf{X}}, \quad \gamma_c \overset{\text{i.i.d.}}{\sim} P_\gamma, \\ &Y_{cg} | X_{cg}, \gamma_c \sim \text{Poi}(\gamma_c n_{\text{reads}} X_{cg}), \ \forall\, c \in [n_{\text{cells}}],\ g \in [G]. \end{aligned} \tag{6}$$

As a brief history, the Poisson sampling model has been known in RNA-Seq literature for a long time[5] and is later brought into scRNA-seq[6]. Accompanying the basic Poisson model, some variations are later purposed and widely-adopted such as the negative binomial (gamma-Poisson, over-dispersion) model[7], beta-Poisson model[8] and the zero-inflated negative binomial model[9]. The model we consider here is an empirical Bayes model that incorporates the Poisson sampling model and follows Efron's G-modeling strategy[10]. It is first purposed for UMI-based scRNA-seq data by[11], where the model has been extensively validated on 9 datasets with different technology.

*Note that here n_{reads} has a slightly different interpretation to be the expected number reads per cell other than the actual average number of reads per cell. This does not make a big difference in practice since the two are very similar due to concentration of the Poisson distribution.

1.2 Computing sequencing depth and size factor for a scRNA-seq dataset

The sequencing depth n_{reads} and the size factor γ_c can be estimated with a much higher preconfidence intervalsion as compared to other quantities. Therefore unless otherwise specified, we treat them as known quantities in the analysis. The estimators for n_{reads} and γ_c can be written as

$$\widehat{n_{\text{reads}}} = \frac{1}{n_{\text{cells}}} \sum_{c=1}^{n_{\text{cells}}} n_{\text{reads},c}, \quad \hat{\gamma}_c = \frac{n_{\text{reads},c}}{\widehat{n_{\text{reads}}}}. \tag{7}$$

Here, the sequencing depth n_{reads} is estimated by a simple average of the entire read count matrix over the cells, which is very accurate since it utilizes all the data. The size factor γ_c, in spite of being a random variable, can be pinned down with a high accuracy given the data. Recall that the total number of reads for each cell follows a Poisson model $[n_{\text{reads}c}|\gamma_c, n_{\text{reads}}] \sim \text{Poi}(\gamma_c n_{\text{reads}})$. Hence when $\gamma_c n_{\text{reads}}$ is large, we know that $n_{\text{reads}c} \approx \gamma_c n_{\text{reads}}$. Since $n_{\text{reads}c}$ is observed and n_{reads} can be accurately estimated, γ_c can also be accurately estimated. In fact, as further elaborated in Remark 3 postponed to Supplementary Note 7, the estimation error is $\frac{1}{\sqrt{n_{\text{cells}} \vee n_{\text{reads}}}}$ with high probability. To put it in context, consider a typical scRNA-seq experiment where $n_{\text{cells}} \sim 5k$, $n_{\text{reads}} \sim 5k$, and $G \sim 30k$. Since $\sum_g X_{cg} = 1$, the expression level for each gene is roughly $X_{cg} \sim 1/G$. Then the read count for an entry $Y_{cg} \sim X_{cg} n_{\text{reads}} \sim n_{\text{reads}}/G$, which is usually a single-digit number and often zero. Hence, as compared to the estimation error of n_{reads} and γ_c, it is much more challenging to estimate the relative gene expression level X_{cg}.

Supplementary Note 2 Roadmap

We study the optimal sequencing budget allocation for estimating the following distributional quantities of $P_{\mathbf{X}}$ that are commonly-used in scRNA-seq analysis.

1. The k-th marginal moment for gene $g \in [G]$ with the definition

$$M_{k,g} = \mathbb{E}[X_{cg}^k] = \int x^k dP_{X_g}(x),\ \forall g \in [G]. \tag{8}$$

   The gene moments often play an important role in data pre-processing, feature selection, and gene-type identification. For example, the first moment (mean) can be used to filter out lowly expressed genes. The coefficient of variation (std/mean) and the Fano factor (var/mean), derived from the first and the second moment, are used to quantify the variability of genes and select important genes for further downstream analysis. Moreover, genes with a high mean and a small variance are often identified as housing keep genes. As a last example, the moments can also be utilized for differential expression (DE) analysis[12], where the second moment can be used to quantify the variability of the gene and hence giving a proper p-value. In addition, the DE analysis can go beyond testing different means under the two conditions. For example, one can consider testing different distributions under the two conditions[13], where the first few moments can serve as a proxy for the gene distribution.

2. The covariance matrix of the gene expression level $K \in \mathbb{R}^{G\times G}$. The covariance matrix is one of the most fundamental quantities for studying gene-gene relationship. First, spectrum methods like PCA, contrastive PCA (cPCA) or spectral clustering[14–16] can be run using the covariance matrix as input. Also, the Pearson correlation matrix, derived from the covariance matrix, can be used to quantify the gene-gene relationship, which can be further used for gene co-expression network analysis[17,18].

3. The inactive probability for gene g with the definition

$$p_{0,g}(\kappa) = \mathbb{E}[\exp(-\kappa X_{cg})] = \int \exp(-\kappa x) dP_{X_g}(x). \tag{9}$$

   The inactive probability is used to quantify to the proportion of cells where gene g is inactive and can be viewed as a proxy of the zero probability as discussed in[19]. In[19], the authors consider the probability that a gene has zero read count:

$$P(Y_{cg} = 0) \approx \int \exp(-n_{\text{reads}} x) dP_{X_g}(x) = p_{0,g}(n_{\text{reads}}). \tag{10}$$

   Our definition generalizes this to a range of quantities specified by different κ's. As a special case, κ=∞ corresponds to the probability that X_{cg} is zero. The smoothing function $\exp(-\kappa x)$ is used because even for a cell population where a gene is not supposed to express, we often observe some low expression level. Figure 20 shows such an example by visualizing the gene expression level of *CD3E*—a marker gene for T-cells—among different cell populations. *CD3E* is expressed in the center point cloud (dark) which corresponds to the T-cells. However, for other cell populations, the dots are not entirely white, indicating that *CD3E* is also detected in those cell populations. Hence a small value of X_{cg} should be treated in a similar way as X_{cg} being 0, both indicating the gene is inactive, while a large value of X_{cg} shows that the gene is active in this cell. The smoothing function $\exp(-\kappa x)$ interpolates the two cases, where κ specifies how small X_{cg} is that we should count it as being inactive.

4. The pairwise inactive probability for the gene pair $g_1 g_2$ with the definition

$$p_{0,g_1g_2}(\kappa) = \mathbb{E}[\exp(-\kappa(X_{cg_1} + X_{cg_2}))] \tag{11}$$

$$= \int \exp(-\kappa x_1)\exp(-\kappa x_2) dP_{X_{g_1g_2}}(x_1, x_2). \tag{12}$$

Similar to the previous case, the pairwise inactive probability quantifies how often the two genes are inactive (or equivalently active) at the same time. This quantity can be used to analyze the gene co-expression network, e.g., in[19].

5. The marginal gene distribution P_{X_g} for each gene. This quantity is considered in[11].

For all quantities listed above, we study the optimal budget split between the number of cells n_{cells} and the sequencing depth n_{reads} that achieves the smallest error. Interestingly we found that this optimal budget split is the same for all these quantities, which, in plain English, can be stated as follows. For any budget B, make n_{reads} some constant value and then let $n_{\text{cells}} = B/n_{\text{reads}}$, whereas this constant may depend on the quantities we consider to estimate but is independent of the sequencing budget B. Mathematically,

Theorem 1. *(Optimal budget allocation, informal) For estimating the gene moments, the covariance matrix, the inactive probability, the pair-wise inactive probability and the distribution, the optimal budget allocation is*

$$n_{\text{reads}} = \Theta(1), \text{ and } n_{\text{cells}} = \Theta(B). \tag{13}$$

This optimality is in the minimax sense over a family of distributions $P_{\mathbf{X}}$ with mild assumptions and the optimal error rate is achieved by the empirical Bayes (EB) estimators.

Theorem 1 suggests that for a scRNA-seq experiment, we should sequence a reasonably deep dataset while collecting as many cells as possible (see also the formal statement in Supplementary Note 5). Interestingly, it says that after the sequencing depth n_{reads} has achieved a certain level, a deeper sequencing does not help much as compared to having more cells. Then how deep is deep enough? In other words, how do we determine the constant inside the big theta term for determining the optimal depth n^*_{reads} for the experiment? This is further explored in Supplementary Note 5, where we argue that ~ 1 read per cell per gene on average guarantees a near-optimal error. This guarantee, however, comes with a condition that instead of the commonly-used plug-in estimator that estimates the gene distributional quantities using the corresponding quantities of the empirical read counts, we need to be a little smarter to develop some alternative estimators which we call the empirical Bayes (EB) estimators. These estimators are discussed in detail in Supplementary Note 4.

The supplementary material is organized as follows. After supplementary figures, Supplementary Note 3 discusses various concerns regarding the experimental design procedure. Supplementary Note 4 provides a high-level introduction on the empirical Bayes estimators whereas the detailed theoretical analysis is at Supplementary Note 5. Supplementary Note 6 contains miscellaneous information. Finally, the technical proofs are postponed to Supplementary Note 7.

Supplementary Note 3 Experimental Design

Theorem 1 implies that the optimal depth n^*_{reads} is a constant independent of the sequencing budget B. The exact value of such constant is further investigated in this section.

First we consider the widely-used overdispersion model, where for each gene g, the read counts Y_{cg} are assumed to follow a negative binomial distribution[7,20,21]. Since the negative binomial distribution can be derived as a gamma-Poisson mixture, the resulting model can be viewed as a special case of the hierarchical model (6) in which the underlying gene expression marginal distribution is the gamma distribution[†], i.e., $X_{cg} \sim \text{Gamma}(r_g, \theta_g)$. This is analyzed in detail in Supplementary Note 5, with the corresponding EB estimators $\hat{\theta}_g^{\text{EB}}$ and $\hat{r}_g^{\text{EB}}$ given in (53) and the estimation error in Lemma 2. Specifically, the estimation error can be written as

$$MSE(\hat{\theta}_g^{\text{EB}}) = \frac{1}{n_{\text{cells}}} \frac{2r_g+3}{r_g}\theta_g^2 + \frac{c_{\gamma,-1}}{B}\frac{4r_g+5}{r_g}\theta_g + \frac{c_{\gamma,-2}}{Bn_{\text{reads}}}\frac{2(r_g+1)}{r_g}, \tag{14}$$

$$MSE(\hat{r}_g^{\text{EB}}) = \frac{1}{n_{\text{cells}}} 2r_g(r_g+1) + \frac{c_{\gamma,-1}}{B}\frac{4r_g(r_g+1)}{\theta_g} + \frac{c_{\gamma,-2}}{Bn_{\text{reads}}}\frac{2r_g(r_g+1)}{\theta_g^2}, \tag{15}$$

where $c_{\gamma,-1} = \frac{1}{n_{\text{cells}}}\sum_{c=1}^{n_{\text{cells}}}\frac{1}{\gamma_c}$ and $c_{\gamma,-2} = \frac{1}{n_{\text{cells}}}\sum_{c=1}^{n_{\text{cells}}}\frac{1}{\gamma_c^2}$. For the case of estimating the gamma parameters, the exact value of the optimal depth n^*_{reads} can be derived from the above equations. Specifically, first we have that $c_{\gamma,-1} \sim c_{\gamma,-2} \sim 1$ since the size factors are close to 1. Second, the shape parameter r_g can be estimated via the mean-variance relationship regression (Supplementary Figure 3), where a value of $r_g = 1.5$ is typical. Third, the scale parameter θ_g satisfies the relationship $\theta_g = r_g/p$ for a gene with a mean expression level $\mathbb{E}[X_{cg}] = p$. Therefore, given the mean expression level p for the gene under consideration, the error curve can be computed from the above equations (Figure 1b main paper, error $= 0.5[MSE(\hat{\theta}_g^{\text{EB}}) + MSE(\hat{r}_g^{\text{EB}})]$). Here we have defined the error to be the average of $MSE(\hat{\theta}_g^{\text{EB}})$ and $MSE(\hat{r}_g^{\text{EB}})$ without loss of generality; using any one of the two terms yields a similar result. It can be further inferred that optimal depth is ~ 1 read per cell per gene for estimating the gamma parameters. Here we note that the ~ 1 read refers to the quantity pn_{reads}, i.e., the mean reads for the gene under consideration rather than n_{reads}, the UMIs for all genes in a cell.

Then how about estimating other quantities? Lemma 2 also shows that the optimal depth is around 1 read per cell on average for the gene under consideration for estimating the second moment. In addition, the simulation results (Figure 2a, Supplementary Figure 4) yield similar conclusions for estimating other quantities. Hence we conclude that for estimating a variety of quantities, the optimal depth is 1 read per cell for the gene under consideration. This result is then converted to an experimental design procedure for all genes (Figure 1c, main paper). For example, Supplementary Figure 2a suggests 7k UMIs per cell for the pbmc_4k dataset when the gene *MS4A1* is considered. Supplementary Figure 2f suggests 14k UMIs per cell for the brain_9k dataset when the gene *S100a10* is considered.

3.1 Feasibility of the recommended sequencing depth

Is the recommended sequencing depth realistic for the current single-cell sequencing technologies? Although 10x Genomics does not have an exact statistics publicly available, an investigation of related literatures yields an affirmative answer.

First, since different cells have different amount of mRNA contents, the maximum UMI per cell is different for different tissues. Based on the information available for the 10x datasets (saturation, repetitions of observed UMIs, etc...), the maximum number of UMIs per cell that can be sequenced by the 10x technology is estimated to be 7k for PBMCs, 25k for mice brain, and 100k for cell lines.

An orthogonal calculation shows a similar result. Specifically, the maximum sequencing depth can be estimated as

$$\text{maximum sequencing depth} = \text{total transcripts per cell} \times \text{UMI efficiency}. \tag{16}$$

[†]The constraint $X_{cg} < 1$ can be neglected here without loss of generality. This is because the relative expression X_{cg} is of the order $1/G$ which is much smaller than 1. With a mean much smaller than 1, the truncated gamma distribution with truncation at 1 is very close to the untruncated version.

For the total transcripts per cell, since there are roughly $N = 200$-400k transcripts (mRNAs) for a typical mammalian cell[22] and given that some cells (e.g., PBMCs) may have a much lower mRNA content, we can conservatively use $N = 100$-300k without loss of generality. For the UMI efficiency, it is reported to be 8% for the External RNA Controls Consortium synthetic RNAs (ERCCs) for the v1 chemistry (Figure 2d in[3]). However, it is found that the UMI efficiency for endogenous genes are usually much higher than that for ERCCs, since endogenous genes have longer poly(A) tails which makes it easier for cDNA conversion[23]. Considering the fact that the v2 chemistry is believed to have a higher UMI efficiency, a conservative estimate yields a UMI efficiency of 10-15% for sequencing endogenous genes for the v2 chemistry (currrent), which translates to a maximum of 10-45k UMIs per cell. In addition, it is reported that other technologies can potentially sequence at a much higher depth than the 10x technology[23].

Under the recommended sequencing depth (e.g., $n_{\text{reads}} = 14$k for brain_9k), the Poisson model (6) is a good approximation of the actual sequencing process. Consider a typical case where there are $N = 200$k mRNAs per cell and the UMI efficiency $\eta = 0.1$. For a gene (e.g., gene 1) with N_1 transcripts in the cell, its corresponding UMI counts, sequenced at the depth of n_{reads} UMIs per cell, follows a hypergeometric distribution

$$Y_{cg_1} \sim \text{hypergeometric}(n_{\text{reads}}, N_1, N), \tag{17}$$

where Y_{cg_1} can be understood as the number of transcripts sequenced for gene 1 while randomly selecting n_{reads} transcripts from a population of N transcripts containing N_1 transcripts for gene 1. We note that this essentially assumes that each transcript molecule is equally likely to be sequenced; such assumption is used in other works, e.g.,[11,24].

Since $n_{\text{reads}} = 14$k is roughly 7% of N, the hypergeometric distribution can be well approximated by the binomial distribution. While multiple genes are considered, the binomial distribution becomes the multinomial distribution, which can be approximated by the Poisson model according to previous discussions (Supplementary Note 1). In fact, since n_{reads} is smaller than the sequencing limit $\eta N \approx 0.1N$, the Poisson model is always a good approximation of the sequencing process. Such approximation may break only when the UMIs sequenced take up a much larger proportion of the entire transcript population in the cell, e.g., $n_{\text{reads}} > 0.3N$. This might happen when the UMI efficiency is significantly improved in the future.

Last but not the least, the UMI efficiency imposes a condition on the limit of the rare genes that can be reliably detected. Since we require one read per cell per gene for the optimal budget allocation and the UMI efficiency is 0.1-0.15, a gene needs to have at least $1/\eta = 7$-10 transcripts. The rarer genes are out of the scope for the experimental design for this paper.

Supplementary Note 4 Empirical Bayes Estimators

In this section we introduce the principles for designing empirical Bayes estimators for different distributional quantities. For simplicity let us ignore the size factor γ_c and the sequencing depth n_{reads} for now by considering the following statistical model:

$$\mathbf{X}_c \overset{\text{i.i.d.}}{\sim} P_{\mathbf{X}},\ Y_{cg}|X_{cg} \sim \text{Poi}(X_{cg}),\ \forall\, c \in [n_{\text{cells}}],\ g \in [G]. \tag{18}$$

We note that both quantities can be accurately estimated and thus can be easily added back later.

As a general recipe, the plug-in estimator uses the scaled (relative) read counts $\mathbf{Y}_1/n_{\text{reads}}, \mathbf{Y}_2/n_{\text{reads}}, \cdots$ as a proxy for the true relative gene expression levels $\mathbf{X}_1, \mathbf{X}_2 \cdots$ effectively estimating the corresponding distributional quantities by "plugging-in" the observed values. For example, the plug-in estimator estimates the mean of the gene expression distribution $P_{\mathbf{X}}$ by that of $P_{\mathbf{Y}/n_{\text{reads}}}$, the variance of $P_{\mathbf{X}}$ by that of $P_{\mathbf{Y}/n_{\text{reads}}}$, etc.

The problem with this is that the estimated results are usually overly variable due to the presence of the Poisson noise. Taking the variance estimation as an example, the plug-in estimated variance can be written as

$$\widehat{var}_g^{\text{plug-in}} = \frac{1}{n_{\text{cells}} - 1} \sum_{c=1}^{n_{\text{cells}}} (Y_{cg} - \bar{Y}_{cg})^2, \tag{19}$$

where $\bar{Y}_{cg}$ is the sample mean of Y_{cg}. How does this compare to the variance of X_{cg}? A simple calculation gives that

$$\mathbb{E}\left[\widehat{var}_g^{\text{plug-in}}\right] = \text{Var}[Y_{cg}] = \text{Var}[\mathbb{E}[Y_{cg}|X_{cg}]] + \mathbb{E}[\text{Var}[Y_{cg}|X_{cg}]] = \text{Var}[X_{cg}] + \mathbb{E}[X_{cg}]. \tag{20}$$

Note that there are two terms in the above equation; the first $\text{Var}[X_{cg}]$ corresponds to the true gene variance and the second $\mathbb{E}[X_{cg}]$ corresponds to the technical variation introduced by the Poisson noise. Then conceptually we can write

$$\text{plug-in estimate} = \text{biological truth} + \text{Poisson noise}, \tag{21}$$

from which we can see that the plug-in estimate is overly inflated by the Poisson noise.

The EB estimators in general refer to the estimators that are aware of the noise model (which is Poisson here) and correct the noise introduced by it. In the case of estimating the variance, the bias can be easily corrected by simply subtracting the mean, where the corresponding EB variance estimator can be written as

$$\widehat{var}_g^{\text{EB}} = \frac{1}{n_{\text{cells}} - 1} \sum_{c=1}^{n_{\text{cells}}} (Y_{cg} - \bar{Y}_{cg})^2 - \frac{1}{n_{\text{cells}}} \sum_{c=1}^{n_{\text{cells}}} Y_{cg}. \tag{22}$$

There is no general principle for designing EB estimators and they are usually developed in the literature in a case-by-case fashion. The EB estimators for quantities considered in this paper are summarized in Supplementary Table 1, where we added back the size factor γ_c as well as the sequencing depth n_{reads}. The corresponding plug-in estimators are also presented for comparison. For the inactive probability (and the pair-wise case), $a_{Y_{cg}}$ is a coefficient that depends on Y_{cg}, κ, and n_{reads}. The plug-in estimators are simply the empirical proportion of zero, but not the exponentially weighted sum by plugging in the empirical reads $\frac{Y_{cg}}{\gamma_c n_{\text{reads}}}$. This is because the inactive probability means to capture the proportion of cells where the corresponding gene is not active, and the former is more approriate for this purpose according to[19]. See corresponding parts in Supplementary Note 5 for details for those estimators.

As a brief history, the EB moment estimators are based on the property of the Poisson factorial moments and have often appeared in literatures on estimating properties of a large alphabet, e.g.,[4,25–27]. The pair-wise moment estimator is a direct extension of such. The EB inactive probability estimator adapts the Good-Toulmin estimator and the Efron-Thisted estimator[4,28,29], while the pair-wise case is a direct generalization. The EB distribution estimator adapts the maximum likelihood estimator accompanied with Efron's G-modeling strategy[10,30]. Some of these EB estimators are already used for scRNA-seq analysis, either explicitly or implicitly. For example, the bias introduced by the Poisson noise is corrected in[31] for estimating cv, Fano factor, and gene correlation, which corresponds to the EB estimators for the 2nd moment. In addition, the EB distribution estimator is purposed in[11], where an exponential-family semi-parametric EB model similar to (67) is assumed.

	plug-in	EB
1-st moment $M_{1,g}$	$\frac{1}{n_{\text{cells}}}\sum_{c=1}^{n_{\text{cells}}}\frac{Y_{cg}}{\gamma_c n_{\text{reads}}}$	same
2-nd moment $M_{2,g}$	$\frac{1}{n_{\text{cells}}}\sum_{c=1}^{n_{\text{cells}}}\frac{Y_{cg}^2}{(\gamma_c n_{\text{reads}})^2}$	$\frac{1}{n_{\text{cells}}}\sum_{c=1}^{n_{\text{cells}}}\frac{Y_{cg}^2-Y_{cg}}{(\gamma_c n_{\text{reads}})^2}$
k-th moment $M_{k,g}$	$\frac{1}{n_{\text{cells}}}\sum_{c=1}^{n_{\text{cells}}}\frac{Y_{cg}^k}{(\gamma_c n_{\text{reads}})^k}$	$\frac{1}{n_{\text{cells}}}\sum_{c=1}^{n_{\text{cells}}}\frac{\prod_{r=0}^{k-1}(Y_{cg}-r)}{(\gamma_c n_{\text{reads}})^k}$
1-st pair-wise moment M_{11,g_1g_2}	$\frac{1}{n_{\text{cells}}}\sum_{c=1}^{n_{\text{cells}}}\frac{1}{n_{\text{reads}}^2\gamma_c^2}Y_{cg_1}Y_{cg_2}$	same
inactively probability $p_{0,g}(\kappa)$	$\frac{1}{n_{\text{cells}}}\sum_{c=1}^{n_{\text{cells}}}\mathbb{I}_{\{Y_{cg}=0\}}$	$\frac{1}{n_{\text{cells}}}\sum_{c=1}^{n_{\text{cells}}}a_{Y_{cg}}$
pair-wise inactive prob. $p_{0,g_1g_2}(\kappa)$	$\frac{1}{n_{\text{cells}}}\sum_{c=1}^{n_{\text{cells}}}\mathbb{I}_{\{Y_{cg_1}=Y_{cg_2}=0\}}$	$\frac{1}{n_{\text{cells}}}\sum_{c=1}^{n_{\text{cells}}}a_{Y_{cg_1}}a_{Y_{cg_2}}$
distribution P_{X_g}	empirical distribution of Y_{cg} (scaled by $1/n_{\text{reads}}$)	$\hat{P}_{X_g}$ that most likely gives empirical distribution of Y_{cg} via model (6)

Supplementary Table 1. A comparison of the plug-in estimator and the EB estimator for estimating various quantities, where the two estimators are deliberately written in similar forms for the ease of comparison.

4.1 Relation to the overdispersion model

The EB framework also encompasses the overdispersion model that is widely-used in scRNA-seq. As a brief history, the overdispersion model is first proposed for bulk RNA-Seq. Taking the simplified model (18) as the working example. Since the sequencing process can be viewed as sampling a subset from a collection of transcripts, it can be modeled using the Poisson model[12,32], where a gene is assumed to have a fixed expression level x_g and the read counts are observed with a Poisson noise $Y_g \sim \text{Poi}(x_g)$. Such model imposes a condition that the mean and the variance of the observed read counts are equal, i.e., $\text{Var}(Y_g) = \text{mean}(Y_g)$. This, however, contradicts with what is observed in practice that the variance is often greater than the mean, i.e., $\text{Var}(Y_g) > \text{mean}(Y_g)$, which is referred to as the overdispersion phenomenon[20,21]. In order to address this issue, the overdispersion model assumes a negative binomial distribution for the read counts $Y_g \sim \text{NB}(r_g, p_g)$, which has the mean-variance relationship $\text{Var}(Y_g) = \text{mean}(Y_g) + \frac{1}{r_g}\text{mean}^2(Y_g)$ and $\alpha_g = \frac{1}{r_g}$ is call the overdispersion parameter[33,34]. This model is then adopted for scRNA-seq[7]. Later on, since the negative binomial (NB) model does not capture the dropouts well, a zero-inflated version is later purposed that incorporates a zero component into the negative binomial model[9].

Both the NB model and the zero-inflated NB model can be thought as special cases of the empirical Bayes model (18) considered in this paper. Let $\theta_g = \frac{p_g}{1-p_g}$. Then we can write the negative binomial model (NB) and the zero-inflated negative binomial (ZINB) model in the framework of EB model as

$$\text{NB}: Y_{cg} \sim \text{NB}(r_g, p_g) \Leftrightarrow X_{cg} \sim \text{Gamma}(r_g, \theta_g), Y_{cg}|X_{cg} \sim \text{Poi}(X_{cg}), \tag{23}$$

$$\text{ZINB}: Y_{cg} \sim p_{0,g}\delta_0 + (1-p_{0,g})\text{NB}(r_g, p_g) \Leftrightarrow \tag{24}$$

$$X_{cg} \sim p_{0,g}\delta_0 + (1-p_{0,g})\text{Gamma}(r_g, \theta_g), Y_{cg}|X_{cg} \sim \text{Poi}(X_{cg}). \tag{25}$$

In other words, they correspond to the empirical Bayes models with P_{X_g} defined to be the gamma distribution and the zero-inflated gamma distribution respectively. Both models make parametric assumptions on the gene distribution P_{X_g} in order to estimate distributional quantities like mean and variance. However, from the analysis of the EB framework we know that for estimating these quantities, such parametric assumptions are not necessary. To put it in another way, the fundamental part for scRNA-seq modelling is not the negative binomial distribution but the Poisson noise inside it.

The overdispersion parameter α_g is often estimated via method of moments (MOM) by

$$\hat{\alpha}_g = \frac{\widehat{\text{Var}}(Y_{cg}) - \widehat{\text{mean}}(Y_{cg})}{\widehat{\text{mean}}^2(Y_{cg})}, \tag{26}$$

where $\widehat{\text{mean}}(Y_{cg})$ and $\widehat{\text{Var}}(Y_{cg})$ are the empirical mean and variance of the read counts. Interestingly, the square-root

of this estimate corresponds to the cv of P_{X_g},

$$\sqrt{\hat{\alpha}_g} = \frac{\sqrt{\widehat{\mathrm{Var}}(Y_{cg}) - \widehat{\mathrm{mean}}(Y_{cg})}}{\widehat{\mathrm{mean}}(Y_{cg})} \approx \frac{\sqrt{\mathrm{Var}(X_{cg})}}{\mathrm{mean}(X_{cg})} = \mathrm{cv}(X_{cg}). \tag{27}$$

Since this relation is independent of the negative binomial model, $\sqrt{\hat{\alpha}_g}$ is still a good estimate of $\mathrm{cv}(X_{cg})$ even under the scenario of model mismatching. This probably explains why the mean-variance relationship in the negative binomial model is widely-used despite that the gamma distribution is likely to be inappropriate for modeling the underlying gene distribution.

4.2 Variation of the EB estimates

As a general phenomenon, the plug-in estimator adds an artificial inflation to the estimate and therefore reduces the relative variation. E.g., see the points in the middle left panel of Figure 3a and Supplementary Figures 8-9. Since the axes are in log scale and the EB estimates have smaller values, the absolute variations for the two estimates are not very different, which are proportional to $1/\sqrt{n_{\text{cells}}}$. It is only that the plug-in estimates have smaller relative fluctuations. However, the plug-in estimates are actually further away from the ground truth as compared to EB. This can be understood by considering the simplified model (18) in the context of estimating cv. In this case, the plug-in and the EB estimator can be written as

$$\widehat{cv}_g^{\text{plug-in}} = \frac{\sqrt{\widehat{\mathrm{Var}}(Y_{cg})}}{\widehat{\mathrm{mean}}(Y_{cg})}, \quad \widehat{cv}_g^{\text{EB}} = \frac{\sqrt{\widehat{\mathrm{Var}}(Y_{cg}) - \widehat{\mathrm{mean}}(Y_{cg})}}{\widehat{\mathrm{mean}}(Y_{cg})}, \tag{28}$$

where $\widehat{\mathrm{mean}}(Y_{cg})$ and $\widehat{\mathrm{Var}}(Y_{cg})$ are the sample mean and the sample variance of the read counts. For points in the bottom left corner in the figures, they have small cv values but relatively large mean expression levels, due to the fact that we only keep genes with mean counts larger than 0.1. As a result, $\mathrm{Var}(Y_{cg})$- $\mathrm{mean}(Y_{cg})$ = $\mathrm{Var}(X_{cg})$ is small while $\mathrm{mean}(Y_{cg})$ is relatively large. Hence, $\widehat{cv}_g^{\text{EB}}$ will fluctuate around a small value that is close to the true cv, while $\widehat{cv}_g^{\text{plug-in}} \sim \frac{1}{\sqrt{\mathrm{mean}(Y_{cg})}}$ will be a large value that is very different from the ground truth.

Supplementary Note 5 Optimal Budget Allocation

In this section we formally state and prove Theorem 1 while providing details for the design of the EB estimators. As a brief introduction of the minimax formulation[35] for the budget allocation problem, suppose we are interested in estimating some generic distributional quantity θ (determined by P_{X_g}) under the squared error $(\hat{\theta}-\theta)^2$. Since we do not know the gene distribution in prior, we would like to guarantee that this error is small for a large family of distributions $P_{X_g} \in \mathscr{P}$. Thus we should consider the worst-case error

$$\sup_{P_{X_g} \in \mathscr{P}} \mathbb{E}[(\hat{\theta}-\theta)^2]. \tag{29}$$

In order to make it small, we can choose the budget allocation $(n_{\text{cells}}, n_{\text{reads}})$ as well as the estimator $\hat{\theta}$, whereas the best of them yields the smallest error:

$$\inf_{n_{\text{cells}}, n_{\text{reads}}} \inf_{\hat{\theta}} \sup_{P_{X_g} \in \mathscr{P}} \mathbb{E}[(\hat{\theta}-\theta)^2], \tag{30}$$

which we call the minimax error or the minimax rate. Note that this minimax error is achieved by having the optimal budget allocation $(n^*_{\text{cells}}, n^*_{\text{reads}})$ and the optimal estimator $\hat{\theta}^*$ at the same time. Hence the optimal budget allocation is always accompanied with the corresponding optimal estimator, and when discussing one of them, one should always keep in mind this correspondence.

Before stating the main theorem, we define the big O notation[36] for asymptotic analysis in the regime where the budget $B \to \infty$. The big O notation is widely used in asymptotic analysis and can be briefly summarized as follows. For two sequences $\{x_n\}, \{y_n\}$ where $n = 1, 2, \cdots$,

$$x_n = O(y_n) \Leftrightarrow \exists\, c_1, n_0 > 0, \quad s.t.\ \forall n > n_0,\ x_n \le c_1 y_n, \tag{31}$$

$$x_n = \Theta(y_n) \Leftrightarrow \exists\, c_1, c_2, n_0 > 0, \quad s.t.\ \forall n > n_0,\ c_1 y_n \le x_n \le c_2 y_n, \tag{32}$$

$$x_n = \Omega(y_n) \Leftrightarrow \exists\, c_1, n_0 > 0, \quad s.t.\ \forall n > n_0\ x_n \ge c_1 y_n. \tag{33}$$

The formal statement of Theorem 1 is as follows.

Theorem 2. *Assume that there exist some constants $0 < c_0 < c_1 < \infty$ such that the size factor $\forall c, c_0 < \gamma_c < c_1$. Then the minimax rate for estimating moments, pairwise moments, inactive probability, pairwise inactively probability, and distribution are all $\Theta(\frac{1}{B})$. Namely,*

$$\inf_{n_{\text{cells}}, n_{\text{reads}}} \inf_{\hat{M}_{k,g}} \sup_{P_{X_g}} \mathbb{E}\left[\left(\hat{M}_{k,g} - M_{k,g}\right)^2\right] = \Theta(\frac{1}{B}) \tag{34}$$

$$\inf_{n_{\text{cells}}, n_{\text{reads}}} \inf_{\hat{M}_{11,g_1g_2}} \sup_{P_{X_{g_1g_2}}} \mathbb{E}\left[\left(\hat{M}_{11,g_1g_2} - M_{11,g_1g_2}\right)^2\right] = \Theta(\frac{1}{B}) \tag{35}$$

$$\inf_{n_{\text{cells}}, n_{\text{reads}}} \inf_{\hat{p}_{0,g}(\kappa)} \sup_{P_{X_g}} \mathbb{E}\left[\left(\hat{p}_{0,g}(\kappa) - p_{0,g}(\kappa)\right)^2\right] = \Theta(\frac{1}{B}) \tag{36}$$

$$\inf_{n_{\text{cells}}, n_{\text{reads}}} \inf_{\hat{p}_{0,g_1g_2}(\kappa)} \sup_{P_{X_{g_1g_2}}} \mathbb{E}\left[\left(\hat{p}_{0,g_1g_2}(\kappa) - p_{0,g_1g_2}(\kappa)\right)^2\right] = \Theta(\frac{1}{B}) \tag{37}$$

$$\inf_{n_{\text{cells}}, n_{\text{reads}}} \inf_{\hat{\alpha}} \sup_{P_{X_g} \in \mathscr{P}^{exp}} \mathbb{E}\left[\|\hat{\alpha} - \alpha\|_2^2\right] = \Theta(\frac{1}{B}), \tag{38}$$

where $\mathscr{P}^{exp}$ is an exponential family parametrized by α as defined later in (67). The minimax rates are achieved by the EB estimators (Supplementary Table 1 with details below) under the budget allocation

$$n_{\text{reads}} = \Theta(1), \quad n_{\text{cells}} = \Theta(B). \tag{39}$$

We follow the standard sandwich approach to prove Theorem 2. Specifically, suppose we want to show the minimax rate

$$\inf_{n_{\text{cells}},n_{\text{reads}}} \inf_{\hat{\theta}} \sup_{P_{X_g}\in\mathscr{P}} \mathbb{E}[(\hat{\theta}-\theta)^2] = \Theta(\frac{1}{B}). \tag{40}$$

We can do it via two steps. First we show that there exists an allocation $(n^*_{\text{cells}}, n^*_{\text{reads}})$ and an estimator $\hat{\theta}^*$ with which

$$\sup_{P_{X_g}\in\mathscr{P}} \mathbb{E}[(\hat{\theta}^*-\theta)^2] = O(\frac{1}{B}). \tag{41}$$

Second we show that for any allocation $(n^a_{\text{cells}}, n^a_{\text{reads}})$ and any estimator $\hat{\theta}^a$, the worst-case error

$$\sup_{P_{X_g}\in\mathscr{P}} \mathbb{E}[(\hat{\theta}^a-\theta)^2] = \Omega(\frac{1}{B}). \tag{42}$$

The minimax rate can then be proved by combining the two. We call the first step the upper bound and the second the lower bound. In the following sections, we first show the upper bounds for estimating different quantities separately and then lower bound in for all quantities, thus drawing the conclusion.

5.1 Moments and covariance

The kth moment of a gene g can be written as

$$M_{k,g} = \mathbb{E}[X_{cg}^k] = \int x^k dP_{X_g}(x).$$

Recall that the Poisson factorial moment has the property

Fact 1. *For a Poisson random variable $Z \sim \text{Poi}(\lambda)$, $\mathbb{E}\left[\prod_{r=0}^{k-1}(Z-r)\right] = \lambda^k$.*

Since $Y_{cg}|X_{cg} \sim \text{Poi}(\gamma_c n_{\text{reads}} X_{cg})$, Fact 1 suggests that

$$\mathbb{E}\left[\prod_{r=0}^{k-1}(Y_{cg}-r)\middle| X_{cg}\right] = (\gamma_c n_{\text{reads}} X_{cg})^k. \tag{43}$$

Then naturally, an unbiased estimator for $M_{k,g}$ can be derived by averaging the factorial moment over all cells:

$$\hat{M}^{\text{EB}}_{k,g} = \frac{1}{n_{\text{cells}}} \sum_{c=1}^{n_{\text{cells}}} \frac{1}{n^k_{\text{reads}}\gamma_c^k} \prod_{r=0}^{k-1}(Y_{cg}-r). \tag{44}$$

This also generalizes to the pairwise case. Suppose we are interested in estimating the first pairwise moment:

$$M_{11,g_1g_2} = \mathbb{E}[X_{cg_1}X_{cg_2}]. \tag{45}$$

Then the corresponding unbiased estimator can be written as

$$\hat{M}^{\text{EB}}_{11,g_1g_2} = \frac{1}{n_{\text{cells}}} \sum_{c=1}^{n_{\text{cells}}} \frac{1}{n^2_{\text{reads}}\gamma_c^2} Y_{cg_1}Y_{cg_2}. \tag{46}$$

We call these estimators the EB moment estimators and their performance can be characterized as below:

Lemma 1. *Assume that there exist some constants $0 < c_0 < c_1 < \infty$ such that the size factor $\forall c, c_0 < \gamma_c < c_1$. Then under the tradeoff (39), the worst-case mean square error (MSE)*

$$\sup_{P_{X_g}} \mathbb{E}\left[\left(\hat{M}^{\text{EB}}_{k,g} - M_{k,g}\right)^2\right] = O(\frac{1}{B}), \tag{47}$$

$$\sup_{P_{X_{g_1g_2}}} \mathbb{E}\left[\left(\hat{M}^{\text{EB}}_{11,g_1g_2} - M_{11,g_1g_2}\right)^2\right] = O(\frac{1}{B}). \tag{48}$$

The moments give rise to many quantities that are important to scRNA-seq analysis. Some of them are

$$\mathrm{Var}(X_{cg}) = M_{2,g} - M_{1,g}^2 \tag{49}$$

$$\mathrm{cv}(X_{cg}) = \frac{\sqrt{\mathrm{Var}(X_{cg})}}{M_{1,g}} = \frac{\sqrt{M_{2,g} - M_{1,g}^2}}{M_{1,g}} \tag{50}$$

$$\mathrm{Cov}(X_{cg_1}, X_{cg_2}) = M_{11,g_1g_2} - M_{1,g_1}M_{1,g_2} \tag{51}$$

$$\rho(X_{cg_1}, X_{cg_2}) = \frac{\mathrm{Cov}(X_{cg_1}, X_{cg_2})}{\sqrt{\mathrm{Var}(X_{cg_1})\mathrm{Var}(X_{cg_2})}} = \frac{M_{11,g_1g_2} - M_{1,g_1}M_{1,g_2}}{\sqrt{(M_{2,g_1} - M_{1,g_1}^2)(M_{2,g_2} - M_{1,g_2}^2)}}, \tag{52}$$

where ρ is the Pearson correlation. In addition, since all above quantities are smooth functions of the moments, according to the delta method[36], their worse-case MSE are also $O(\frac{1}{B})$.

Yet, one mysterious piece is the constants hidden inside the big theta terms in (39), which vary case-by-case for estimating different quantities. Can they be unrealistically large in spite of being constants? It is hard to derive such constant for the original budget allocation problem and for estimating a general quantity. However for the case of using EB estimators to estimate quantities like the first two moments or the gamma parameters, the closed-form expressions for optimal budget allocation are available.

As a recap, the EB estimators for the first and the second moment are given in (44). For assuming P_{X_g} to be the gamma distribution with the shape parameter r_g and the scale parameter θ_g, the EB estimators are obtained by plugging in the EB moment estimates:

$$\hat{\theta}_g^{\mathrm{EB}} = \frac{\hat{M}_{2,g}^{\mathrm{EB}}}{\hat{M}_{1,g}^{\mathrm{EB}}} - \hat{M}_{1,g}^{\mathrm{EB}}, \quad \hat{r}_g^{\mathrm{EB}} = \frac{(\hat{M}_{1,g}^{\mathrm{EB}})^2}{\hat{M}_{2,g}^{\mathrm{EB}} - (\hat{M}_{1,g}^{\mathrm{EB}})^2}. \tag{53}$$

We note that they also correspond to the common practice of first using the method of moments estimator (MOM) to estimate the negative binomial parameters and then converting the estimates to the corresponding gamma parameters based on the gamma-Poisson model.

Lemma 2. *(Exact budget allocation) Let* $c_{\gamma,-1} = \frac{1}{n_{\mathrm{cells}}}\sum_{c=1}^{n_{\mathrm{cells}}}\frac{1}{\gamma_c}$ *and* $c_{\gamma,-2} = \frac{1}{n_{\mathrm{cells}}}\sum_{c=1}^{n_{\mathrm{cells}}}\frac{1}{\gamma_c^2}$*. The MSE for estimating the first moment, the second moment, the gamma parameters using EB estimators are*

$$MSE(\hat{M}_{1,g}^{\mathrm{EB}}) = \frac{1}{n_{\mathrm{cells}}}Var(X_{cg}) + \frac{c_{\gamma,-1}}{B}M_{1,g}, \tag{54}$$

$$MSE(\hat{M}_{2,g}^{\mathrm{EB}}) = \frac{Var(X_{cg}^2)}{n_{\mathrm{cells}}} + \frac{c_{\gamma,-1}}{B}4M_{3,g} + \frac{c_{\gamma,-2}}{Bn_{\mathrm{reads}}}2M_{2,g}, \tag{55}$$

$$MSE(\hat{\theta}_g^{\mathrm{EB}}) = \frac{1}{n_{\mathrm{cells}}}\frac{2r_g+3}{r_g}\theta_g^2 + \frac{c_{\gamma,-1}}{B}\frac{4r_g+5}{r_g}\theta_g + \frac{c_{\gamma,-2}}{Bn_{\mathrm{reads}}}\frac{2(r_g+1)}{r_g}, \tag{56}$$

$$MSE(\hat{r}_g^{\mathrm{EB}}) = \frac{1}{n_{\mathrm{cells}}}2r_g(r_g+1) + \frac{c_{\gamma,-1}}{B}\frac{4r_g(r_g+1)}{\theta_g} + \frac{c_{\gamma,-2}}{Bn_{\mathrm{reads}}}\frac{2r_g(r_g+1)}{\theta_g^2}. \tag{57}$$

The optimal n_{reads} *that minimizes the above errors are respectively*

$$n_{\mathrm{reads}}^*(\hat{M}_{1,g}^{\mathrm{EB}}) \to 0, \quad n_{\mathrm{reads}}^*(\hat{M}_{2,g}^{\mathrm{EB}}) = \sqrt{\frac{2M_{2,g}c_{\gamma,-2}}{Var[X_{cg}^2]}}, \tag{58}$$

$$n_{\mathrm{reads}}^*(\hat{\theta}_g^{\mathrm{EB}}) = \sqrt{(1-\frac{1}{2r_g+3})\frac{c_{\gamma,-2}}{\theta_g^2}}, \quad n_{\mathrm{reads}}^*(\hat{r}_g^{\mathrm{EB}}) = \sqrt{\frac{c_{\gamma,-2}}{\theta_g^2}}. \tag{59}$$

We note that for the first moment, the error is $O(\frac{1}{B})$ as long as $n_{\mathrm{cells}} = \Omega(B)$, which includes the case where $n_{\mathrm{cells}} = \Theta(B)$. Hence the result is consistent with Theorem 2. Interestingly the result for the first moment implies

that the error always gets smaller when n_{cells} gets larger. In the limiting case where the error is minimized, there are infinitely many cells, each with almost zero reads, which corresponds to bulk RNA-Seq. This is consistent with the observation that bulk RNA-Seq has a good estimation of the mean gene expression level, but lacks a further resolution for the finer structures (say the second moment).

Since the size factors are usually close to 1, we have $c_{\gamma,-1} \approx c_{\gamma,-2} \approx 1$. Since there are G genes, for a typical gene $X_{cg} \sim \frac{1}{G}$ and therefore $\theta \sim \frac{1}{G}$, $M_2 \sim \frac{1}{G^2}$, $\text{Var}(X_{cg}^2) \sim \frac{1}{G^4}$. Also, the shape parameter $r \sim 1$. Therefore based on these approximations, the optimal allocations are

$$n^*_{\text{reads}}(\hat{M}_1^{\text{EB}}) \to 0, \quad n^*_{\text{reads}}(\hat{M}_2^{\text{EB}}) \sim \sqrt{2}G, \quad n^*_{\text{reads}}(\hat{\theta}^{\text{EB}}) \sim 0.9G, \quad n^*_{\text{reads}}(\hat{r}^{\text{EB}}) \sim G. \tag{60}$$

Then the mean read for this typical gene is $n^*_{\text{reads}} M_{1,g} \sim n^*_{\text{reads}}/G \sim 1$. In other words, the optimal budget allocation for this gene is around 1 read per cell for the four quantities considered above.

5.2 Inactive probability

Recall that for gene g, the inactive probability is defined as

$$p_{0,g}(\kappa) = \int \exp(-\kappa x) dP_{X_g}(x). \tag{61}$$

Define $t_c = \frac{\kappa}{n_{\text{reads}}\gamma_c}$ and an ancillary random variable $L \sim Bin(\lceil \frac{1}{2}\log_2 \frac{n_{\text{cells}}(t_c-1)^2}{t_c-2} \rceil, \frac{1}{t_c})$. Then the estimator can be constructed as follows:

$$\hat{p}^{\text{EB}}_{0,g}(\kappa) = \frac{1}{n_{\text{cells}}} \sum_{c=1}^{n_{\text{cells}}} (1-t_c)^{Y_{cg}} \min\left(1, \mathbb{I}_{\{t_c>2\}} P(L \geq Y_{cg})\right). \tag{62}$$

Remark 1. *The EB inactive probability estimator consists of two parts: $t_c \leq 2$ and $t_c > 2$. The first part is constructed via Taylor expansion, similar to the construction of the Good-Toulmin estimator[28]. The second part corresponds to the Efron-Thisted estimator[29]. When $t_c \leq 2$, $\hat{p}^{\text{EB}}_{0,g}(\kappa)$ is unbiased. When $t_c > 2$ and as it grows, the bias becomes larger and the estimation error also becomes larger. Supplementary Figure 21 in this note shows the bias of the estimator on approximating $\exp(-\kappa x)$ for a selection of t_c. The bias becomes large when $t_c = 5$. This is also reflected on the estimation error on Supplementary Figure 22, where the bias starts to dominate the error when $t_c > 5$. Empirically with $n_{\text{cells}} \sim 10000$, this estimator has a good performance when $t_c \leq 5$. One can refer to[4] for a comprehensive discussion of related estimators.*

Such estimator directly generalizes to the pairwise case, where we recall that the pairwise inactive probability is

$$p_{0,g_1g_2}(\kappa) = \int \exp(-\kappa x_1)\exp(-\kappa x_2) dP_{X_{g_1g_2}}(x_1,x_2). \tag{63}$$

The EB estimator for the pairwise inactive probability can be written as

$$\hat{p}^{\text{EB}}_{0,g_1g_2}(\kappa) = \frac{1}{n_{\text{cells}}} \sum_{c=1}^{n_{\text{cells}}} (1-t_c)^{Y_{cg_1}+Y_{cg_2}} \min\left(1, \mathbb{I}_{\{t_c>2\}} P(L \geq Y_{cg_1}) P(L \geq Y_{cg_2})\right) \tag{64}$$

Lemma 3. *Assume that there exist some constants $0 < c_0 < c_1 < \infty$ such that the size factor $\forall c, c_0 < \gamma_c < c_1$. Then under the tradeoff (39), the worst-case mean square error (MSE)*

$$\sup_{P_{X_g}} \mathbb{E}\left[\left(\hat{p}^{\text{EB}}_{0,g}(\kappa) - p_{0,g}(\kappa)\right)^2\right] = O(\frac{1}{B}), \tag{65}$$

$$\sup_{P_{X_{g_1g_2}}} \mathbb{E}\left[\left(\hat{p}^{\text{EB}}_{0,g_1g_2}(\kappa) - p_{0,g_1g_2}(\kappa)\right)^2\right] = O(\frac{1}{B}). \tag{66}$$

5.3 Distribution

Consider the distribution of gene g, P_{X_g}. Following the recipe in[10,11], we assume a zero-inflated p-parameter exponential family model for the distribution P_{X_g}, i.e.,

$$\mathscr{P}^{exp}: \; P_{X_g}(x;\alpha) = \exp\left(Q(x)\alpha - \phi(\alpha)\right), \tag{67}$$

where $Q(x) = [q_1(x), \cdots, q_p(x)]$, and $\alpha \in \mathbb{R}^p$. The first component $q_1(x) = \mathbb{I}_{\{x=0\}}$ models the zero inflation and $q_2(x), \cdots q_p(x)$ are the spline basis that are set to be 0 when $x = 0$, where we choose the degree to be 5. To make the problem computationally feasible, we assume that X_{cg} can be discretized as $X \in \mathscr{X} = \{x_1, \cdots, x_m\}$. We differ from previous works[10,11] by the way we choose the values in $\mathscr{X}$: instead of having points equally spaced between 0 and 1, we let them to space quadratically as $\mathscr{X} = \{\frac{1}{\eta}, \frac{2^2}{\eta}, \cdots, 1\}$ for some η. This is because the estimation becomes harder at the region closed to 0 and we need a finer grid there, which is inspired by[37]. We observed a significant performance improvement from such modification.

We infer the coefficients α via maximum likelihood, where the formula for the derivatives are given in[30] and we include them here for completeness. We denote the corresponding estimator by $\hat{\alpha}^{\text{EB}}$.

Remark 2. *(Maximum likelihood inference) For simplicity we omit the subscript "cg" in this section and use X, Y in places where we previously use X_{cg} and Y_{cg}. Since we have discretized X, we can define the m-dimensional probability mass vector $\mathbf{p}_X : [\mathbf{p}_X]_j = P_X(x_j;\alpha)$. We can also define the $m \times p$ matrix $Q : Q_{ji} = q_i(x_j)$. Note that they respect the relationship*

$$\mathbf{p}_X(\alpha) = \exp\left(Q\alpha - \phi(\alpha)\right).$$

Furthermore, define the conditional probability matrix $P_{Y|X} : [P_{Y|X}]_{kj} = P(Y = k|X = x_j)$. Then the probability of $Y = k$ can be written as

$$P(Y = k;\alpha) = f_k(\alpha) = \sum_{j=1}^{m} [P_{Y|X}]_{kj}[p_X]_j.$$

The log likelihood function for a single observation Y_c is

$$l_c(\alpha) = \log f_{Y_c}(\alpha) = \log \sum_{j=1}^{m} [P_{Y|X}]_{Y_c j}[p_X(\alpha)]_j,$$

and further the full log likelihood function is

$$l(\alpha) = \frac{1}{n_{\text{cells}}} \sum_{c=1}^{n_{\text{cells}}} l_i(\alpha) = \frac{1}{n_{\text{cells}}} \sum_{c=1}^{n_{\text{cells}}} \log \sum_{j=1}^{m} [P_{Y|X}]_{Y_c j}[p_X(\alpha)]_j. \tag{68}$$

We the maximum likelihood estimate $\hat{\alpha}^{\text{EB}}$ is obtained by optimizing

$$\hat{\alpha}^{\text{EB}} = \operatorname*{argmax}_{\alpha} l(\alpha). \tag{69}$$

Regarding this, some useful quantities are:

$$\dot{l}_c(\alpha) = Q^T W_{Y_c}(\alpha), \quad \mathscr{I}(\alpha) = Q^T \left[\sum_{k=1}^{\infty} f_k(\alpha) W_k W_k^T\right] Q,$$

where $w_{kj} = [P_X(\alpha)]_j \left(\frac{[P_{Y|X}]_{kj}}{f_k(\alpha)} - 1\right)$, and $W_k = [w_{k1}, \cdots, w_{km}]^T$.

Lemma 4. *(Distribution estimation) tradeoff For any distribution P_{X_g} in the exponential family (67), under the tradeoff (39) the EB distribution estimator $\hat{\alpha}^{\text{EB}}$ satisfies*

$$\mathbb{E}\left[\|\hat{\alpha}^{\text{EB}} - \alpha\|_2^2\right] = O(\frac{1}{B}).$$

5.4 Lower bound

In this section we give a lower bound for Lemma 1, 3, 4, and hence establishing the optimality of the tradeoff (39) as well as the optimal rate of the EB estimators. The lower bound is proved via Le Cam's two-point method[38] and is stated as below:

Lemma 5. *Under any tradeoff* $(n_{\text{reads}}, n_{\text{cells}})|_{n_{\text{reads}}n_{\text{cells}}=B}$ *and for any estimators, the worst-case error*

$$\sup_{P_{X_g}} \mathbb{E}\left[\left(\hat{M}_{k,g}^{\text{EB}} - M_{k,g}\right)^2\right] = \Omega(\frac{1}{B}) \tag{70}$$

$$\sup_{P_{X_{g_1g_2}}} \mathbb{E}\left[\left(\hat{M}_{11,g_1g_2}^{\text{EB}} - M_{11,g_1g_2}\right)^2\right] = \Omega(\frac{1}{B}) \tag{71}$$

$$\sup_{P_{X_g}} \mathbb{E}\left[\left(\hat{p}_{0,g}^{\text{EB}}(\kappa) - p_{0,g}(\kappa)\right)^2\right] = \Omega(\frac{1}{B}) \tag{72}$$

$$\sup_{P_{X_{g_1g_2}}} \mathbb{E}\left[\left(\hat{p}_{0,g_1g_2}^{\text{EB}}(\kappa) - p_{0,g_1g_2}(\kappa)\right)^2\right] = \Omega(\frac{1}{B}) \tag{73}$$

$$\sup_{P_{X_g} \in \mathscr{P}^{exp}} \mathbb{E}\left[\|\hat{\alpha}^{\text{EB}} - \alpha\|_2^2\right] = \Omega(\frac{1}{B}), \tag{74}$$

where the sup is over the exponential family $\mathscr{P}^{exp}$ *as defined in* (67) *for* (74) *and is over all distributions for others. Also we let* $\gamma_c = 1$ *for all* c *without loss of generality.*

Supplementary Note 6 Miscellaneous

6.1 Remark 3

Remark 3. *(Estimation error for estimating the sequencing depth n_{reads} and the size factor γ_c)*
The mean and the variance of $n_{\text{reads},c}$ can be computed as follows:

$$\mathbb{E}[n_{\text{reads},c}] = \mathbb{E}_{\gamma_c}\left[\mathbb{E}[n_{\text{reads},c}|\gamma_c]\right] = n_{\text{reads}} \tag{75}$$

$$Var[n_{\text{reads},c}] = \mathbb{E}_{\gamma_c}\left[Var[n_{\text{reads},c}|\gamma_c]\right] + Var_{\gamma_c}\left[\mathbb{E}[n_{\text{reads},c}|\gamma_c]\right] = n_{\text{reads}} + n_{\text{reads}}^2 Var[\gamma_c]. \tag{76}$$

Then the mean square error of $\widehat{n_{\text{reads}}}$ can be written as

$$\text{MSE}(\widehat{n_{\text{reads}}}) = \mathbb{E}[(\widehat{n_{\text{reads}}} - n_{\text{reads}})^2] = \frac{Var[n_{\text{reads},c}]}{n_{\text{cells}}} = \frac{n_{\text{reads}}}{n_{\text{cells}}} + \frac{n_{\text{reads}}^2 Var[\gamma_c]}{n_{\text{cells}}}. \tag{77}$$

Therefore

$$\widehat{n_{\text{reads}}} = n_{\text{reads}}\left[1 + O_p\left(\sqrt{\frac{Var[\gamma_c]}{n_{\text{cells}}}}\right)\right]. \tag{78}$$

We also know $[n_{\text{reads},c}|\gamma_c, n_{\text{reads}}] \sim \text{Poi}(\gamma_c n_{\text{reads}})$, giving that $[n_{\text{reads},c}|\gamma_c, n_{\text{reads}}] = \gamma_c n_{\text{reads}}(1 + O_p(\frac{1}{\sqrt{\gamma_c n_{\text{reads}}}}))$. Therefore,

$$[\hat{\gamma}|\gamma_c, n_{\text{reads}}] = \gamma_c\left[1 + O_p\left(\sqrt{\frac{Var[\gamma_c]}{n_{\text{cells}}}} + \frac{1}{\sqrt{\gamma_c n_{\text{reads}}}}\right)\right]. \tag{79}$$

A careful inspection of (77) and (79) reveals that the estimation error of (7) is around $\frac{1}{\sqrt{n_{\text{cells}} \vee n_{\text{reads}}}}$.

6.2 Algorithm efficiency

We implement the EB estimators in an efficient way that can be applied on very-large-scale scRNA-seq datasets like the brain_1.3m dataset, which is computationally prohibitive for many scRNA-seq algorithms available. We benchmark the running time for estimating the moment, the pairwise moment, the inactive probability, and the pairwise inactive probability on three datasets with various sizes, i.e., pbmc_4k, brain_9k, brain_1.3m. For these datasets, we use the top 4000 genes and estimate the related quantities, since most scRNA-seq analysis are performed using less than 4000 genes. We report the average running time over 5 repetitions along with the standard deviation. The data loading time via scanpy[2], an efficient python implementation of popular scRNA-seq algorithms, is also reported for comparison. The reported time is for loading the data for a second time after the cache file was built, which is much faster than the first time. All algorithms are run using single core (CPU model: AMD Opteron(tm) Processor 6378) and can be run on a laptop. We do not include distribution estimation since usually the distribution estimation is done on a few genes but not all of them, which presents a smaller challenge for computational efficiency.

The results are shown in Supplementary Table 2, where we see it is very efficient for estimating the 1d quantities like the moments and the inactive probability. Estimating the covariance and the pairwise inactive probability takes a longer time but is still within a feasible range, i.e., $\sim$ 12 hours for the brain_1.3m dataset. In practice to speed up the analysis process, we suggest to first estimate the 1d quantities to select a smaller subset of genes, and estimate more computationally-demanding quantities using only this smaller subset.

6.3 Definition of errors in simulations

The error used in the simulations (Figure 2a, Supplementary Figures 4-6) are defined as follows. Suppose $\mathbf{x}$ is the true parameter and $\hat{\mathbf{x}}$ is the estimated value, which can be scalar, vector, or matrix. Then the errors are

$$\text{log10 relative error} = \log_{10}\left(\frac{\|\mathbf{x} - \hat{\mathbf{x}}\|^2}{\|\mathbf{x}\|^2}\right) \tag{80}$$

$$\text{log10 cosine distance} = \log_{10}\left(1 - \left|\frac{\mathbf{x}^T\hat{\mathbf{x}}}{\sqrt{\|\mathbf{x}\|^2\|\hat{\mathbf{x}}\|^2}}\right|\right). \tag{81}$$

	pbmc_4k	brain_9k	brain_1.3m
data loading	0.76	1.99	131.00
moment	0.033(1e-4)	0.14(1e-3)	17.10(0.36)
covariance	27.76(1.65)	156.53(6.34)	15001.07(321.21)
inactive probability	0.46(4e-3)	1.23(0.02)	96.23(6.03)
pairwise inactive prob.	239.30(4.73)	642.41(0.91)	44887.18 (1276.25)

Supplementary Table 2. Computation time for algorithms in `sceb`. mean (std) (seconds)

For the W1 distance, first the two distributions are matched to have mean 1. Then the function sp.stats.wasserstein_distance in scipy[39] is called to computed the standard W1 distance.

6.4 Subsampling experiment

Here we describe the procedure of subsampling from a dataset. Suppose the full dataset has n_{cells} cells with $n_{\text{reads},c}$ reads for cell c and for the target subsampled dataset, we want $\tilde{n}_{\text{cells}} < n_{\text{cells}}$ cells with $\tilde{n}_{\text{cells},c} < n_{\text{reads},c}$ reads for cell c. First, we sample $\tilde{n}_{\text{cells}}$ cells from the overall n_{cells} cells uniformly at random without replacement. Next for each cell c, we first expand the read counts for the G genes into $n_{\text{cells},c}$ reads. Then we sample $\tilde{n}_{\text{cells},c}$ reads from the total $n_{\text{cells},c}$ reads uniformly at random without replacement. Last, we collapse the $\tilde{n}_{\text{cells},c}$ reads into the read counts for the subsampled data. It is not hard to show that if the full data follows a multinomial distribution with parameters $n_{\text{cells},c}$ and $\mathbf{x}_c$, then the new data follows a multinomial distribution with parameters $\tilde{n}_{\text{cells},c}$ and $\mathbf{x}_c$.

6.5 Gene module identification

The gene modules are identified based on the marker genes[40], gene pathways[41] and previous studies on PBMCs[3,19,42,43]. Compared to the gene network in a recent study based on the same dataset (pbmc_4k, Figure 2 in[19]), the gene network in this paper missed the module corresponding to the hematopoietic stem cells, but recovered additional modules that correspond to megakaryocytes, ribosomal proteins, and mitochondrially encoded protein coding genes; the megakaryocytes are reported in[3] while the latter two correspond to important house-keeping gene functions.

For Figure 4c right, there are 1054 gene pairs (of 217 distinct genes) where the EB estimates are significantly larger than the plug-in estimates (> 0.7). Out of this 1054 connections, 91 are also annotated in STRING with a score larger than 0.4 (medium confidence)[44]. Since STRING altogether annotated 842 connections out of 23246 possible connections of these 217 genes. The p-value that these 1054 gene pairs are selected at random is calculated to be 4.2e-11 based on the hypergeometric distribution with one-sided alternative (over-represent). Last, the biological interpretation of LY86 is based on[45,46].

6.6 Details of the smFISH experiments

Since the number of UMIs per cell is small as compared to the 10x datasets, we found that it is no longer a good assumption that the size factor γ_c can be estimated with high accuracy. Hence, in these two experiments, we treat them as random variables instead of given fixed numbers. Specifically, following model (6) and by Fact 1,

$$\mathbb{E}\left[\prod_{r=0}^{k-1}(Y_{cg}-r)\middle| X_{cg},\gamma_c\right] = (\gamma_c n_{\text{reads}} X_{cg})^k. \tag{82}$$

Taking expectation of both sides to have

$$\mathbb{E}\left[\prod_{r=0}^{k-1}(Y_{cg}-r)\right] = \mathbb{E}[(\gamma_c n_{\text{reads}})^k]\mathbb{E}[X_{cg}^k]. \tag{83}$$

Hence, the kth moment can be alternatively estimated as

$$\hat{M}_k^{\text{alt}} = \frac{\frac{1}{n_{\text{cells}}}\sum_{c=1}^{n_{\text{cells}}}\prod_{r=0}^{k-1}(Y_{cg}-r)}{\frac{1}{n_{\text{cells}}}\sum_{c=1}^{n_{\text{cells}}} n_{\text{reads},c}^k}. \tag{84}$$

For the smFISH data, we assume the following model

$$\begin{aligned} &\mathbf{X}_c \overset{\text{i.i.d.}}{\sim} P_{\mathbf{X}}, \quad \gamma_c \overset{\text{i.i.d.}}{\sim} P_\gamma, \\ &Y_{cg} = X_{cg}, \gamma_c, \ \forall\ c \in [n_{\text{cells}}],\ g \in [G]. \end{aligned} \tag{85}$$

As a result, the kth moment can be estimated as

$$\hat{M}_k^{\text{smFISH}} = \frac{\frac{1}{n_{\text{cells}}} \sum_{c=1}^{n_{\text{cells}}} Y_{cg}^k}{\frac{1}{n_{\text{cells}}} \sum_{c=1}^{n_{\text{cells}}} n_{\text{reads},c}^k}. \tag{86}$$

For the inactive probability, the smFISH ground truth is obtained by subsampling the smFISH data to have κ reads per cell on average and then computing the empirical zero proportion.

6.7 Details of the ERCC experiments

In Supplementary Figure 15, three ERCC datasets are analyzed, i.e., Zheng (1,015 cells)[3], Klein (953 cells)[31], Svensson (2,000 cells)[23]. Each of them contains 92 ERCC synthetic spike-in RNAs (Thermo Fisher Scientific), where the Zheng data uses Mix2 while others use Mix1. Expected ERCC molecule counts are calculated based on the amount of ERCC molecules used and sample dilution factors. The dilution factors are 340 for the Zheng data, 5000 for the Klein data, and 133 for the Svensson data, respectively. We would like to point out that there are two minor mistakes made in[11] regarding the Zheng data. First, Mix2 was used in the Zheng data instead of Mix1. Second, for the dilution factor, an extra factor of 40 needs to be considered since $3\mu L$ of the diluted ERCC solution is added to $100\mu L$ master mix according to the 10x protocal[‡]. After this two corrections, we are able to reproduce the exact figure as shown in the original paper (Figure 2d in[3]).

The post-hoc thresholds for the mean and the cv are obtained from the post-hoc table (Figure 2b, the column for 1,000 cells). For the inactive probability, the post-hoc threshold is 0.5 UMI per cell and is derived as follows. First for each ERCC spike-in, since the numbers of molecules are expected to be the same for all cells, the actual proportion of zero counts is 0. Due to the Poisson sequencing noise, an ERCC spike-in with an average depth of 2.5 UMIs per cell roughly yields 10% of zero counts ($\exp -2.5 \approx 0.1$), i.e., an error of 0.1. Recall that according to the interpretation in the Methods section, the EB inactive probability estimator estimates the proportion of zero counts when sequenced κ/n_{reads} times deeper. Thus, $\kappa = 5n_{\text{reads}}$ gives that at the depth of 2.5/5=0.5 UMI per cell, the EB inactive probability estimator should estimate the zero proportion at 2.5 UMIs per cell, i.e., yielding an error of 0.1. Hence, 0.5 UMI per cell is used as the post-hoc threshold.

Supplementary Figure 16 is based on the Klein data that also contains pure RNA controls; the variance and cv of them are expected to be very small or close to zero.

Supplementary Figure 17 is based on the Svensson data, which has relatively more cells (2,000) to subsample from. The procedure of the experiment is identical to that for the smFISH data (Figure 5b, Supplementary Figure 14) and the details are described in the figure caption.

[‡] https://community.10xgenomics.com/t5/Single-Cell-Forum/Thoughts-on-ERCC-Spike-Ins/td-p/207

Supplementary Note 7 Auxiliary Lemmas and Proofs

7.1 Proof of Theorem 2

Proof. The upper bound is shown by Lemma 1, Lemma 3 and Lemma 4 respectively. The lower bound is shown by Lemma 5. Then the conclusion can be drawn by the sandwich approach. □

7.2 Proof of Lemma 1

Proof. **We first show** (47). Consider the tradeoff (39), any distribution P_{X_g}, and any size factor $\{\gamma_c\}_{c=1}^{n_{\text{cells}}}$ that satisfies the assumption in Lemma 1. The bias and the variance can be computed as

$$bias[\hat{M}_{k,g}^{\text{EB}}] = \mathbb{E}[\hat{M}_{k,g}^{\text{EB}}] - M_{k,g} = \frac{1}{n_{\text{cells}}}\sum_{c=1}^{n_{\text{cells}}}\mathbb{E}\left[\frac{1}{n_{\text{reads}}^k\gamma_c^k}\mathbb{E}\left[\prod_{r=0}^{k-1}(Y_{cg}-r)\middle|X_{cg}\right]\right] - M_{k,g}$$

$$= \frac{1}{n_{\text{cells}}}\sum_{c=1}^{n_{\text{cells}}}\mathbb{E}\left[x_{cg}^k\right] - M_{k,g} = 0,$$

where the third equality is due to Fact 1. The variance

$$var[\hat{M}_{k,g}^{\text{EB}}] = \frac{1}{n_{\text{cells}}^2}\sum_{c=1}^{n_{\text{cells}}}\text{Var}\left[\frac{1}{n_{\text{reads}}^k\gamma_c^k}\prod_{r=0}^{k-1}(Y_{cg}-r)\right] \le \frac{1}{n_{\text{cells}}^2}\sum_{c=1}^{n_{\text{cells}}}\mathbb{E}\left[\frac{Y_{cg}^{2k}}{n_{\text{reads}}^{2k}\gamma_c^{2k}}\right]$$

$$= \frac{1}{n_{\text{cells}}^2}\sum_{c=1}^{n_{\text{cells}}}\mathbb{E}\left[\mathbb{E}\left[\frac{Y_{cg}^{2k}}{n_{\text{reads}}^{2k}\gamma_c^{2k}}\middle|X_{cg}\right]\right] = \frac{1}{n_{\text{cells}}^2}\sum_{c=1}^{n_{\text{cells}}}\mathbb{E}\left[\frac{1}{n_{\text{reads}}^{2k}\gamma_c^{2k}}\sum_{l=0}^{2k}c_{2k,l}(X_{cg}n_{\text{reads}}\gamma_c)^l\right],$$

where $c_{2k,l} = S(2k,l)$ is the Stirling numbers of the second kind, a constant that depends on $2k$ and l. Since γ_c can be treated as a constant in the asymptotic analysis and so is n_{reads} due to (39), we further conclude that

$$var[\hat{M}_{k,g}^{\text{EB}}] = O\left(\frac{1}{n_{\text{cells}}}\right),$$

and finally, by letting $n_{\text{cells}} = \Theta(B)$, the MSE of the $\hat{M}_{k,g}^{\text{EB}}$

$$MSE[\hat{M}_{k,g}^{\text{EB}}] = bias[\hat{M}_{k,g}^{\text{EB}}]^2 + var[\hat{M}_{k,g}^{\text{EB}}] = O\left(\frac{1}{B}\right).$$

We next show (48). Again consider the tradeoff (39), any distribution $P_{X_{g_1g_2}}$, and any size factor $\{\gamma_c\}_{c=1}^{n_{\text{cells}}}$ that satisfies the assumption in Lemma 1. The bias and the variance can be computed as follows.

$$bias[\hat{M}_{11,g_1g_2}^{\text{EB}}] = \mathbb{E}[\hat{M}_{11,g_1g_2}^{\text{EB}}] - M_{11,g_1g_2}$$

$$= \frac{1}{n_{\text{cells}}}\sum_{c=1}^{n_{\text{cells}}}\mathbb{E}\left[\frac{1}{n_{\text{reads}}^2\gamma_c^2}\mathbb{E}\left[Y_{cg_1}Y_{cg_2}\middle|X_{cg_1},X_{cg_2}\right]\right] - M_{11,g_1g_2}$$

$$= \frac{1}{n_{\text{cells}}}\sum_{c=1}^{n_{\text{cells}}}\mathbb{E}\left[X_{cg_1}X_{cg_2}\right] - M_{11,g_1g_2} = 0.$$

The variance

$$var[\hat{M}_{11,g_1g_2}^{\text{EB}}] = \frac{1}{n_{\text{cells}}^2}\sum_{c=1}^{n_{\text{cells}}}\text{Var}\left[\frac{1}{n_{\text{reads}}^2\gamma_c^2}Y_{cg_1}Y_{cg_2}\right] \le \frac{1}{n_{\text{cells}}^2}\sum_{c=1}^{n_{\text{cells}}}\mathbb{E}\left[\frac{Y_{cg_1}^2Y_{cg_2}^2}{n_{\text{reads}}^4\gamma_c^4}\right]$$

$$= \frac{1}{n_{\text{cells}}^2}\sum_{c=1}^{n_{\text{cells}}}\mathbb{E}\left[\mathbb{E}\left[\frac{Y_{cg_1}^2Y_{cg_2}^2}{n_{\text{reads}}^4\gamma_c^4}\middle|X_{cg_1},X_{cg_2}\right]\right]$$

$$= \frac{1}{n_{\text{cells}}^2}\sum_{c=1}^{n_{\text{cells}}}\mathbb{E}\left[\left(X_{cg_1}^2+\frac{X_{cg_1}}{n_{\text{reads}}\gamma_c}\right)\left(X_{cg_2}^2+\frac{X_{cg_2}}{n_{\text{reads}}\gamma_c}\right)\right] = O\left(\frac{1}{n_{\text{cells}}}\right).$$

By letting $n_{\text{cells}} = \Theta(B)$, the MSE of $\hat{M}^{\text{EB}}_{11,g_1g_2}$

$$MSE[\hat{M}^{\text{EB}}_{11,g_1g_2}] = bias[\hat{M}^{\text{EB}}_{11,g_1g_2}]^2 + var[\hat{M}^{\text{EB}}_{11,g_1g_2}] = O\left(\frac{1}{B}\right).$$

□

7.3 Proof of Lemma 2

Proof. We consider the first moment, the second moment, and the gamma parameters in order as follows. We omit the gene subscript g for simplicity.

The first moment. The MSE of the first moment

$$MSE(\hat{M}^{\text{EB}}_1) = \text{Var}(\hat{M}^{\text{EB}}_1) = \frac{1}{n^2_{\text{cells}}}\sum_{c=1}^{n_{\text{cells}}} \text{Var}\left[\frac{Y_{cg}}{n_{\text{reads}}\gamma_c}\right] \tag{87}$$

$$= \frac{1}{n^2_{\text{cells}}}\sum_{c=1}^{n_{\text{cells}}} \left[\text{Var}[\mathbb{E}[\frac{Y_{cg}}{n_{\text{reads}}\gamma_c}|X_{cg}]] + \mathbb{E}[\text{Var}[\frac{Y_{cg}}{n_{\text{reads}}\gamma_c}|X_{cg}]]\right] \tag{88}$$

$$= \frac{1}{n^2_{\text{cells}}}\sum_{c=1}^{n_{\text{cells}}} \left[\text{Var}[X_{cg}] + \mathbb{E}[\frac{X_{cg}}{n_{\text{reads}}\gamma_c}]\right] = \frac{1}{n_{\text{cells}}}\text{Var}(X_{cg}) + \frac{M_1}{B}\frac{1}{n_{\text{cells}}}\sum_{c=1}^{n_{\text{cells}}}\frac{1}{\gamma_c}, \tag{89}$$

where $c_{\gamma,-1} = \frac{1}{n_{\text{cells}}}\sum_{c=1}^{n_{\text{cells}}}\frac{1}{\gamma_c}$. Also it is not hard to see that the above decrease as n_{reads} decreases. Hence $n^*_{\text{reads}}(\hat{M}^{\text{EB}}_1) \to 0$.

The second moment. The MSE of the second moment

$$MSE(\hat{M}^{\text{EB}}_2) = \text{Var}(\hat{M}^{\text{EB}}_2) = \frac{1}{n^2_{\text{cells}}}\sum_{c=1}^{n_{\text{cells}}} \text{Var}\left[\frac{Y_{cg}(Y_{cg}-1)}{n^2_{\text{reads}}\gamma^2_c}\right]$$

$$= \frac{1}{n^2_{\text{cells}}}\sum_{c=1}^{n_{\text{cells}}} \left\{\text{Var}\left[\mathbb{E}\left[\frac{Y_{cg}(Y_{cg}-1)}{n^2_{\text{reads}}\gamma^2_c}\middle|X_{cg}\right]\right] + \mathbb{E}\left[\text{Var}\left[\frac{Y_{cg}(Y_{cg}-1)}{n^2_{\text{reads}}\gamma^2_c}\middle|X_{cg}\right]\right]\right\}.$$

We calculate the two terms separately. For the first term

$$\text{Var}\left[\mathbb{E}\left[\frac{Y_{cg}(Y_{cg}-1)}{n^2_{\text{reads}}\gamma^2_c}\middle|X_{cg}\right]\right] = \text{Var}[X^2_{cg}].$$

The second term

$$\mathbb{E}\left[\text{Var}\left[\frac{Y_{cg}(Y_{cg}-1)}{n^2_{\text{reads}}\gamma^2_c}\middle|X_{cg}\right]\right] = \mathbb{E}\left[\mathbb{E}\left[\frac{Y^2_{cg}(Y_{cg}-1)^2}{n^4_{\text{reads}}\gamma^4_c}|X_{cg}\right] - \mathbb{E}^2\left[\frac{Y_{cg}(Y_{cg}-1)}{n^2_{\text{reads}}\gamma^2_c}|X_{cg}\right]\right]$$

$$= \mathbb{E}\left[\left(X^4_{cg} + 4\frac{X^3_{cg}}{n_{\text{reads}}\gamma_c} + 2\frac{X^2_{cg}}{n^2_{\text{reads}}\gamma^2_c}\right) - (X^2_{cg})^2\right] = 4\frac{M_3}{n_{\text{reads}}\gamma_c} + 2\frac{M_2}{n^2_{\text{reads}}\gamma^2_c}.$$

Putting the two terms together we get

$$MSE(\hat{M}_2) = \frac{1}{n^2_{\text{cells}}}\sum_{c=1}^{n_{\text{cells}}}\left[\text{Var}[X^2_{cg}] + 4\frac{M_3}{n_{\text{reads}}\gamma_c} + 2\frac{M_2}{n^2_{\text{reads}}\gamma^2_c}\right] \tag{90}$$

$$= \frac{\text{Var}[X^2_{cg}]}{n_{\text{cells}}} + \frac{4M_3}{B}\frac{1}{n_{\text{cells}}}\sum_{c=1}^{n_{\text{cells}}}\frac{1}{\gamma_c} + \frac{2M_2}{Bn_{\text{reads}}}\frac{1}{n_{\text{cells}}}\sum_{c=1}^{n_{\text{cells}}}\frac{1}{\gamma^2_c}, \tag{91}$$

where $c_{\gamma,-1} = \frac{1}{n_{\text{cells}}}\sum_{c=1}^{n_{\text{cells}}}\frac{1}{\gamma_c}$ and $c_{\gamma,-2} = \frac{1}{n_{\text{cells}}}\sum_{c=1}^{n_{\text{cells}}}\frac{1}{\gamma^2_c}$. Further $MSE(\hat{M}^{\text{EB}}_2)$ is minimized by

$$n^*_{\text{reads}}(g) = \sqrt{\frac{2M_2c_{\gamma,-2}}{\text{Var}[X^2_{cg}]}}, \quad n^*_{\text{cells}}(g) = B\sqrt{\frac{\text{Var}[X^2_{cg}]}{2M_2c_{\gamma,-2}}}.$$

The gamma parameters. Let $X_{cg} \sim \text{Gamma}(r,\theta)$, for the shape parameter r and scale parameter θ. Then the moments (of this gamma distribution) are $M_k = \theta^k \frac{(r+k-1)!}{(r-1)!}$ with special cases

$$M_1 = r\theta \tag{92}$$
$$M_2 = r(r+1)\theta^2 \tag{93}$$
$$M_3 = r(r+1)(r+2)\theta^3 \tag{94}$$
$$M_4 = r(r+1)(r+2)(r+3)\theta^4 \tag{95}$$
$$\text{Var}(X_{cg}) = r\theta^2 \tag{96}$$
$$\text{Var}(X_{cg}^2) = 2(2r+3)(r+1)r\theta^4. \tag{97}$$

First the EM moment estimators follow an asymptotic normal distribution

$$\sqrt{n_{\text{cells}}}\begin{bmatrix} \hat{M}_1^{\text{EB}} - M_1 \\ \hat{M}_2^{\text{EB}} - M_2 \end{bmatrix} \sim \mathcal{N}\left(\begin{bmatrix} 0 \\ 0 \end{bmatrix}, \Sigma\right).,\tag{98}$$

with elements of Σ written as

$$\Sigma_{11} = \frac{1}{n_{\text{cells}}}\sum_{c=1}^{n_{\text{cells}}} \text{Var}[\frac{1}{n_{\text{reads}}\gamma_c}Y_{cg}] = \text{Var}(X_{cg}) + \frac{c_{\gamma,-1}}{n_{\text{reads}}}M_1 = r\theta^2 + \frac{c_{\gamma,-1}}{n_{\text{reads}}}r\theta \tag{99}$$
$$\Sigma_{12} = \frac{1}{n_{\text{cells}}}\sum_{c=1}^{n_{\text{cells}}} \text{Cov}[\frac{1}{n_{\text{reads}}\gamma_c}Y_{cg}, \frac{1}{n_{\text{reads}}^2\gamma_c^2}\left(Y_{cg}^2 - Y_{cg}\right)] = M_3 - M_1M_2 + 2\frac{c_{\gamma,-1}}{n_{\text{reads}}}M_2 \tag{100}$$
$$= 2r(r+1)\theta^3 + 2\frac{c_{\gamma,-1}}{n_{\text{reads}}}r(r+1)\theta^2 \tag{101}$$
$$\Sigma_{22} = \frac{1}{n_{\text{cells}}}\sum_{c=1}^{n_{\text{cells}}} \text{Var}[\frac{1}{n_{\text{reads}}^2\gamma_c^2}\left(Y_{cg}^2 - Y_{cg}\right)] = \text{Var}[X_{cg}^2] + \frac{4c_{\gamma,-1}}{n_{\text{reads}}}M_3 + \frac{2c_{\gamma,-2}}{n_{\text{reads}}^2}M_2 \tag{102}$$
$$= 2(2r+3)(r+1)r\theta^4 + \frac{4c_{\gamma,-1}}{n_{\text{reads}}}r(r+1)(r+2)\theta^3 + \frac{2c_{\gamma,-2}}{n_{\text{reads}}^2}r(r+1)\theta^2. \tag{103}$$

The EB estimator for the gamma parameters can be written as $(\hat{\theta}^{\text{EB}}, \hat{r}^{\text{EB}}) = h(\hat{M}_1^{\text{EB}}, \hat{M}_2^{\text{EB}})$, with the function

$$h(x,y) = (\frac{y}{x} - x, \frac{x^2}{y-x^2}), \tag{104}$$

whose Jacobian

$$J(x,y) = \begin{bmatrix} -\frac{y}{x^2} - 1 & \frac{1}{x} \\ \frac{2xy}{(y-x^2)^2} & -\frac{x^2}{(y-x^2)^2} \end{bmatrix}, \quad J(M_1,M_2) = \begin{bmatrix} -\frac{2r+1}{r} & \frac{1}{r\theta} \\ \frac{2(r+1)}{\theta} & -\frac{1}{\theta^2} \end{bmatrix}. \tag{105}$$

Let $\tilde{\Sigma} = J\Sigma J^T$. Using the delta method we have

$$\sqrt{n_{\text{cells}}}\begin{bmatrix} \hat{\theta}^{\text{EB}} - \theta \\ \hat{r}^{\text{EB}} - r \end{bmatrix} \sim \mathcal{N}\left(\begin{bmatrix} 0 \\ 0 \end{bmatrix}, \tilde{\Sigma}\right), \tag{106}$$

with the diagonal elements of $\tilde{\Sigma}$:

$$\tilde{\Sigma}_{11} = \frac{2r+3}{r}\theta^2 + \frac{c_{\gamma,-1}}{n_{\text{reads}}}\frac{4r+5}{r}\theta + \frac{c_{\gamma,-2}}{n_{\text{reads}}^2}\frac{2(r+1)}{r} \tag{107}$$
$$\tilde{\Sigma}_{22} = 2r(r+1) + \frac{c_{\gamma,-1}}{n_{\text{reads}}}\frac{4r(r+1)}{\theta} + \frac{c_{\gamma,-2}}{n_{\text{reads}}^2}\frac{2r(r+1)}{\theta^2}. \tag{108}$$

Then the error

$$MSE(\hat{\theta}^{\text{EB}}) = \frac{1}{n_{\text{cells}}}\tilde{\Sigma}_{11} = \frac{1}{n_{\text{cells}}}\left[\frac{2r+3}{r}\theta^2 + \frac{c_{\gamma,-1}}{n_{\text{reads}}}\frac{4r+5}{r}\theta + \frac{c_{\gamma,-2}}{n_{\text{reads}}^2}\frac{2(r+1)}{r}\right] \tag{109}$$

$$MSE(\hat{r}^{\text{EB}}) = \frac{1}{n_{\text{cells}}}\tilde{\Sigma}_{22} = \frac{1}{n_{\text{cells}}}\left[2r(r+1) + \frac{c_{\gamma,-1}}{n_{\text{reads}}}\frac{4r(r+1)}{\theta} + \frac{c_{\gamma,-2}}{n_{\text{reads}}^2}\frac{2r(r+1)}{\theta^2}\right]. \tag{110}$$

Then the optimal n_{reads} for $\hat{\theta}^{\text{EB}}$ and $\hat{r}^{EB}$ can be derived as

$$n_{\text{reads}}^*(\hat{\theta}^{\text{EB}}) = \sqrt{(1-\frac{1}{2r+3})\frac{c_{\gamma,-2}}{\theta^2}}, \quad n_{\text{reads}}^*(\hat{r}^{\text{EB}}) = \sqrt{\frac{c_{\gamma,-2}}{\theta^2}}. \tag{111}$$

□

7.4 Proof of Lemma 3

Proof. **We first show** (65). Consider the tradeoff (39), any distribution P_{X_g}, and any size factor $\{\gamma_c\}_{c=1}^{n_{\text{cells}}}$ that satisfies the assumption in Lemma 3. Let $n_{\text{reads}} = \frac{\kappa}{2c_0} = O(1)$. Then $t_c \leq 2$ for all cell c and hence the estimator can be simplified as

$$\hat{p}_{0,g}^{\text{EB}}(\kappa) = \frac{1}{n_{\text{cells}}}\sum_{c=1}^{n_{\text{cells}}}(1-t_c)^{Y_{cg}}. \tag{112}$$

Next we compute the bias and the variance under this scenario.

$$bias[\hat{p}_{0,g}^{\text{EB}}(\kappa)] = \mathbb{E}[\hat{p}_{0,g}^{\text{EB}}(\kappa)] - p_{0,g}(\kappa) = \frac{1}{n_{\text{cells}}}\sum_{c=1}^{n_{\text{cells}}}\mathbb{E}\left[\mathbb{E}\left[(1-t_c)^{Y_{cg}}\middle|X_{cg}\right]\right] - p_{0,g}(\kappa) \tag{113}$$

$$= \frac{1}{n_{\text{cells}}}\sum_{c=1}^{n_{\text{cells}}}\mathbb{E}\left[\sum_{y=0}^{\infty}(1-t_c)^y\exp(-n_{\text{reads}}\gamma_c X_{cg})\frac{(n_{\text{reads}}\gamma_c X_{cg})^y}{y!}\right] - p_{0,g}(\kappa) \tag{114}$$

$$= \frac{1}{n_{\text{cells}}}\sum_{c=1}^{n_{\text{cells}}}\mathbb{E}\left[\exp(-n_{\text{reads}}\gamma_c X_{cg})\sum_{y=0}^{\infty}\frac{(-\kappa X_{cg} + n_{\text{reads}}\gamma_c X_{cg})^y}{y!}\right] - p_{0,g}(\kappa) \tag{115}$$

$$= \frac{1}{n_{\text{cells}}}\sum_{c=1}^{n_{\text{cells}}}\mathbb{E}\left[\exp(-\kappa X_{cg})\right] - p_{0,g}(\kappa) = 0, \tag{116}$$

where the third equality is by Poisson probability and the fifth by Taylor expansion. The variance

$$var[\hat{p}_{0,g}^{\text{EB}}(\kappa)] = \frac{1}{n_{\text{cells}}^2}\sum_{c=1}^{n_{\text{cells}}}\text{Var}\left[(1-t_c)^{Y_{cg}}\right] \leq \frac{1}{n_{\text{cells}}^2}\sum_{c=1}^{n_{\text{cells}}}\mathbb{E}\left[(1-t_c)^{2Y_{cg}}\right] = O\left(\frac{1}{n_{\text{cells}}}\right),$$

where the last equality is by noting that $(1-t_c)^{2Y_{cg}} \leq 1$. Then by letting $n_{\text{cells}} = \Theta(B)$, the MSE of the $\hat{p}_{0,g}^{\text{EB}}(\kappa)$

$$MSE[\hat{p}_{0,g}^{\text{EB}}(\kappa)] = bias[\hat{p}_{0,g}^{\text{EB}}(\kappa)]^2 + var[\hat{p}_{0,g}^{\text{EB}}(\kappa)] = O\left(\frac{1}{B}\right).$$

We next show (66). Again consider the tradeoff (39), any distribution $P_{X_{g_1g_2}}$, and any size factor $\{\gamma_c\}_{c=1}^{n_{\text{cells}}}$ that satisfies the assumption in Lemma 3. Let $n_{\text{reads}} = \frac{\kappa}{2c_0} = O(1)$. Then $t_c \leq 2$ for all cell c and hence the estimator can be simplified as

$$\hat{p}_{0,g_1g_2}^{\text{EB}}(\kappa) = \frac{1}{n_{\text{cells}}}\sum_{c=1}^{n_{\text{cells}}}(1-t_c)^{Y_{cg_1}+Y_{cg_2}}. \tag{117}$$

Next we compute the bias and the variance under this scenario.

$$
\begin{aligned}
bias[\hat{p}^{\mathrm{EB}}_{0,g_1g_2}(\kappa)] &= \mathbb{E}[\hat{p}^{\mathrm{EB}}_{0,g_1g_2}(\kappa)] - p_{0,g_1g_2}(\kappa)\\
&= \frac{1}{n_{\text{cells}}}\sum_{c=1}^{n_{\text{cells}}}\mathbb{E}\left[\mathbb{E}\left[(1-t_c)^{Y_{cg_1}+Y_{cg_2}}\middle| X_{cg_1}, X_{cg_2}\right]\right] - p_{0,g_1g_2}(\kappa)\\
&= \frac{1}{n_{\text{cells}}}\sum_{c=1}^{n_{\text{cells}}}\mathbb{E}\left[\mathbb{E}\left[(1-t_c)^{Y_{cg_1}}\middle| X_{cg_1}\right]\mathbb{E}\left[(1-t_c)^{Y_{cg_2}}\middle| X_{cg_2}\right]\right] - p_{0,g_1g_2}(\kappa)\\
&= \frac{1}{n_{\text{cells}}}\sum_{c=1}^{n_{\text{cells}}}\mathbb{E}\left[\exp(-\kappa X_{cg_1})\exp(-\kappa X_{cg_2})\right] - p_{0,g_1g_2}(\kappa) = 0,
\end{aligned}
$$

where the third equality is by the conditional independence of Y_{cg_1} and Y_{cg_2} given X_{cg_1}, X_{cg_2}, and the fourth inequality is by the same reason as (113). The variance

$$
var[\hat{p}^{\mathrm{EB}}_{0,g_1g_2}(\kappa)] = \frac{1}{n^2_{\text{cells}}}\sum_{c=1}^{n_{\text{cells}}}\mathrm{Var}\left[(1-t_c)^{Y_{cg_1}+Y_{cg_2}}\right] \le \frac{1}{n^2_{\text{cells}}}\sum_{c=1}^{n_{\text{cells}}}\mathbb{E}\left[(1-t_c)^{2(Y_{cg}+Y_{cg_2})}\right] = O\left(\frac{1}{n_{\text{cells}}}\right),
$$

where the last equality is by noting that $(1-t_c)^{2(Y_{cg}+Y_{cg_2})} \le 1$. Then by letting $n_{\text{cells}} = \Theta(B)$, the MSE of $\hat{p}^{\mathrm{EB}}_{0,g_1g_2}(\kappa)$

$$
MSE[\hat{p}^{\mathrm{EB}}_{0,g_1g_2}(\kappa)] = bias[\hat{p}^{\mathrm{EB}}_{0,g_1g_2}(\kappa)]^2 + var[\hat{p}^{\mathrm{EB}}_{0,g_1g_2}(\kappa)] = O\left(\frac{1}{B}\right).
$$

□

7.5 Proof of Lemma 4

Proof. By the property of MLE,

$$
\mathbb{E}[\sqrt{n_{\text{cells}}}(\hat{\alpha}^{\mathrm{EB}} - \alpha)] \to 0
$$

and

$$
\mathrm{Cov}[\sqrt{n_{\text{cells}}}(\hat{\alpha}^{\mathrm{EB}} - \alpha)] \to \mathscr{I}^{-1}(\alpha).
$$

Then,

$$
\mathbb{E}\left[\|\sqrt{n_{\text{cells}}}(\hat{\alpha}^{\mathrm{EB}} - \alpha)\|_2^2\right] \to \mathrm{Tr}(\mathscr{I}^{-1}(\alpha)) = \Theta(1).
$$

Letting $n_{\text{cells}} = \Theta(B)$ and we reach that

$$
\mathbb{E}\left[\|\hat{\alpha}^{\mathrm{EB}} - \alpha\|_2^2\right] = O\left(\frac{1}{B}\right).
$$

□

7.6 Proof of Lemma 5

Proof. We use Le Cam's two-point method ([38], Chapter 2) to prove the lower bound, whose core theorem can be stated as follows:

Theorem 3. *(Le Cam's method[38]) For any family $\mathscr{P}$ of distributions for which there exists a pair $P_1, P_2 \in \mathscr{P}$ satisfying the corresponding distributional quantities $|\theta(P_1) - \theta(P_2)| \ge \delta$, then the worse-case error after n observations has the lower bound*

$$
\inf_{\hat{\theta}}\sup_{P\in\mathscr{P}}\mathbb{E}[(\hat{\theta}-\theta)^2] \ge \frac{\delta^2}{8}(1 - \|P_1^{\times n} - P_2^{\times n}\|_{TV}), \tag{118}
$$

where $P_1^{\times n}$ refers to the product distribution of n samples.

In order to use Theorem 3, we need to find a pair of distributions P_{X_g} and P'_{X_g} whose corresponding quantities to estimate—may it be the moments, the inactive probability, or the distribution parameters—differ at least by $\Omega(\frac{1}{\sqrt{B}})$, and yet their induced observation distributions P_{Y_g} and P'_{Y_g} are almost indistinguishable, as quantified by their total variation distance $\|P_{Y_g}^{\times n_{\text{cells}}} - P_{Y_g}'^{\times n_{\text{cells}}}\|_{TV}$ bounded from above by a constant that is smaller than 1.

We now construct such a distribution pair P_{X_g}, P'_{X_g} and use it to prove all results. In order to do so, this distribution pair should lie in the exponential family $\mathscr{P}^{exp}$ as defined in (67). Following the recipe in Lemma 6, first let $\{x_i\}$ be the same support as $\mathscr{X}$ which is used to discretize the exponential family distribution (67). Then there exists an α' whose corresponding $\{p_i\}$—as specified by letting $P_{X_g}(x;\alpha') = P'_{X_g}(x)$—are all in $[0,1]$ and thus satisfy the requirement in Lemma 6. Let δ be the one specified by Lemma 6. Since $\mathscr{P}^{exp}$ has one free dimension for the zero probability, there exists an α such that $P_{X_g}(x=0;\alpha) = P_{X_g}$. In other words, We know that pair of distributions that we have just constructed satisfy (125) and lie in $\mathscr{P}^{exp}$.

Since the second half of (125) has already given the indistinguishability condition, in order to use Theorem 3 to show the result, we only need to make sure that for the distribution pair P_{X_g}, P'_{X_g}, the difference of the quantity to estimate $|\theta(P_{X_g}) - \theta(P'_{X_g})| = \Theta(\frac{1}{\sqrt{B}})$ in each case. Next we examine the statement in Lemma 5 line by line, where we recall that by the first half of (125) $\delta = \Theta(\frac{1}{\sqrt{B}})$.

First, for the kth-moment (70),

$$|M_k(P_{X_g}) - M_k(P'_{X_g})| = \delta \sum_{i=1}^{m} p_i x_i^k = \Theta\left(\frac{1}{\sqrt{B}}\right). \tag{119}$$

Second, for the pairwise moment (71), let us augment a second dimension by defining the distribution pair to be $P_{X_{g_1 g_2}} = P_{X_g} \times P_{X_{g_2}}$ and $P'_{X_{g_1 g_2}} = P'_{X_g} \times P_{X_{g_2}}$ for $P_{X_{g_2}}$ that takes value x_1 with probability 1. Since the second dimension is constant and is independent of the first dimension, the indistinguishability condition still holds. At the same time,

$$|M_{11}(P_{X_g}) - M_{11}(P'_{X_g})| = \delta \sum_{i=1}^{m} p_i x_i x_1 = \Theta\left(\frac{1}{\sqrt{B}}\right). \tag{120}$$

Third, for the inactive probability (72),

$$|p_0(\kappa)(P_{X_g}) - p_0(\kappa)(P'_{X_g})| = \delta\left(1 - \sum_{i=1}^{m} p_i \exp(-\kappa x_i)\right) = \Theta\left(\frac{1}{\sqrt{B}}\right). \tag{121}$$

Fourth, for the pairwise inactive probability (73), augment the second dimension the same way as we did for the pairwise moment. Then

$$|p_0(\kappa)(P_{X_{g_1 g_2}}) - p_0(\kappa)(P'_{X_{g_1 g_2}})| = \delta \exp(-\kappa x_1)\left(1 - \sum_{i=1}^{m} p_i \exp(-\kappa x_i)\right) = \Theta\left(\frac{1}{\sqrt{B}}\right). \tag{122}$$

Finally, for the distribution (74), since the zero probability is a continuous function of α_0, the first element of α, then $|\alpha_0 - \alpha_0'| = \Theta(\delta) = \Theta(\frac{1}{\sqrt{B}})$. Then we complete the proof by noting that the MSE of estimating α is lower bounded by the MSE of estimating its first element. □

7.7 Lemma 6 with proof

Lemma 6. *(Auxiliary lemma for the proof of Lemma 5) Let $\{p_i, x_i\}_{i=1}^m$ be any ensemble satisfying $\sum_{i=1}^m p_i = 1$, $\forall i$, $0 \le p_i \le 1$, $x_i > 0$. For any tradeoff $(n_{\text{reads}}, n_{\text{cells}})$ such that $n_{\text{reads}} n_{\text{cells}} = B$, there exists $\delta \le \frac{1}{2}$ such that the distribution pair P_{X_g}, P'_{X_g} defined by*

$$P_{X_g}(x) = \left(\frac{1}{2} - \delta\right)\mathbb{I}_{\{x=0\}} + \left(\frac{1}{2} + \delta\right)\sum_{i=1}^{m} p_i \mathbb{I}_{\{x=x_i\}} \tag{123}$$

$$P'_{X_g}(x) = \frac{1}{2}\mathbb{I}_{\{x=0\}} + \frac{1}{2}\sum_{i=1}^{m} p_i \mathbb{I}_{\{x=x_i\}} \tag{124}$$

satisfies

$$\delta = \Theta(\frac{1}{\sqrt{B}}), \quad \|P_{Y_g}^{\times n_{\text{cells}}} - P_{Y_g}^{\prime \times n_{\text{cells}}}\|_{TV} \leq \frac{1}{2}, \tag{125}$$

where P_{Y_g} and P'_{Y_g} are the corresponding observation distributions.

Proof. (Proof of Lemma 6) The observation distributions can be written as

$$P_{Y_g}(y) = \left(\frac{1}{2} - \delta\right)\mathbb{I}_{\{y=0\}} + \left(\frac{1}{2} + \delta\right)\sum_{i=1}^{m} p_i poi(y, n_{\text{reads}} x_i) \tag{126}$$

$$P'_{Y_g}(y) = \frac{1}{2}\mathbb{I}_{\{y=0\}} + \frac{1}{2}\sum_{i=1}^{m} p_i poi(y, n_{\text{reads}} x_i), \tag{127}$$

where $poi(y, \lambda)$ represents that probability that $Y = y$ when Y follows a Poisson distribution with rate λ, i.e., $Y \sim \text{Poi}(n_{\text{reads}} x_i)$. Define

$$\varepsilon = 1 - \sum_{i=1}^{m} p_i \exp(-n_{\text{reads}} x_i). \tag{128}$$

Then we have

$$P_{Y_g}(0) = \left(\frac{1}{2} - \delta\right) + \left(\frac{1}{2} + \delta\right)\sum_{i=1}^{m} p_i \exp(-n_{\text{reads}} x_i) = \tag{129}$$

$$= 1 - \frac{1}{2} + \frac{1}{2}\sum_{i=1}^{m} p_i \exp(-n_{\text{reads}} x_i) - \delta + \delta\sum_{i=1}^{m} p_i \exp(-n_{\text{reads}} x_i) = 1 - \frac{1}{2}\varepsilon - \delta\varepsilon. \tag{130}$$

Similarly

$$\sum_{y=1}^{\infty} P_{Y_g}(y) = \frac{1}{2}\varepsilon + \delta\varepsilon, \quad P'_{Y_g}(0) = 1 - \frac{1}{2}\varepsilon, \quad \sum_{y=1}^{\infty} P'_{Y_g}(y) = \frac{1}{2}\varepsilon. \tag{131}$$

Then the KL divergence between P_{Y_g} and P'_{Y_g} can be computed as

$$D_{KL}(P_{Y_g}\|P'_{Y_g}) = \sum_{y=0}^{\infty} P_{Y_g}(y)\log\frac{P_{Y_g}(y)}{P'_{Y_g}(y)} = P_{Y_g}(0)\log\frac{P_{Y_g}(0)}{P'_{Y_g}(0)} + \sum_{y=1}^{\infty} P_{Y_g}(y)\log(1+2\delta) \tag{132}$$

$$= \left(1 - \frac{1}{2}\varepsilon - \delta\varepsilon\right)\log\frac{1 - \frac{1}{2}\varepsilon - \delta\varepsilon}{1 - \frac{1}{2}\varepsilon} + \left(\frac{1}{2}\varepsilon + \delta\varepsilon\right)\log(1+2\delta) \tag{133}$$

$$= \left(1 - \frac{1}{2}\varepsilon - \delta\varepsilon\right)\log\left(1 - \frac{\delta\varepsilon}{1 - \frac{1}{2}\varepsilon}\right) + \left(\frac{1}{2}\varepsilon + \delta\varepsilon\right)\log(1+2\delta) \tag{134}$$

$$= \left(1 - \frac{1}{2}\varepsilon - \delta\varepsilon\right)\left[-\frac{\delta\varepsilon}{1 - \frac{1}{2}\varepsilon} - \frac{1}{2}\left(\frac{\delta\varepsilon}{1 - \frac{1}{2}\varepsilon}\right)^2 + o(\delta^2\varepsilon^2)\right] + \left(\frac{1}{2}\varepsilon + \delta\varepsilon\right)\left[2\delta - 2\delta^2 + o(\delta^2)\right] \tag{135}$$

$$= \frac{1}{2}\frac{(\delta\varepsilon)^2}{1 - \frac{1}{2}\varepsilon} + o(\delta^2\varepsilon^2) + \delta^2\varepsilon + o(\delta^2\varepsilon) = \left[\delta^2\varepsilon + \frac{1}{2}\frac{(\delta\varepsilon)^2}{1 - \frac{1}{2}\varepsilon}\right](1 + o(1)), \tag{136}$$

where the fifth equality is by the Taylor expansion $\log(1+x) = x - \frac{x^2}{2} + o(x^2)$ for $x = o(1)$. Next we consider the total variation distance of the product distribution

$$\|P_{Y_g}^{\times n_{\text{cells}}} - P_{Y_g}^{\prime \times n_{\text{cells}}}\|_{TV}^2 \leq \frac{n_{\text{cells}}}{2} D_{KL}(P_{Y_g}\|P'_{Y_g}) = \left[\frac{n_{\text{cells}}\delta^2\varepsilon}{2} + \frac{n_{\text{cells}}}{4}\frac{(\delta\varepsilon)^2}{1 - \frac{1}{2}\varepsilon}\right](1 + o(1)). \tag{137}$$

Now let $\delta = \frac{c_0}{\sqrt{B}}$ for some constant c_0 to be specified later. Then

$$||P_{Y_g}^{\times n_{\text{cells}}} - P_{Y_g}'^{\times n_{\text{cells}}}||_{TV}^2 \leq \left[\frac{c_0^2 \varepsilon}{2n_{\text{reads}}} + \frac{(c_0\varepsilon)^2}{4n_{\text{reads}}\left(1-\frac{1}{2}\varepsilon\right)}\right](1+o(1)). \tag{138}$$

When $n_{\text{reads}} = o(1)$,

$$\varepsilon = 1 - \sum_{i=1}^{m} p_i \exp(-n_{\text{reads}} x_i) = \Theta(n_{\text{reads}} \sum_{i=1}^{m} p_i x_i) = \Theta(n_{\text{reads}}). \tag{139}$$

When $n_{\text{reads}} = \Omega(1)$,

$$\varepsilon = 1 - \sum_{i=1}^{m} p_i \exp(-n_{\text{reads}} x_i) = \Theta(1). \tag{140}$$

In both cases,

$$||P_{Y_g}^{\times n_{\text{cells}}} - P_{Y_g}'^{\times n_{\text{cells}}}||_{TV}^2 = c_0^2 \Theta(1), \tag{141}$$

378 and hence there exists a c_0 such that $||P_{Y_g}^{\times n_{\text{cells}}} - P_{Y_g}'^{\times n_{\text{cells}}}||_{TV} \leq \frac{1}{2}$. Hence, we have completed the proof. □

References

1. J. Ding, X. Adiconis, S. K. Simmons, M. S. Kowalczyk, C. C. Hession, N. D. Marjanovic, T. K. Hughes, M. H. Wadsworth, T. Burks, L. T. Nguyen, *et al.*, "Systematic comparative analysis of single cell rna-sequencing methods," *Preprint at https://www.biorxiv.org/content/10.1101/632216v2*, (2019).

2. F. A. Wolf, P. Angerer, and F. J. Theis, "Scanpy: large-scale single-cell gene expression data analysis," *Genome biology*, vol. 19, no. 1, p. 15, 2018.

3. G. X. Zheng, J. M. Terry, P. Belgrader, P. Ryvkin, Z. W. Bent, R. Wilson, S. B. Ziraldo, T. D. Wheeler, G. P. McDermott, J. Zhu, *et al.*, "Massively parallel digital transcriptional profiling of single cells," *Nature communications*, vol. 8, p. 14049, 2017.

4. A. Orlitsky, A. T. Suresh, and Y. Wu, "Optimal prediction of the number of unseen species," *Proceedings of the National Academy of Sciences*, vol. 113, no. 47, pp. 13283–13288, 2016.

5. M. I. Love, W. Huber, and S. Anders, "Moderated estimation of fold change and dispersion for RNA-seq data with DESeq2," *Genome biology*, vol. 15, no. 12, p. 550, 2014.

6. J. K. Kim, A. A. Kolodziejczyk, T. Ilicic, S. A. Teichmann, and J. C. Marioni, "Characterizing noise structure in single-cell RNA-seq distinguishes genuine from technical stochastic allelic expression," *Nature communications*, vol. 6, p. 8687, 2015.

7. W. Chen, Y. Li, J. Easton, D. Finkelstein, G. Wu, and X. Chen, "UMI-count modeling and differential expression analysis for single-cell RNA sequencing," *Genome biology*, vol. 19, no. 1, p. 70, 2018.

8. T. N. Vu, Q. F. Wills, K. R. Kalari, N. Niu, L. Wang, M. Rantalainen, and Y. Pawitan, "Beta-poisson model for single-cell RNA-seq data analyses," *Bioinformatics*, vol. 32, no. 14, pp. 2128–2135, 2016.

9. E. Pierson and C. Yau, "Zifa: Dimensionality reduction for zero-inflated single-cell gene expression analysis," *Genome biology*, vol. 16, no. 1, p. 241, 2015.

10. B. Efron, "Two modeling strategies for empirical Bayes estimation," *Statistical science: a review journal of the Institute of Mathematical Statistics*, vol. 29, no. 2, p. 285, 2014.

11. J. Wang, M. Huang, E. Torre, H. Dueck, S. Shaffer, J. Murray, A. Raj, M. Li, and N. R. Zhang, "Gene expression distribution deconvolution in single-cell RNA sequencing," *Proceedings of the National Academy of Sciences*, vol. 115, no. 28, pp. E6437–E6446, 2018.

12. L. Wang, Z. Feng, X. Wang, X. Wang, and X. Zhang, "DEGseq: an R package for identifying differentially expressed genes from RNA-seq data," *Bioinformatics*, vol. 26, no. 1, pp. 136–138, 2009.

13. K. D. Korthauer, L.-F. Chu, M. A. Newton, Y. Li, J. Thomson, R. Stewart, and C. Kendziorski, "A statistical approach for identifying differential distributions in single-cell RNA-seq experiments," *Genome biology*, vol. 17, no. 1, p. 222, 2016.

14. I. T. Jolliffe, "Principal component analysis and factor analysis," in *Principal component analysis*, pp. 115–128, Springer, 1986.

15. A. Abid, M. J. Zhang, V. K. Bagaria, and J. Zou, "Exploring patterns enriched in a dataset with contrastive principal component analysis," *Nature communications*, vol. 9, no. 1, p. 2134, 2018.

16. J. Shi and J. Malik, "Normalized cuts and image segmentation," *IEEE Transactions on pattern analysis and machine intelligence*, vol. 22, no. 8, pp. 888–905, 2000.

17. J. Friedman, T. Hastie, and R. Tibshirani, "Sparse inverse covariance estimation with the graphical lasso," *Biostatistics*, vol. 9, no. 3, pp. 432–441, 2008.

18. B. Zhang and S. Horvath, "A general framework for weighted gene co-expression network analysis," *Statistical applications in genetics and molecular biology*, vol. 4, no. 1, 2005.

19. S. Mohammadi, J. Davila-Velderrain, M. Kellis, and A. Grama, "DECODE-ing sparsity patterns in single-cell RNA-seq," *Preprint at https://www.biorxiv.org/content/10.1101/241646v2*, (2018).

20. M. D. Robinson, D. J. McCarthy, and G. K. Smyth, "edgeR: a Bioconductor package for differential expression analysis of digital gene expression data," *Bioinformatics*, vol. 26, no. 1, pp. 139–140, 2010.

21. M. D. Robinson and G. K. Smyth, "Moderated statistical tests for assessing differences in tag abundance," *Bioinformatics*, vol. 23, no. 21, pp. 2881–2887, 2007.

22. E. Shapiro, T. Biezuner, and S. Linnarsson, "Single-cell sequencing-based technologies will revolutionize whole-organism science," *Nature Reviews Genetics*, vol. 14, no. 9, p. 618, 2013.

23. V. Svensson, K. N. Natarajan, L.-H. Ly, R. J. Miragaia, C. Labalette, I. C. Macaulay, A. Cvejic, and S. A. Teichmann, "Power analysis of single-cell RNA-sequencing experiments," *Nature methods*, vol. 14, no. 4, p. 381, 2017.

24. G. Heimberg, R. Bhatnagar, H. El-Samad, and M. Thomson, "Low dimensionality in gene expression data enables the accurate extraction of transcriptional programs from shallow sequencing," *Cell systems*, vol. 2, no. 4, pp. 239–250, 2016.

25. J. Jiao, K. Venkat, Y. Han, and T. Weissman, "Minimax estimation of functionals of discrete distributions," *IEEE Transactions on Information Theory*, vol. 61, no. 5, pp. 2835–2885, 2015.

26. Y. Wu, P. Yang, *et al.*, "Chebyshev polynomials, moment matching, and optimal estimation of the unseen," *The Annals of Statistics*, vol. 47, no. 2, pp. 857–883, 2019.

27. W. Kong, G. Valiant, *et al.*, "Spectrum estimation from samples," *The Annals of Statistics*, vol. 45, no. 5, pp. 2218–2247, 2017.

28. I. Good and G. Toulmin, "The number of new species, and the increase in population coverage, when a sample is increased," *Biometrika*, vol. 43, no. 1-2, pp. 45–63, 1956.

29. B. Efron and R. Thisted, "Estimating the number of unseen species: How many words did Shakespeare know?," *Biometrika*, vol. 63, no. 3, pp. 435–447, 1976.

30. B. Efron, "Empirical Bayes deconvolution estimates," *Biometrika*, vol. 103, no. 1, pp. 1–20, 2016.

31. A. M. Klein, L. Mazutis, I. Akartuna, N. Tallapragada, A. Veres, V. Li, L. Peshkin, D. A. Weitz, and M. W. Kirschner, "Droplet barcoding for single-cell transcriptomics applied to embryonic stem cells," *Cell*, vol. 161, no. 5, pp. 1187–1201, 2015.

32. J. C. Marioni, C. E. Mason, S. M. Mane, M. Stephens, and Y. Gilad, "RNA-seq: an assessment of technical reproducibility and comparison with gene expression arrays," *Genome research*, vol. 18, no. 9, pp. 1509–1517, 2008.

33. M. D. Robinson and G. K. Smyth, "Small-sample estimation of negative binomial dispersion, with applications to sage data," *Biostatistics*, vol. 9, no. 2, pp. 321–332, 2007.

34. S. Anders and W. Huber, "Differential expression analysis for sequence count data," *Genome biology*, vol. 11, no. 10, p. R106, 2010.

35. A. B. Tsybakov, "Introduction to nonparametric estimation. revised and extended from the 2004 french original. translated by vladimir zaiats," 2009.

36. A. W. Van der Vaart, *Asymptotic statistics*, vol. 3. Cambridge university press, 2000.

37. G. Valiant and P. Valiant, "Estimating the unseen: an n/log (n)-sample estimator for entropy and support size, shown optimal via new clts," in *Proceedings of the forty-third annual ACM symposium on Theory of computing*, pp. 685–694, ACM, 2011.

38. L. Le Cam, *Asymptotic methods in statistical decision theory*. Springer Science & Business Media, 2012.

39. E. Jones, T. Oliphant, P. Peterson, *et al.*, "Scipy: Open source scientific tools for python," 2001.

40. B. BioSciences, “Human and mouse cd marker handbook,” 2017.

41. H. Mi, X. Huang, A. Muruganujan, H. Tang, C. Mills, D. Kang, and P. D. Thomas, “Panther version 11: expanded annotation data from gene ontology and reactome pathways, and data analysis tool enhancements,” *Nucleic acids research*, vol. 45, no. D1, pp. D183–D189, 2016.

42. D. Chaussabel, C. Quinn, J. Shen, P. Patel, C. Glaser, N. Baldwin, D. Stichweh, D. Blankenship, L. Li, I. Munagala, *et al.*, “A modular analysis framework for blood genomics studies: application to systemic lupus erythematosus,” *Immunity*, vol. 29, no. 1, pp. 150–164, 2008.

43. E. Z. Macosko, A. Basu, R. Satija, J. Nemesh, K. Shekhar, M. Goldman, I. Tirosh, A. R. Bialas, N. Kamitaki, E. M. Martersteck, *et al.*, “Highly parallel genome-wide expression profiling of individual cells using nanoliter droplets,” *Cell*, vol. 161, no. 5, pp. 1202–1214, 2015.

44. D. Szklarczyk, A. Franceschini, S. Wyder, K. Forslund, D. Heller, J. Huerta-Cepas, M. Simonovic, A. Roth, A. Santos, K. P. Tsafou, *et al.*, “String v10: protein–protein interaction networks, integrated over the tree of life,” *Nucleic acids research*, vol. 43, no. D1, pp. D447–D452, 2014.

45. R. M. Gorczynski, Z. Chen, D. A. Clark, J. Hu, G. Yu, X. Li, W. Tsang, and S. Hadidi, “Regulation of gene expression of murine md-1 regulates subsequent t cell activation and cytokine production,” *The Journal of Immunology*, vol. 165, no. 4, pp. 1925–1932, 2000.

46. X. Xiong, Y. Liu, Y. Mei, J. Peng, Z. Wang, B. Kong, P. Zhong, L. Xiong, D. Quan, Q. Li, *et al.*, “Novel protective role of myeloid differentiation 1 in pathological cardiac remodelling,” *Scientific reports*, vol. 7, p. 41857, 2017.
